# The Genetic Legacy of Religious Diversity and Intolerance: Paternal Lineages of Christians, Jews, and Muslims in the Iberian Peninsula

**DOI:** 10.1016/j.ajhg.2008.11.007

**Published:** 2008-12-05

**Authors:** Susan M. Adams, Elena Bosch, Patricia L. Balaresque, Stéphane J. Ballereau, Andrew C. Lee, Eduardo Arroyo, Ana M. López-Parra, Mercedes Aler, Marina S. Gisbert Grifo, Maria Brion, Angel Carracedo, João Lavinha, Begoña Martínez-Jarreta, Lluis Quintana-Murci, Antònia Picornell, Misericordia Ramon, Karl Skorecki, Doron M. Behar, Francesc Calafell, Mark A. Jobling

**Affiliations:** 1 Department of Genetics, University of Leicester, University Road, Leicester LE1 7RH, UK; 2 Laboratorio de Genética Forense y Genética de Poblaciones, Departamento de Toxicología y Legislación Sanitaria, Facultad de Medicina, Universidad Complutense de Madrid, 28040 Madrid, Spain; 3 Unidad Docente de Medicina Legal, Sección de Biología Forense, Facultad de Medicina, Universidad de Valencia, 46010 Valencia, Spain; 4 Instituto de Medicina Legal, Universidade de Santiago, Fundación de Medicina Xenómica–Hospital Clínico Universitario, 15706 Santiago de Compostela, Spain; 5 Centro de Genética Humana, Instituto Nacional de Saúde Dr. Ricardo Jorge, Av. Padre Cruz, 1649-016 Lisboa, Portugal; 6 Unidad Docente de Medicina Legal y Forense, Universidad de Zaragoza, 50.009, Zaragoza, Spain; 7 Unit of Human Evolutionary Genetics, CNRS URA3012, Institut Pasteur, 75015, Paris, France; 8 Laboratori de Genètica, IUNICS i Departament Biologia, Universitat de les Illes Balears, 07122 Palma de Mallorca, Spain; 9 Molecular Medicine Laboratory, Rambam Health Care Campus, Haifa 31096, Israel; 10 Rappaport Faculty of Medicine and Research Institute, Technion, Israel Institute of Technology, Haifa 31096, Israel; 11 Institute of Evolutionary Biology (UPF-CSIC), Departament de Ciències Experimentals i de la Salut, Universitat Pompeu Fabra, 08003 Barcelona, Spain

## Abstract

Most studies of European genetic diversity have focused on large-scale variation and interpretations based on events in prehistory, but migrations and invasions in historical times could also have had profound effects on the genetic landscape. The Iberian Peninsula provides a suitable region for examination of the demographic impact of such recent events, because its complex recent history has involved the long-term residence of two very different populations with distinct geographical origins and their own particular cultural and religious characteristics—North African Muslims and Sephardic Jews. To address this issue, we analyzed Y chromosome haplotypes, which provide the necessary phylogeographic resolution, in 1140 males from the Iberian Peninsula and Balearic Islands. Admixture analysis based on binary and Y-STR haplotypes indicates a high mean proportion of ancestry from North African (10.6%) and Sephardic Jewish (19.8%) sources. Despite alternative possible sources for lineages ascribed a Sephardic Jewish origin, these proportions attest to a high level of religious conversion (whether voluntary or enforced), driven by historical episodes of social and religious intolerance, that ultimately led to the integration of descendants. In agreement with the historical record, analysis of haplotype sharing and diversity within specific haplogroups suggests that the Sephardic Jewish component is the more ancient. The geographical distribution of North African ancestry in the peninsula does not reflect the initial colonization and subsequent withdrawal and is likely to result from later enforced population movement—more marked in some regions than in others—plus the effects of genetic drift.

## Introduction

The genetic diversity of human populations in Europe has been the subject of intense scrutiny since the first “classical” markers became available.^1^ Most studies have focused on the identification of large-scale variation and its interpretation in terms of major events in prehistory, such as expansions from glacial refugia in the Paleolithic era^2^, ^3^, ^4^, ^5^ and the spread of agriculture from the Near East in the Neolithic era.^6^, ^7^, ^8^, ^9^, ^10^, ^11^, ^12^, ^13^ This approach seems reasonable, given that early events that occurred when populations were small are likely to have had major effects that could persist to the present day. However, Europe has also been subject to migrations and invasions within historical times, and these may have played an important role in shaping current patterns of diversity^14^ and could contribute to confusion over more ancient population movement.

Although evidence of the cultural impact of historical events can be gleaned from sources such as archaeology, place names, and linguistic elements, there is often debate about the weight of their corresponding demographic impact. Genetic analysis of modern populations can offer a more direct approach to recognizing the impact of migrations and invasions in historical times, especially when source populations for migrations are clearly differentiated from recipient populations. The Iberian Peninsula is of particular interest in this context, because it has a complex recent history over the last two millennia, involving the long-term residence of two very different populations with very distinct geographical origins and their own particular cultural and religious characteristics—North African Muslims and Sephardic Jews.^15^

North Africa and the Iberian Peninsula are separated by a mere 15 km of water at the Gibraltar Strait, making the region a potential migration route between Africa and Europe. Historically documented contact began dramatically in 711 CE, when a Berber army under Arab leadership crossed from Morocco, winning a key battle the following year.^16^ Within only four years, the invaders had conquered the entire peninsula, with the exception of the northern Basque country, Cantabria, Galicia, Asturias, and most of the Pyrenees in the north, which remained largely unoccupied.^17^ Arab and Berber forces then remained in control for more than five centuries, with a gradual withdrawal toward Andalusia in the south and a final expulsion in 1492. Today, signs of this lengthy Islamic occupation are abundantly obvious in the place names, language, archaeology,^18^ architecture, and other cultural traits of Spain and Portugal, but its demographic impact is less clear.

The established population of the Iberian Peninsula prior to 711 CE has been estimated at 7–8 million people, ruled by about 200,000 Germanic Visigoths,^19^ who had entered from the north in the sixth century. Though the initial invading North African force was between 10,000 and 15,000 strong, the scale of subsequent migration and settlement is uncertain, with some claiming numbers in the hundreds of thousands.^20^ Islamization of the populace after the invasion was certainly rapid, but it has been argued that this reflects an exponential social process of religious conversion rather than a substantial immigration;^21^ a sizeable proportion of the indigenous population (the so-called Mozarabs) was allowed to retain its Christian practices, as a result of the religious tolerance of the Muslim rulers.^22^ There is also doubt about the extent of intermarriage between indigenous people and settlers in the early phase.^20^ After the overthrow of Islamic rule in most of the peninsula, a period of tolerant coexistence (*convivencia*) ensued in the twelfth and thirteenth centuries, but after 1492 (1496 in Portugal), religious intolerance forced Spanish Muslims to either convert to Christianity (as so-called *moriscos*) or leave.^23^ After the fifteenth century, *moriscos* were relocated across Spain on occasion, and, finally, during 1609–1616, over 200,000 were expelled, mostly from Valencia.

The people encountered by the Islamic invaders in the eighth century were not a religiously and culturally uniform group; they included among the Catholic Christian majority a substantial minority of Jewish people. They and their descendants are known as Sephardic Jews, from *Sepharad*, the Hebrew word for Spain. The Jewish presence was very long-established, with some evidence that it predated the Christian era; many Jews, however, are thought to have arrived during the Roman period, either voluntarily or as slaves brought from the Middle East after the defeat of Judea in 70 CE.^24^ The later arrival of others was due to their displacement by the Islamic invasion of their homelands in the Near East. Under the final years of Visigothic rule, the Jews suffered the first of a long series of persecutions, including forced religious conversion. It has been estimated that during the *convivencia*, their population size in Spain was around 100,000.^25^ In the late 14th century, a wave of pogroms affected the main Jewish quarters in Iberian cities, particularly Barcelona and Girona. One estimate^26^ gives a Spanish Jewish population of 400,000 by the time of the expulsions of the late fifteenth century, during which some 160,000 Spanish Jews were expelled, largely settling around the Mediterranean, while the remainder underwent conversion to Christianity, living as so-called *conversos* (in Spain) or *cristãos novos* (in Portugal).

Previous genetic studies of the Iberian Peninsula included analyses of classical marker frequencies,^27^, ^28^, ^29^, ^30^ autosomal *Alu* insertion polymorphisms,^31^ mitochondrial DNA variation,^32^, ^33^ and Y-chromosomal haplotypes.^34^, ^35^, ^36^, ^37^, ^38^, ^39^ In general, these surveys have paid little attention to the issue of admixture, though studies that include North African populations identify a marked genetic boundary coinciding with the Gibraltar Strait.^30^, ^31^, ^32^, ^34^, ^36^ The Y chromosome provides the phylogeographic resolution that might allow the disentangling of past admixture events,^40^ and studies^34^, ^36^, ^38^, ^41^, ^42^ have focused on haplogroup E3b2 (also known as E-M81), common in North Africa and found at an average frequency of 5.6% in the peninsula,^38^ which, adjusting for the haplogroup's frequency in North Africa itself, would correspond to a contribution of 8%–9%. Although these studies indicate the presence of some North African lineages in the Iberian Peninsula, they have taken ad hoc approaches to quantifying this and have almost entirely^41^ neglected to address the possible contribution of Sephardic Jews. Here, we take a formal admixture approach and reveal a remarkably high level of North African and Sephardic Jewish ancestry in a large sample of Y chromosomes from the Iberian Peninsula and Balearic Islands. We use the power of combined binary marker and short tandem repeat (STR) haplotyping to illuminate the relative time depths of these contributions, and we show that the geographical patterns of ancestry fail to fit simple expectations based on historical accounts, suggesting the influence of religious conversion of both Muslims and Jews and the subsequent dispersal and drift of their Y-chromosomal lineages.

## Subjects and Methods

### Subjects

A total of 1140 DNA samples isolated from males from the Iberian Peninsula and the Balearic Islands were analyzed; below, we refer to these samples as “Iberian,” for brevity. All samples were collected with appropriate ethical approval and informed consent. Individuals were assigned to geographical locations on the basis of paternal grandfather's place of birth, and they were then grouped on the basis of traditional regions. Andalusia was divided into western and eastern (including Murcia) parts; Castilla y Leon was divided into northeast and northwest Castile; and a set of individuals from the Pyrenees, including some from north of the Spain-France border, were pooled as “Gascony.” Samples from Portugal were divided into two sets, those north and those south of the Mondego river.

### Y Chromosome Haplotyping

**Figure 1:**
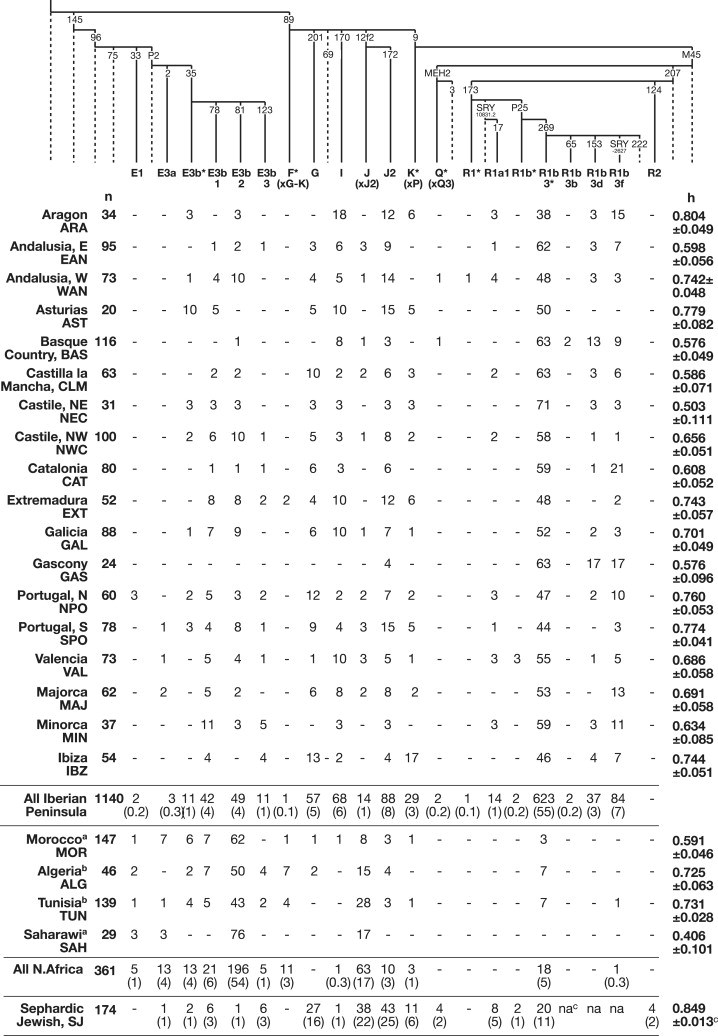
Y-Chromosomal Haplogroups in Iberian, North African, and Sephardic Jewish Samples Binary marker phylogeny of the Y chromosome, showing mutations on the branches of the tree, and shorthand haplogroup names^40^ immediately beneath. Haplogroups unobserved in any sample are indicated by dashed branches on the tree. Below the phylogeny are given the percentages of chromosomes carrying the observed haplogroup. Abbreviations are as follows: n, sample size; h, Nei's unbiased estimator of gene diversity. Data on North African populations are from the literature (see footnotes). ^a^ Data from Bosch et al.^34^ ^b^ Data from Arredi et al.,^47^ with haplogroup prediction for hgG. ^c^ Subhaplogroups of R1b3 were not typed in the Sephardic Jewish sample.

Binary markers (Figure 1) on the nonrecombining region of the Y chromosome were typed in hierarchical multiplexes,^43^ via the SNaPshot minisequencing procedure (Applied Biosystems) and an ABI3100 Genetic Analyzer (Applied Biosystems). All samples were initially analyzed with multiplex I^43^ (containing the markers M9, M69, M89, M145, M170, M172, M201, and 12f2). Samples derived for M9 (haplogroup [hg] K) were analyzed with multiplex II^43^ (containing M17, M45, M173, M207, P25, and SRY_10831_). Two individuals derived for M45 but ancestral for M207 (hgP^∗^[xR]) were analyzed with the markers MEH2 and M3 and could thus be assigned to hgQ^∗^(xQ3). Samples derived for M173 but ancestral for SRY_10831.2_ and M17 (hgR1^∗^[xR1a]) were further analyzed with multiplex IV—which, to our knowledge, is previously unpublished—containing the markers M65, M153, M222, M269, and SRY_-2627_. Ten individuals carry reversions of the marker P25 through gene conversion,^44^ and the allelic state of this marker in these chromosomes is therefore ignored for the purposes of this study. Samples derived for M145 within multiplex I (in hgDE) were further analyzed with multiplex III^43^ (containing M33, M35, M75, M78, M81, M96, M123, and P2), and the marker M2 was also analyzed as appropriate. Previously unreported primers were designed on the basis of published information about polymorphic sites.^45^ Note that hgR2 (R-M124) is reported in Figure 1, because it was detected in the Sephardic Jewish sample (Table S1, available online), but was not typed in the Iberian samples, because all chromosomes derived for M207 (hgR) were also derived for M173 (hgR1).

Nomenclature of haplogroups is in accordance with the Y Chromosome Consortium,^45^ uses updated names,^40^ and is given in Figure 1. We employ shorthand names as follows: E3b^∗^ refers to E3b^∗^(xE3b1, E3b2, E3b3), also known as E-M35^∗^(xM78, M81, M123); and R1b3^∗^ refers to R1b3^∗^(xR1b3b, R1b3d, R1b3f, R1b3g), also known as R-M269^∗^(xM65, M153, SRY_-2627_, M222).

Nineteen Y-chromosomal STRs (DYS19, DYS385a/b, DYS388, DYS389I, DYS389II, DYS390, DYS391, DYS392, DYS393, DYS434, DYS435, DYS436, DYS437, DYS438, DYS439, DYS460, DYS461, DYS462) were typed in three multiplexes, as described previously,^46^ on an ABI3100 Genetic Analyzer (Applied Biosystems). Allele nomenclature is as reported by Bosch et al.,^46^ and DYS385a and DYS385b were omitted from statistical analyses.

### Comparative data

Comparative data for North African populations were obtained from the literature.^34^, ^47^ For Moroccan and Saharawi data,^34^ haplogroup resolution was increased to facilitate comparison with haplogroups determined here by the consideration of previously published data on the marker 12f2^48^ and by typing of the hgG-defining marker M201 on chromosomes belonging to hgF^∗^(xH,I,J,K). Haplogroup G was also undetermined in the published Algerian and Tunisian data,^47^ so this haplogroup was predicted from Y-STR haplotypes via a published method.^49^ We used the Bayesian and support vector machine (SVM) approaches, with our Iberian sample as the training set, and we based the predictions on the 14 Y-STRs (the list above, omitting DYS385a/b, and DYS460-462) that are shared between our data and the published data.^47^ A single Algerian chromosome among the ten hgF^∗^(xH,I,J,K) cases was predicted with high confidence to belong to hgG (100% [Bayesian] and 96% [SVM]); this low level of the haplogroup in North Africa is consistent with the Moroccan and Saharawi samples and with an independently published set of Algerian data.^50^ In comparisons among Iberian samples typed here, 17 Y-STRs were considered; when comparisons were done with published data on North African samples,^34^, ^47^ the number of Y-STRs was reduced to eight for compatibility with the published data, after adjustment of allele nomenclature for DYS389I.^47^

Comparative data for Sephardic Jewish populations were extracted from a large collection of Y haplotypes assembled by D.M.B. and K.S. The term “Sephardic Jews” is used here in its narrow sense,^51^ referring to Jewish men deriving from originally Ladino-speaking communities that emanated directly from the Iberian Exile. Included males noted in their informed consents that they, their fathers, and their paternal grandfathers are Sephardic Jews from the specified community. A sample of 174 males was compiled (Table S1), made up of self-defined Sephardic Jewish males either from the Iberian Peninsula itself or from countries that received major migrations of Sephardic Jews after the expulsion of 1492–1496, as follows: Belmonte, Portugal (16); Bulgaria (49); Djerba (13); Greece (2); Spain (3); Turkey (91). Countries that received exiles from the Iberian Peninsula but that themselves had substantial preexisting Jewish communities (Italy and the North African countries) were not included. Haplogroups were equivalent to those typed in the Iberian Peninsula samples, except that sublineages of hgR1b3 were not defined. In haplogroup comparisons, therefore, all of these sublineages were combined into hgR1b3 (also known as R-M269) itself. Data on eight Y-STRs were available, allowing comparison with Iberian and North African data.

### Data Analysis

In many cases, sample sizes for haplogroup-based analyses are larger than those used for the same populations for Y-STR-based analyses. For the Moroccan sample (total n = 147), this is because only a subset (n = 104) was typed for Y-STRs. For the remainder, small reductions in sample sizes are due to the removal of chromosomes carrying STR-allele duplications, or “partial” alleles, which cannot be readily accommodated in STR-based analyses.

Summary statistics (Nei's estimator of gene diversity, population-pairwise *F*_ST_ [for haplogroups] and *R*_ST_ [for Y-STR haplotypes]) were calculated with Arlequin.^52^ Multidimensional scaling based on *F*_ST_ and *R*_ST_ matrices was carried out with PROXSCAL in SPSS 14.0.

Relationships between Y-STR haplotypes within specific haplogroups were displayed via reduced-median networks^53^ constructed within Network 4.500 with the use of intrahaplogroup variance-based weighting as described previously.^54^ Chromosomes carrying Y-STR allele duplications, or partial alleles, were omitted before analysis.

Admixture proportions were estimated with mY statistics implemented in Admix 2.0.^55^ This coalescent-based estimator takes into account allele frequencies, as well as molecular information.^56^ All potential parental populations are expected to be sampled and constant in size, and the effects of genetic drift or gene flow since the admixture event are considered negligible.^11^ We have used three parental populations: Basques (n = 115), Moroccans^34^ (n = 104), and Sephardic Jews (n = 174). Molecular distances between haplotypes were calculated with binary markers and microsatellites, weighted respectively at 100 and 1 to reflect their differences in mutation rates. Standard errors were calculated on the basis of 10,000 bootstraps.

Average square difference (ASD) was calculated as described previously.^57^

## Results

A total of 30 binary markers were typed in a set of 1140 Y chromosomes belonging to 18 populations from the Iberian Peninsula and the Balearic Islands (Figure 1). Of the 31 possible haplogroups defined by these markers, 20 were observed, but seven were represented by only one or two individuals. Thirteen haplogroups (each present at about 1% or greater overall) thus account for the vast majority of chromosomes, and one haplogroup, R1b3^∗^, is by far the most common (55%). When all chromosomes derived for the marker M269 are considered (R1b3^∗^ plus its sublineages R1b3b, R1b3d, and R1b3f), this figure approaches 66%.

To provide a context in which to consider the issue of a North African genetic contribution, we compiled haplogroup frequency data for four North African populations: Moroccans and Saharawi,^34^ plus Algerians and Tunisians^47^ (Figure 1). The most common haplogroup among North African populations is E3b2, representing 54% of the total of 361 chromosomes. To consider the contribution of Sephardic Jewish populations to the modern Iberian Peninsula, we compiled a set of 174 Y haplotypes from self-defined Sephardic males with ancestry in Mediterranean countries (see Subjects and Methods). This sample does not carry one predominant haplogroup but instead shows >15% frequencies of three haplogroups: J2, J^∗^(xJ2), and G.

**Figure 2:**
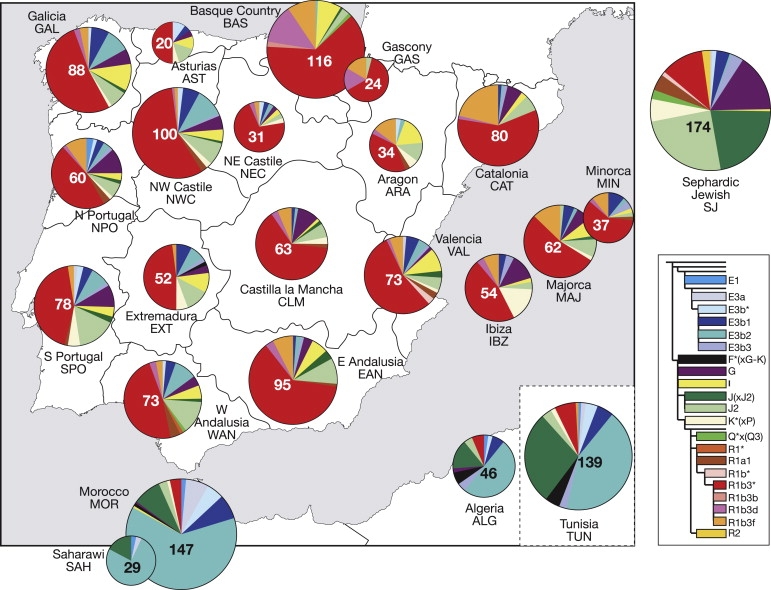
Haplogroup Distributions in Iberian, North African, and Sephardic Jewish Populations Haplogroup profiles of samples from the Iberian Peninsula and the Balearic Islands, published North African samples,^34^, ^47^ and a Sephardic Jewish sample. Sectors in pie charts are colored according to haplogroup in the schematic tree to the right, and sector areas are proportional to haplogroup frequency. Sample names, abbreviations, and sizes (within pie charts) are indicated. Subhaplogroups of R1b3 were not typed in the Sephardic Jewish sample.

Haplogroup frequencies in these Iberian, North African, and Sephardic Jewish populations are displayed graphically in Figure 2. The dramatic difference in haplogroup frequencies across the Gibraltar Strait^34^ is the most striking feature.

**Figure 3:**
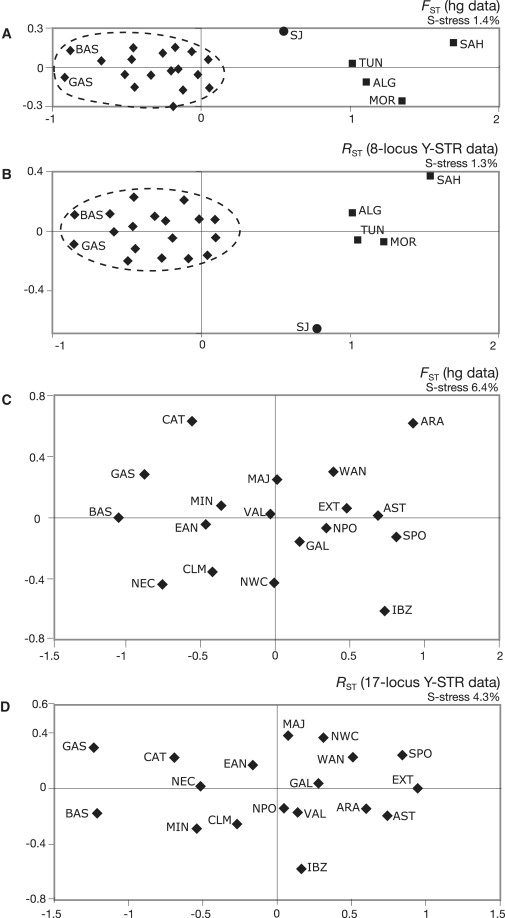
Multidimensional Scaling (MDS) Plots Illustrating the Relationships among Iberian, North African, and Sephardic Jewish Populations A) MDS plot based on population pairwise *F*_ST_ values from haplogroup data, including Iberian, North African, and Sephardic Jewish samples. All haplogroups carrying the derived allele at M269 are pooled into hgR1b3 for the purposes of this analysis. Abbreviations of population names are as in Figure 1. B) MDS plot based on population pairwise *R*_ST_ values from eight-locus Y-STR data, including Iberian, North African, and Sephardic Jewish samples. C) MDS plot based on population pairwise *F*_ST_ values from haplogroup data, including only Iberian samples. Abbreviations of population names are as in Figure 1. D) MDS plot based on population pairwise *R*_ST_ values from 17-locus Y-STR data, including only Iberian samples.

A representation of these haplogroup-frequency data in the form of a multidimensional scaling (MDS) plot based on a pairwise *F_ST_* matrix (Figure 3A) displays this distinction clearly, with the Iberian populations forming a clear cluster, the four North African populations clearly separated from them in the first dimension, and the Sephardic Jewish sample occupying an intermediate position. The Iberian populations most strongly differentiated from the non-Iberians are the Basques and the Gascons.

Ascertainment bias of the SNPs used to define haplogroups is a potential problem in Y-chromosomal diversity analysis (particularly because some Y-SNPs typed here were actually ascertained in Basques^58^, ^59^) and can be addressed by consideration of pairwise *R_ST_* estimates based on Y-STRs. Most of the Iberian samples had been previously typed with a set of 19 Y-STRs;^46^ this typing was extended to the full set of samples (Table S1). Inclusion of published data on the North African samples allowed a comparison over eight shared loci; data on the same eight loci were also available in the Sephardic Jewish sample. The MDS plot based on these data (Figure 3B) shows a similar pattern to that based on haplogroup frequencies, suggesting that ascertainment bias is not a major issue here.

Removal of the North African and Sephardic Jewish samples allows the distribution of Iberian populations to be seen more clearly (Figures 3C and 3D). Once more, the patterns based on haplogroup frequencies and Y-STR haplotypes (here based on 17 loci, with DYS385a and DYX385b removed) are broadly similar. In each case, the Basques are distinct from all other Iberian populations (and statistically significantly different, as judged by pairwise population-differentiation tests), with the exception of the Gascons, when haplogroup frequencies are considered.

To formally assess the impact of North African and Sephardic Jewish contributions on the indigenous population, we carried out admixture analysis, employing the mY estimator^55^ and treating the study populations as hybrids of three parental populations. We chose the Basques as the Iberian parental sample. This is justified on the basis of a relative absence of Muslim occupation of the Basque region^17^ and supported by the genetic distinctiveness of the Basque and neighboring Gascon samples (Figure 3). We chose the Moroccans as the North African parental sample, on the basis of historical evidence that entry to the Iberian Peninsula occurred via the Strait of Gibraltar^17^ and that the invading armies were largely native to Morocco. The third parental population was the Sephardic Jewish sample.

**Figure 4:**
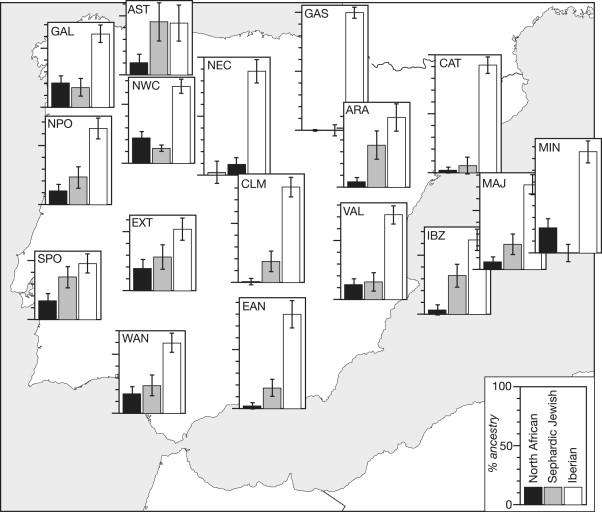
Iberian, North African, and Sephardic Jewish Admixture Proportions among Iberian Peninsula Samples Mean North African, Sephardic Jewish, and Iberian admixture proportions among Iberian samples, based on the mY estimator and on Moroccan, Sephardic Jewish, and Basque parental populations, are represented on a map as shaded bars on bar charts. Error bars indicate standard deviations, and three-letter codes indicate populations, as given in Figure 1.

Mean ancestry proportions and their standard deviations for each population are represented schematically in Figure 4 (see Table S2 also). Considering the peninsula as a single population, the analysis unsurprisingly finds that the highest mean proportion of ancestry corresponds to the Basque parental population. However, this level is only 69.6%, leaving a remarkably high overall mean proportion of North African and Jewish ancestry forming the remainder. Mean North African admixture is 10.6%, with wide geographical variation (Figure 4, Table S2), ranging from zero in Gascony to 21.7% in Northwest Castile. Mean Sephardic Jewish admixture is 19.8%, varying from zero in Minorca to 36.3% in South Portugal (the value in Asturias is unlikely to be reliable, because of small sample size).

**Figure 5:**
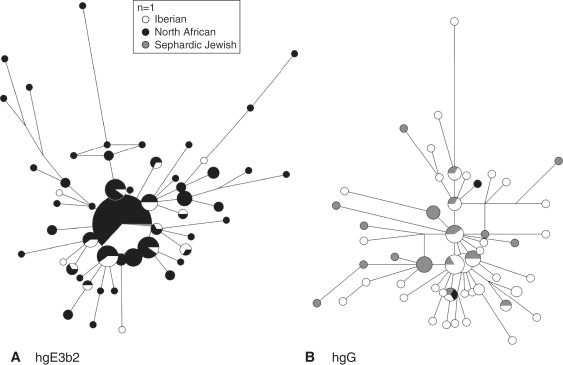
Diversity of Y-STR Haplotypes Belonging to Haplogroups E3b2 and G A) Reduced median network^53^ containing the eight-locus Y-STR (DYS19, DYS388, DYS389I, DYS389II-I, DYS390, DYS391, DYS392, DYS393) haplotypes of 170 hgE3b2 chromosomes. North African haplotypes include those from Moroccan, Algerian, Tunisian, and Saharawi samples. Circles represent haplotypes, with area proportional to frequency and colored according to population, as shown in the key. B) Analogous network for 82 chromosomes within hgG.

To examine admixture in more detail, we can compare Y-STR haplotypes within prominent lineages shared between the Iberian samples and the North African and Sephardic Jewish samples. A reduced-median network representing the eight-locus haplotypes within hgE3b2, the predominant haplogroup in North Africa, is shown in Figure 5a. The network is star-like, with a major core haplotype shared by 48 North Africans and 27 Iberians, plus the sole example of a Sephardic Jewish haplotype. In total, twelve of the 51 haplotypes are shared between North Africans and Iberians, but Iberians show a lower diversity (average squared difference [ASD] = 2.85) than North Africans (ASD = 9.13). This is consistent with a history of migration of North Africans to Iberia and introgression of hgE3b2 haplotypes, representing a subset of the North African diversity, into the indigenous population. A reciprocal example is provided by hgG (Figure 5B), frequent in the Sephardic Jewish sample. In this case, only two North African chromosomes belong to this haplogroup, but 7/48 haplotypes are shared between Sephardic Jewish and Iberian chromosomes, and the respective ASD values are similar, at 14.00 and 15.10. The high degree of haplotype sharing indicates introgression of Sephardic Jews into the indigenous Iberian population, but the similarity in haplotype diversity suggests that this was relatively ancient. Supporting a contribution of Sephardic Jewish patrilines to the Iberian population, shared STR haplotypes between the two within haplogroups E3b1, J^∗^, J2, and K^∗^ (data not shown, Table S1) were also observed. The mean proportion of identical haplotypes shared between the Sephardic Jewish sample and the Iberian samples is 3.6%, whereas the proportion for those shared between the Moroccan sample and the Iberian samples is 2.8%.

## Discussion

The Iberian Peninsula is often regarded as a source for northward postglacial expansions^2^, ^3^, ^5^ and a sphere of Neolithic influence from the Near East.^38^ Our study suggests that its recent history has also had a profound influence on its diversity of Y-chromosomal lineages. Historical accounts should allow us to account for this, but they are sometimes written long after the incidents they describe, are usually scarce, and are always recorded with a particular audience in mind (and, therefore, are subject to bias).^17^ The marked genetic differentiation between the contributing populations in this case allows an attempt to disentangle their influence; such recognition may be more difficult when source populations for migrations or invasions are only slightly differentiated from recipient populations, as in the case of the Anglo-Saxon^60^ or Viking^61^ contributions to the British Isles, for example.

Our admixture approach has identified high mean levels of North African and Sephardic Jewish patrilineal ancestry in modern populations of the Iberian Peninsula and Balearic Islands. We find a mean of 10.6% North African ancestry, somewhat higher than previous ad hoc estimates,^38^ and a mean of 19.8% Sephardic Jewish ancestry, a figure that cannot be readily compared with any other study. These findings attest to a high level of religious conversion (whether voluntary or enforced) driven by historical episodes of religious intolerance, which ultimately led to the integration of descendants.

It has been claimed that there is some archaeological evidence to support prehistoric African influence in the Iberian Peninsula,^62^ and a single mitochondrial DNA (mtDNA) haplotype of North African origin found among ancient DNA samples of Iberian Bronze Age cattle from northern Spain^63^ has been taken as support of this claim. However, we observe low diversity of the prominent North African lineage hgE3b2 in Iberian populations, which argues against a prehistoric origin for the majority of chromosomes in this lineage, the low diversity being more compatible with their arrival in more recent times.

North Africans entered the Iberian Peninsula from the south, and after a rapid northward expansion soon retreated southwards, being finally expelled from Andalusia over 700 years after their arrival. Thus, they apparently spent the least amount of time in the north, and we might therefore expect a south-north gradient of North African ancestry proportions. However (and in agreement with studies of independent samples^36^, ^41^, ^64^), we find no evidence of this. Indeed, the highest mainland proportions of North African ancestry (>20%) are found in Galicia and Northwest Castile, with much lower proportions in Andalusia. The most striking division in North African ancestry proportions is between the western half of the peninsula, where the proportion is relatively high, to the eastern half, where it is relatively low (Figure 4). This distribution could reflect genetic drift, as well as the history of enforced relocations and expulsion of *moriscos.* The entire large community of *moriscos* in Granada was relocated northward and westward following the war of 1567–1571.^23^ In addition, the final expulsion of *moriscos*, ordered by Philip III and beginning in1609, was highly effective in some regions of Spain, including Valencia and Western Andalucia, but less so in Galicia and Extremadura, where the population was more dispersed and integrated. Jewish communities were already widespread and long-established by 711 CE, so we might expect the level of Sephardic ancestry to also be widespread and undifferentiated. With the exception of the far northeast (NE Castile, Gascony, and Catalonia), this is indeed true for the mainland.

**Figure 6:**
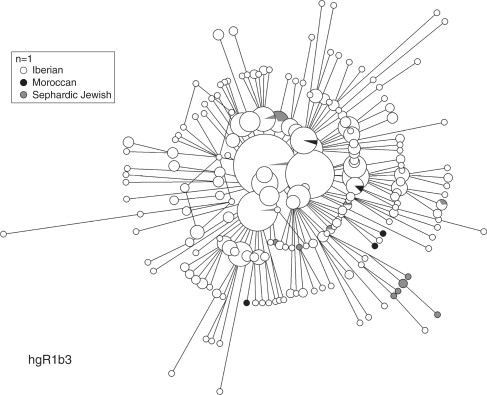
Diversity of Y-STR Haplotypes Belonging to Haplogroup R1b3 Reduced median network^53^ containing the eight-locus Y-STR (DYS19, DYS389I, DYS389II-I, DYS390, DYS391, DYS392, DYS393, DYS439) haplotypes of 767 hgR1b3 chromosomes, from Iberian populations and the Sephardic Jewish and Moroccan parental samples used in admixture analysis. Circles represent haplotypes, with area proportional to frequency and colored according to population, as shown in the key. For Iberian data, hgs R1b3b, R1b3d, R1b3f, and R1b3g have been combined into hgR1b3, because these sublineages were not distinguished in the Sephardic Jewish sample.

It is important to consider factors that might act to elevate the apparent proportions of Sephardic Jewish ancestry that we estimate, because these values are surprisingly high. Choice of parental populations in admixture analysis can have a major effect on the outcome, and among the parental populations in our analysis, the Sephardic Jewish population has a different status compared to the two others: whereas Basque and Moroccan samples are drawn from sizeable populations that have maintained their existence in situ, with a probable low level of admixture with the other parentals, the Sephardic Jewish sample is taken from a comparatively small group of self-defined individuals whose ancestors have lived in various parts of the Iberian Peninsula and were themselves probably subject to some degree of admixture with Iberians. This potential past admixture would have the effect of increasing the perceived level of Sephardic Jewish ancestry compared to the actual proportion. The presence of the typically western European lineage hgR1b3 at a frequency of 11% in the Sephardic Jewish sample might be a signal of such introgression. To examine this, we constructed a network of hgR1b3 Y-STR haplotypes in Iberian, Sephardic Jewish, and Moroccan samples (Figure 6). Twelve of the 20 Sephardic Jewish R1b3 haplotypes are shared with Iberian examples, suggesting that they will indeed affect the admixture proportions. However, eight of the 20 are unique, and five of these are peripheral in the network. They will have little impact on the admixture proportions, and they probably reflect R1b3 chromosomes of Middle Eastern origin. It therefore seems that, overall, the ancestry proportions are likely to be only slightly affected by Iberian admixture into the Sephardic Jewish sample.

An additional factor that could lead to overestimation of Sephardic Jewish ancestry proportions is the effect of other influences on the Iberian Peninsula from eastern Mediterranean populations that might have imported lineages such as G, K^∗^, and J. These influences fall into two different time periods: the Neolithic era, beginning in 10 KYA, the demographic effects of which are a matter for heated debate;^1^ and the last three millennia, the time period of Greek and Phoenician colonization.^65^ Effects in the second case are expected to be most marked in the eastern part of our sample area, but despite this, the apparent Sephardic Jewish ancestry proportions remain substantial in the west (Figure 4). The confounding effects of earlier population movement are likely to be particularly strong for Ibiza, Majorca, and Minorca, whose island natures make them more susceptible to influence by immigration and subsequent drift than inland sites. For example, history records that Ibiza, found to have a high apparent Sephardic Jewish ancestry proportion in our study, had an insignificant Jewish population compared to its neighbors^66^ yet had previously been an important Phoenician colony. Likewise, Minorca is recorded as having a substantial Jewish population,^66^ yet here, it shows no Sephardic Jewish ancestry.

Our study has focused on the Y chromosome, but can we say anything about whether admixture has been predominantly male-mediated? Some mtDNA studies^32^, ^33^ find evidence of the characteristic North African haplogroup U6 within the Iberian Peninsula. Although the overall absolute frequency of U6 is low (2.4%^33^), this signals a possible current North African ancestry proportion of 8%–9%, because U6 is not a common lineage in North Africa itself. If this figure is reliable, it is not dissimilar from the level of paternal ancestry that we find. This might suggest that initial admixture involved movement of approximately equal numbers of males and females. However, because of drift through the differential reproductive success of males and females carrying different lineages, current relative proportions are an unreliable guide to proportions of the past. Comparable mtDNA data reflecting Sephardic Jewish contributions to the various areas of Iberia are not available, but sequence data on hypervariable regions I and II in a sample of 31 Sephardic Jews from Turkey has shown that their sequences and haplogroup frequencies are similar to those of Iberian populations,^67^ suggesting that admixture might be difficult to detect. Interestingly, analysis of European genome-wide SNP data^68^ shows the western half of the Iberian Peninsula to display the highest mean heterozygosity values in the continent, an observation that might reflect its history of population admixture from very different sources.

In this study, we have demonstrated the dramatic impact of recent events on the genetic landscape of an important part of the European continent. Immigration events from the Middle East and North Africa over the last two millennia, followed by introgression driven by religious conversion and intermarriage, seem likely to have contributed a substantial proportion of the patrilineal ancestry of modern populations of Spain, Portugal, and the Balearic Islands. In studies that seek to trace the imprint of key events in the earlier prehistory of Europe, the impacts of such recent episodes of gene flow and integration must be taken into account.

## Supplemental Data

Document S1. Two Tables

## References

[1] Jobling M.A., Hurles M.E., Tyler-Smith C. (2004). Human Evolutionary Genetics: origins, peoples and disease.

[2] Torroni A., Bandelt H.J., Macaulay V., Richards M., Cruciani F., Rengo C., Martinez-Cabrera V., Villems R., Kivisild T., Metspalu E. (2001). A signal, from human mtDNA, of postglacial recolonization in Europe. Am. J. Hum. Genet..

[3] Achilli A., Rengo C., Magri C., Battaglia V., Olivieri A., Scozzari R., Cruciani F., Zeviani M., Briem E., Carelli V. (2004). The molecular dissection of mtDNA haplogroup H confirms that the Franco-Cantabrian glacial refuge was a major source for the European gene pool. Am. J. Hum. Genet..

[4] Rootsi S., Magri C., Kivisild T., Benuzzi G., Help H., Bermisheva M., Kutuev I., Barac L., Pericic M., Balanovsky O. (2004). Phylogeography of Y-chromosome haplogroup I reveals distinct domains of prehistoric gene flow in Europe. Am. J. Hum. Genet..

[5] Pereira L., Richards M., Goios A., Alonso A., Albarran C., Garcia O., Behar D.M., Golge M., Hatina J., Al-Gazali L. (2005). High-resolution mtDNA evidence for the late-glacial resettlement of Europe from an Iberian refugium. Genome Res..

[6] Richards M., Côrte-Real H., Forster P., Macaulay V., Wilkinson-Herbots H., Demaine A., Papiha S., Hedges R., Bandelt H.-J., Sykes B. (1996). Paleolithic and neolithic lineages in the European mitochondrial gene pool. Am. J. Hum. Genet..

[7] Richards M., Macaulay V., Hickey E., Vega E., Sykes B., Guida V., Rengo C., Sellitto D., Cruciani F., Kivisild T. (2000). Tracing European founder lineages in the near eastern mtDNA pool. Am. J. Hum. Genet..

[8] Simoni L., Calafell F., Pettener D., Bertranpetit J., Barbujani G. (2000). Geographic patterns of mtDNA diversity in Europe. Am. J. Hum. Genet..

[9] Rosser Z.H., Zerjal T., Hurles M.E., Adojaan M., Alavantic D., Amorim A., Amos W., Armenteros M., Arroyo E., Barbujani G. (2000). Y-chromosomal diversity within Europe is clinal and influenced primarily by geography, rather than by language. Am. J. Hum. Genet..

[10] Semino O., Passarino G., Oefner P.J., Lin A.A., Arbuzova S., Beckman L.E., De Benedictis G., Francalacci P., Kouvatsi A., Limborska S. (2000). The genetic legacy of Paleolithic Homo sapiens sapiens in extant Europeans: a Y chromosome perspective. Science.

[11] Dupanloup I., Bertorelle G., Chikhi L., Barbujani G. (2004). Estimating the impact of prehistoric admixture on the genome of Europeans. Mol. Biol. Evol..

[12] Chikhi L., Destro-Bisol G., Bertorelle G., Pascali V., Barbujani G. (1998). Clines of nuclear DNA markers suggest a largely neolithic ancestry of the European gene pool. Proc. Natl. Acad. Sci. USA.

[13] Belle E.M., Landry P.A., Barbujani G. (2006). Origins and evolution of the Europeans' genome: evidence from multiple microsatellite loci. Proc Biol Sci.

[14] Roewer L., Croucher P.J., Willuweit S., Lu T.T., Kayser M., Lessig R., de Knijff P., Jobling M.A., Tyler-Smith C., Krawczak M. (2005). Signature of recent historical events in the European Y-chromosomal STR haplotype distribution. Hum. Genet..

[15] Carr R. (2000). Spain: a history.

[16] Fletcher R. (1992). Moorish Spain.

[17] Collins R. (1989). The Arab Conquest of Spain.

[18] Boone J.L., Benco N.L. (1999). Islamic settlement in North Africa and the Iberian Peninsula. Annu. Rev. Anthropol..

[19] Glick T.F. (1979). Islamic and Christian Spain in the Early Middle Ages.

[20] Guichard P. (1976). Al-Andalus: Estructura antropologica de una sociedade islamica en Occidente.

[21] Bulliet R.W. (1979). Conversion to Islam in the medieval period: an essay in quantitative history.

[22] Anon. (1147) De Expugnatione Lyxbonensi, codex 470, folios 125–146. Corpus Christi College Library, University of Cambridge.

[23] Harvey L.P. (2005). Muslims in Spain: 1500 to 1614.

[24] Gerber J.S. (1992). The Jews of Spain: a History of the Sephardic Experience.

[25] Mackay A., Kedourie E. (1992). The Jews in Spain during the Middle Ages. Spain and the Jews: the Sephardi Experience, 1492 and after.

[26] Caro Baroja J. (1978). Los judios en la España moderna y contemporanea, Volume 1.

[27] Reyment R.A. (1983). Moors and Christians: an example of multivariate analysis applied to human blood-groups. Ann. Hum. Biol..

[28] Bertranpetit J., Cavalli-Sforza L.L. (1991). A genetic reconstruction of the history of the population of the Iberian Peninsula. Ann. Hum. Genet..

[29] Calafell F., Bertranpetit J. (1994). Principal component analysis of gene frequencies and the origin of Basques. Am. J. Phys. Anthropol..

[30] Fernandez-Santander A., Kandil M., Luna F., Esteban E., Gimenez F., Zaoui D., Moral P. (1999). Genetic relationships between southeastern Spain and Morocco: New data on ABO, RH, MNSs, and DUFFY polymorphisms. Am. J. Hum. Biol..

[31] Comas D., Calafell F., Benchemsi N., Helal A., Lefranc G., Stoneking M., Batzer M.A., Bertranpetit J., Sajantila A. (2000). Alu insertion polymorphisms in NW Africa and the Iberian Peninsula: evidence for a strong genetic boundary through the Gibraltar Straits. Hum. Genet..

[32] Côrte-Real H.B.S.M., Macaulay V.A., Richards M.B., Hariti G., Issad M.S., Cambon-Thomsen A., Papiha S., Bertranpetit J., Sykes B.C. (1996). Genetic diversity in the Iberian Peninsula determined from mitochondrial sequence analysis. Ann. Hum. Genet..

[33] Pereira L., Cunha C., Alves C., Amorim A. (2005). African female heritage in Iberia: a reassessment of mtDNA lineage distribution in present times. Hum. Biol..

[34] Bosch E., Calafell F., Comas D., Oefner P.J., Underhill P.A., Bertranpetit J. (2001). High-resolution analysis of human Y-chromosome variation shows a sharp discontinuity and limited gene flow between Northwestern Africa and the Iberian Peninsula. Am. J. Hum. Genet..

[35] Scozzari R., Cruciani F., Pangrazio A., Santolamazza P., Vona G., Moral P., Latini V., Varesi L., Memmi M.M., Romano V. (2001). Human Y-chromosome variation in the western Mediterranean area: implications for the peopling of the region. Hum. Immunol..

[36] Brion M., Salas A., Gonzalez-Neira A., Lareu M.V., Carracedo A. (2003). Insights into Iberian population origins through the construction of highly informative Y-chromosome haplotypes using biallelic markers, STRs, and the MSY1 minisatellite. Am. J. Phys. Anthropol..

[37] Cruciani F., La Fratta R., Santolamazza P., Sellitto D., Pascone R., Moral P., Watson E., Guida V., Colomb E.B., Zaharova B. (2004). Phylogeographic analysis of haplogroup E3b (E-M215) y chromosomes reveals multiple migratory events within and out of Africa. Am. J. Hum. Genet..

[38] Flores C., Maca-Meyer N., Gonzalez A.M., Oefner P.J., Shen P., Perez J.A., Rojas A., Larruga J.M., Underhill P.A. (2004). Reduced genetic structure of the Iberian Peninsula revealed by Y-chromosome analysis: implications for population demography. Eur. J. Hum. Genet..

[39] Cruciani F., La Fratta R., Trombetta B., Santolamazza P., Sellitto D., Colomb E.B., Dugoujon J.M., Crivellaro F., Benincasa T., Pascone R. (2007). Tracing past human male movements in northern/eastern Africa and western Eurasia: new clues from Y-chromosomal haplogroups E-M78 and J-M12. Mol. Biol. Evol..

[40] Jobling M.A., Tyler-Smith C. (2003). The human Y chromosome: an evolutionary marker comes of age. Nat. Rev. Genet..

[41] Gonçalves R., Freitas A., Branco M., Rosa A., Fernandes A.T., Zhivotovsky L.A., Underhill P.A., Kivisild T., Brehm A. (2005). Y-chromosome lineages from Portugal, Madeira and Açores record elements of Sephardim and Berber ancestry. Ann. Hum. Genet..

[42] Beleza S., Gusmao L., Lopes A., Alves C., Gomes I., Giouzeli M., Calafell F., Carracedo A., Amorim A. (2006). Micro-phylogeographic and demographic history of Portuguese male lineages. Ann. Hum. Genet..

[43] Bosch E., Calafell F., González-Neira A., Flaiz C., Mateu E., Scheil H.-G., Huckenbeck W., Efremovska L., Mikerezi I., Xirotiris N. (2006). Male and female lineages in the Balkans show a homogeneous landscape over linguistic barriers, except for the isolated Aromuns. Ann. Hum. Genet..

[44] Adams S.M., King T.E., Bosch E., Jobling M.A. (2006). The case of the unreliable SNP: Recurrent back-mutation of Y-chromosomal marker P25 through gene conversion. Forensic Sci. Int..

[45] Y Chromosome Consortium (2002). A nomenclature system for the tree of human Y-chromosomal binary haplogroups. Genome Res..

[46] Bosch E., Lee A.C., Calafell F., Arroyo E., Henneman P., de Knijff P., Jobling M.A. (2002). High resolution Y chromosome typing: 19 STRs amplified in three multiplex reactions. Forensic Sci. Int..

[47] Arredi B., Poloni E.S., Paracchini S., Zerjal T., Fathallah D.M., Makrelouf M., Pascali V.L., Novelletto A., Tyler-Smith C. (2004). A predominantly neolithic origin for Y-chromosomal DNA variation in North Africa. Am. J. Hum. Genet..

[48] Bosch E., Calafell F., Santos F.R., Pérez-Lezaun A., Comas D., Benchemsi N., Tyler-Smith C., Bertranpetit J. (1999). Variation in short tandem repeats is deeply structured by genetic background on the human Y chromosome. Am. J. Hum. Genet..

[49] Schlecht J., Kaplan M.E., Barnard K., Karafet T., Hammer M.F., Merchant N.C. (2008). Machine-learning approaches for classifying haplogroup from Y chromosome STR data. PLoS Comput Biol.

[50] Robino C., Crobu F., Di Gaetano C., Bekada A., Benhamamouch S., Cerutti N., Piazza A., Inturri S., Torre C. (2008). Analysis of Y-chromosomal SNP haplogroups and STR haplotypes in an Algerian population sample. Int. J. Legal Med..

[51] Behar D., Skorecki K., Skolnik F. (2006). Genetic ancestry, Jewish. Encyclopaedia Judaica.

[52] Schneider S., Roessli D., Excoffier L. (2000). Arlequin ver. 2.0: A Software for Population Genetics Data Analysis.

[53] Bandelt H.-J., Forster P., Röhl A. (1999). Median-joining networks for inferring intraspecific phylogenies. Mol. Biol. Evol..

[54] Qamar R., Ayub Q., Mohyuddin A., Helgason A., Mazhar K., Mansoor A., Zerjal T., Tyler-Smith C., Mehdi S.Q. (2002). Y-chromosomal DNA variation in Pakistan. Am. J. Hum. Genet..

[55] Dupanloup I., Bertorelle G. (2001). Inferring admixture proportions from molecular data: extension to any number of parental populations. Mol. Biol. Evol..

[56] Bertorelle G., Excoffier L. (1998). Inferring admixture proportions from molecular data. Mol. Biol. Evol..

[57] Helgason A., Sigurdardóttir S., Nicholson J., Sykes B., Hill E.W., Bradley D.G., Bosnes V., Gulcher J.R., Ward R., Stefánsson K. (2000). Estimating Scandinavian and Gaelic ancestry in the male settlers of Iceland. Am. J. Hum. Genet..

[58] Underhill P.A., Jin L., Lin A.A., Mehdi S.Q., Jenkins T., Vollrath D., Davis R.W., Cavalli-Sforza L.L., Oefner P.J. (1997). Detection of numerous Y chromosome biallelic polymorphisms by denaturing high-performance liquid chromatography. Genome Res..

[59] Underhill P.A., Shen P., Lin A.A., Jin L., Passarino G., Yang W.H., Kauffman E., Bonné-Tamir B., Bertranpetit J., Francalacci P. (2000). Y chromosome sequence variation and the history of human populations. Nat. Genet..

[60] Weale M.E., Weiss D.A., Jager R.F., Bradman N., Thomas M.G. (2002). Y chromosome evidence for Anglo-Saxon mass migration. Mol. Biol. Evol..

[61] Bowden G.R., Balaresque P., King T.E., Hansen Z., Lee A.C., Pergl-Wilson G., Hurley E., Roberts S.J., Waite P., Jesch J. (2008). Excavating past population structures by surname-based sampling: the genetic legacy of the Vikings in northwest England. Mol. Biol. Evol..

[62] Escacena J.L., Arnaiz-Villena A. (2000). Applications of evolutive archeology: migrations from Africa to Iberia in the recent prehistory. Prehistoric Iberia Genetics.

[63] Anderung C., Bouwman A., Persson P., Carretero J.M., Ortega A.I., Elburg R., Smith C., Arsuaga J.L., Ellegren H., Götherstrom A. (2005). Prehistoric contacts over the Straits of Gibraltar indicated by genetic analysis of Iberian Bronze Age cattle. Proc. Natl. Acad. Sci. USA.

[64] Brion M., Sobrino B., Blanco-Verea A., Lareu M.V., Carracedo A. (2004). Hierarchical analysis of 30 Y-chromosome SNPs in European populations. Int. J. Legal Med..

[65] Zalloua P.A., Platt D.E., El Sibai M., Khalife J., Makhoul N., Haber M., Xue Y., Izaabel H., Bosch E., Adams S.M. (2008). Identifying genetic traces of historical expansions: Phoenician footprints in the Mediterranean. Am. J. Hum. Genet..

[66] Foss A. (1975). Ibiza and Minorca.

[67] Picornell A., Gimenez P., Castro J.A., Ramon M.M. (2006). Mitochondrial DNA sequence variation in Jewish populations. Int. J. Legal Med..

[68] Lao O., Lu T.T., Nothnagel M., Junge O., Freitag-Wolf S., Caliebe A., Balascakova M., Bertranpetit J., Bindoff L.A., Comas D. (2008). Correlation between genetic and geographic structure in Europe. Curr. Biol..

