# Supplementary material for: The Genetic Legacy of Religious Diversity and Intolerance: Paternal Lineages of Christians, Jews, and Muslims in the Iberian Peninsula
Source: Am J Hum Genet. 2008 Dec 5;83(6):725–36. doi: 10.1016/j.ajhg.2008.11.007 (PMC2668061; doi:10.1016/j.ajhg.2008.11.007)
Supplement: Document S1. Two Tables [file mmc1.pdf]

AJHG, Volume *83*

Supplemental Data

The Genetic Legacy of Religious Diversity and Intolerance: Paternal Lineages of Christians, Jews, and Muslims in the Iberian Peninsula

Susan M. Adams, Elena Bosch, Patricia L. Balaresque, Stéphane J. Ballereau, Andrew C. Lee, Eduardo Arroyo, Ana M. López-Parra, Mercedes Aler, Marina S. Gisbert Grifo, Maria Brion, Angel Carracedo, João Lavinha, Begoña Martínez-Jarreta, Lluis Quintana-Murci, Antònia Picornell, Misericordia Ramon, Karl Skorecki, Doron M. Behar, Francesc Calafell, and Mark A. Jobling

Table S1. Haplogroups[a] and Y-STR Haplotypes of Iberian Peninsula and Sephardic Jewish Samples[b]

Name	Pop.	Hg	DYS19	DYS388	DYS389I	389 II-Ii	DYS390	DYS391	DYS392	DYS393	DYS434	DYS435	DYS436	DYS437	DYS438	DYS439	DYS460	DYS461	DYS462	DYS385
**Aragon**																				
IP685	ARA	E3b*	15	12	13	17	22	9	11	14	11	11	12	14	10	11	11	13	12	14,14
IP314	ARA	E3b2	13	12	14	16	24	9	11	13	11	11	12	14	10	10	11	12	12	13,14
IP292	ARA	I	14	14	12	16	22	10	11	13	11	11	12	16	10	11	10	12	12	13,14
IP534	ARA	I	17	13	14	15	25	9	11	13	11	11	12	15	10	12	10	12	12	12,12
IP575	ARA	I	15	13	14	17	23	10	12	14	11	11	12	15	10	12	11	12	12	16,16
IP577	ARA	I	14	14	12	17	22	10	11	13	11	11	12	15	10	11	10	12	12	14,14
IP580	ARA	I	17	13	13	15	24	10	11	13	11	11	12	15	10	13	10	11	13	12,12
IP586	ARA	I	17	13	14	15	25	9	11	13	11	11	12	15	10	12	10	12	12	12,13
IP537	ARA	J2	15	15	12	18	24	10	11	12	11	11	12	16	9	13	11	10	10	13,17
IP571	ARA	J2	14	15	13	16	23	11	11	13	11	11	12	15	9	11	11	12	11	12,17
IP574	ARA	J2	14	16	13	15	23	11	11	12	11	10	12	15	9	11	10	12	11	12,17
IP584	ARA	J2	15	15	12	17	23	10	11	12	11	11	12	16	9	12	11	10	11	14,17
IP521	ARA	K(XP)	13	12	14	17	23	10	13	14	11	11	12	15	9	12	11	11	12	16,17
IP572	ARA	K(xP)	16	12	12	15	23	10	14	13	11	11	12	14	9	9	10	11	12	14,15
IP579	ARA	R1a1	15	12	13	18	25	11	11	13	11	11	12	14	11	10	11	11	11	11,14
IP369	ARA	R1b3*	14	12	13	16	24	11	13	13	11	11	12	14	12	12	10	12	11	11,14
IP533	ARA	R1b3*	12	12	13	17	24	11	13	14	11	11	12	15	12	12	10	12	11	12,14
IP573	ARA	R1b3*	14	12	14	16	24	11	13	13	11	11	12	15	12	11	11	12	11	11,14
IP576	ARA	R1b3*	14	12	14	17	24	10	13	13	11	11	12	15	12	12	11	12	12	10,13
IP581	ARA	R1b3*	14	12	14	16	23	11	13	13	11	11	12	15	12	11	10	12	11	11,14
IP582	ARA	R1b3*	14	12	13	16	24	11	13	13	11	11	12	15	12	13	11	12	11	11,14
IP583	ARA	R1b3*	14	12	14	16	23	10	13	13	11	11	12	15	12	13	11	12	11	11,11
IP585	ARA	R1b3*	14	12	13	16,17	24	11	13	13	11	11	12	15	12	10,12	11	12	11	12,15

Name	Pop.	Hg	DYS19	DYS388	DYS389I	389 II-Ii	DYS390	DYS391	DYS392	DYS393	DYS434	DYS435	DYS436	DYS437	DYS438	DYS439	DYS460	DYS461	DYS462	DYS385
IP599	ARA	R1b3*	14	12	13	16	24	11	13	14	11	11	12	15	12	11	11	12	11	11,14
IP789	ARA	R1b3*	14	12	12	16	24	11	13	13	11	11	12	15	11	11	11	12	11	11,14
IP808	ARA	R1b3*	15	12	13	16	23	10	13	13	11	11	12	14	12	12	11	13	11	10,15
IP812	ARA	R1b3*	14	12	13	16	23	11	13	13	11	11	12	15	12	12	11	12	11	11,13
IP864	ARA	R1b3*	14	12	13	16	23	10	13	13	11	11	12	14	12	12	11	12	11	11,14
IP359	ARA	R1b3d	14	12	13	16	24	10	13	13	11	11	12	14	12	13	10	12	11	11,14
IP535	ARA	R1b3f	15	12	13	16	23	11	13	13	11	11	12	15	12	11	10	13	11	11,14
IP536	ARA	R1b3f	14	12	13	15	23	11	13	13	11	11	12	15	12	12	11	12	11	14,14
IP578	ARA	R1b3f	14	12	13	17	24	11	13	13	11	11	12	15	12	11	11	12	11	11,15
IP730	ARA	R1b3f	14	12	13	17	24	11	13	14	11	11	12	15	12	12	11	13	11	11,14
IP835	ARA	R1b3f	14	12	13	16	23	11	13	13	11	11	12	15	12	12	11	12	11	11,14
**Asturias**																				
IP1008	AST	E3b*	13	12	12	18	24	10	10	13	11	11	12	14	10	12	10	12	11	16,16
IP1015	AST	E3b*	13	12	14	17	24	10	11	12	11	11	12	14	11	12	10	13	12	16,19
IP1002	AST	E3b1	13	12	13	17	25	10	11	14	11	11	12	14	10	11	11	11	13	16,17
IP1019	AST	G	15	13	12	17	22	10	11	14	11	11	12	15	10	11	10	11	12	14,15
IP716	AST	I	17	13	13	15	23	10	11	13	11	11	12	15	10	11	10	11	12	12,12
IP995	AST	I	15	14	12	16	22	10	11	13	11	11	12	16	10	11	11	12	12	13,15
IP992	AST	J2	14	15	13	17	23	10	11	12	12	11	12	15	9	11	9	13	11	13,17
IP997	AST	J2	15	15	12	16	24	10	11	13	11	11	12	16	9	11	11	10	11	13,17
IP1020	AST	J2	14	15	12	16	23	11	11	13	11	11	12	15	10	12	10	12	11	14,16
IP1023	AST	K(XP)	15	12	12	15	24	10	14	13	11	11	12	14	9	11	10	11	13	13,15
IP991	AST	R1b3*	14	12	13	16	24	10	13	12	11	11	12	15	12	11	11	12	11	12,14
IP303	AST	R1b3*	15	12	13	16	23	11	13	13	11	11	12	15	12	12	11	13	11	11,13
IP680	AST	R1b3*	14	12	13	16	24	12	13	13	11	11	12	15	12	12	11	12	11	11,14
IP989	AST	R1b3*	14	12	13	16	24	11	13	13	11	11	12	15	12	11	10	13	11	11,15
IP993	AST	R1b3*	14	12	13	16	24	11	13	13	11	11	12	15	12	12	10	12	11	11,14
IP999	AST	R1b3*	14	12	13	16	24	10	13	13	11	11	12	15	12	12	10	12	11	11,14
IP1011	AST	R1b3*	14	12	13	16	24	10	13	13	11	11	12	14	12	12	11	12	11	11,14
IP1016	AST	R1b3*	14	12	13	16	24	10	13	13	11	11	12	15	12	13	11	12	11	11,15
IP1018	AST	R1b3*	14	12	14	16	22	10	13	13	11	11	12	14	12	12	13	12	11	11,14
IP1021	AST	R1b3*	14	12	12	16	23	11	14	13	11	11	12	15	12	12	11	13	11	11,14
**Basque Country**																				
IP1	BAS	E3b2	13	12	14	16	24	9	11	13	11	11	12	14	10	10	11	12	12	13,14
IP11	BAS	I	15	13	13	16	25	10	11	13	11	11	12	15	10	11	11	11	12	12,16
IP20	BAS	I	13	14	12	16	22	10	11	13	12	11	12	16	10	11	11	12	12	13,16
IP25	BAS	I	17	13	15	15	23	10	11	13	11	11	12	15	10	12	10	11	12	12,12
IP36	BAS	I	17	13	13	15	24	9	11	13	11	11	12	14	10	13	10	12	12	12,12
IP884	BAS	I	16	13	13	15	23	10	11	13	11	11	12	15	10	11	10	11	12	12,12
IP902	BAS	I	17	13	13	15	23	10	11	13	11	11	12	14	10	13	10	11	13	12,12
IP904	BAS	I	17	13	13	15	23	11	11	13	11	11	12	14	10	13	10	11	13	12,13

Name	Pop.	Hg	DYS19	DYS388	DYS389I	389 II-Ii	DYS390	DYS391	DYS392	DYS393	DYS434	DYS435	DYS436	DYS437	DYS438	DYS439	DYS460	DYS461	DYS462	DYS385
IP935	BAS	I	17	13	14	15	23	10	11	13	11	11	12	15	10	12	10	11	12	12,12
IP936	BAS	I	17	13	12	15	23	10	11	13	11	11	12	15	10	11	10	11	12	12,12
IP905	BAS	J(xJ2)	14	13	14	16	23	10	11	12	11	11	11	14	10	11	11	13	13	12,18
IP889	BAS	J2	14	15	12	17	23	10	12	11	11	11	12	14	9	11	10	11	11	13,21
IP908	BAS	J2	15	17	12	17	23	10	11	12	11	12	12	15	9	11	11	13	11	12,16
IP910	BAS	J2	16	16	13	16	23	9	11	12	11	11	12	14	9	12	10	13	10	13,15
IP906	BAS	Q(xQ3)	13	12	14	18	24	10	14	13	11	11	12	13	11	12	10	12	12	15,16
IP3	BAS	R1b3*	14	12	13	16	24	11	13	13	11	11	12	15	12	11	11	12	11	12,14
IP4	BAS	R1b3*	14	12	12	16	24	11	13	13	11	11	12	15	12	12	11	12	11	11,14
IP6	BAS	R1b3*	14	12	12	16	24	11	13	13	11	11	12	15	12	11	11	12	11	11,14
IP7	BAS	R1b3*	14	12	13	16	24	10	13	13	11	11	12	15	12	11	12	12	11	11,13
IP8	BAS	R1b3*	14	12	13	16	24	11	13	13	11	11	12	15	12	12	11	12	11	11,14
IP10	BAS	R1b3*	14	12	14	16	23	11	13	14	11	11	12	14	12	13	11	12	11	11,14
IP12	BAS	R1b3*	14	12	13	16	25	10	13	12	11	11	12	15	12	13	11	12	11	11,13
IP15	BAS	R1b3*	14	12	14	17	24	10	13	13	11	11	12	14	12	12	10	12	11	11,14
IP16	BAS	R1b3*	14	12	13	16	24	10	13	13	11	11	12	15	12	12	11	12	11	11,14
IP18	BAS	R1b3*	15	12	13	17	23	11	13	13	11	11	12	15	12	12	10	12	11	11,14
IP19	BAS	R1b3*	14	12	14	16	24	11	13	13	11	11	12	15	12	11	11	12	11	11,14
IP21	BAS	R1b3*	14	12	13	15	23	11	13	15	11	11	12	15	12	11	11	12	11	11,14
IP22	BAS	R1b3*	14	12	13	16	24	11	13	13	11	11	12	15	12	12	11	12	11	11,14
IP23	BAS	R1b3*	14	12	13	16	24	11	13	13	11	11	12	15	12	12	11	12	11	12,14
IP24	BAS	R1b3*	14	12	14	16	23	11	13	13	11	11	12	15	12	13	10	13	11	11,11
IP26	BAS	R1b3*	14	12	13	16	24	11	13	13	11	11	12	15	12	12	11	12	11	10,14
IP27	BAS	R1b3*	14	12	12	16	24	11	13	13	11	11	12	15	12	12	11	12	11	11,14
IP29	BAS	R1b3*	14	12	14	16	24	11	13	13	11	11	12	15	12	12	11	12	11	11,13
IP31	BAS	R1b3*	13	12	13	16	25	10	13	12	11	11	12	15	12	14	11	12	11	11,13
IP32	BAS	R1b3*	14	12	13	16	24	11	13	13	11	11	12	15	12	12	11	12	11	12,14
IP33	BAS	R1b3*	14	12	11	16	24	10	13	13	11	11	12	15	12	12	11	12	11	11,14
IP34	BAS	R1b3*	14	12	13	16	23	10	13	13	11	11	12	15	12	13	11	12	11	11,11
IP37	BAS	R1b3*	14	12	13	17	24	11	13	13	11	11	12	15	12	12	12	12	11	11,14
IP38	BAS	R1b3*	14	12	12	16	24	11	13	13	11	11	12	15	12	12	11	12	11	11,14
IP39	BAS	R1b3*	14	12	13	16	24	12	13	13	11	11	12	15	12	12	11	12	11	11,14
IP40	BAS	R1b3*	14	12	13	15	24	10	13	13	11	11	12	15	12	11	11	12	11	11,15
IP44	BAS	R1b3*	14	12	13	16	24	11	13	13	11	11	12	15	12	12	11	12	11	11,14
IP45	BAS	R1b3*	14	12	14	16	24	11	13	13	11	11	12	15	12	11	11	11	12	11,15
IP46	BAS	R1b3*	14	12	14	16	24	10	13	13	11	11	12	15	12	11	11	12	11	11,14
IP48	BAS	R1b3*	14	12	14	16	23	10	13	13	11	11	12	15	13	12	11	12	11	11,11
IP49	BAS	R1b3*	14	12	13	16	24	11	13	13	11	11	12	14	12	11	11	12	11	11,14
IP527	BAS	R1b3*	14	12	14	16	24	11	13	13	11	11	12	15	12	11	11	12	11	11,14
IP531	BAS	R1b3*	14	12	13	16	24	11	13	13	11	11	12	15	12	12	11	12	11	11,14
IP532	BAS	R1b3*	14	12	13	16	25	10	13	12	11	11	12	15	12	13	10	13	11	11,13
IP877	BAS	R1b3*	14	12	12	16	23	11	13	14	11	11	12	14	12	13	11	12	11	12,14
IP878	BAS	R1b3*	14	12	14	16	24	10	13	13	11	11	12	14	12	11	11	12	11	11,13

Name	Pop.	Hg	DYS19	DYS388	DYS389I	389 II-Ii	DYS390	DYS391	DYS392	DYS393	DYS434	DYS435	DYS436	DYS437	DYS438	DYS439	DYS460	DYS461	DYS462	DYS385
IP880	BAS	R1b3*	14	12	14	16	24	11	13	13	11	11	12	15	12	13	10	12	11	11,14
IP881	BAS	R1b3*	14	12	14	16	24	11	13	13	11	11	12	14	12	11	10	12	11	11,14
IP882	BAS	R1b3*	14	12	13	16	24	11	13	13	11	11	12	15	12	13	11	12	11	11,14
IP883	BAS	R1b3*	14	12	13	16	23	11	13	13	11	11	12	15	12	12	10	13	11	11,14
IP885	BAS	R1b3*	14	12	13	17	24	11	13	13	11	11	12	15	12	11	11	12	11	11,14
IP887	BAS	R1b3*	14	12	13	16	24	11	13	13	11	11	12	15	12	13	11	13	11	11,15
IP890	BAS	R1b3*	14	12	13	16	25	11	13	13	11	11	12	15	12	11	11	11	11	11,12
IP891	BAS	R1b3*	14	12	13	17	24	11	13	13	11	11	12	15	12	11	12	12	11	11,14
IP892	BAS	R1b3*	14	12	15	16	24	11	13	12	11	12	12	15	12	11	11	12	11	11,14
IP893	BAS	R1b3*	14	12	14	15	23	11	13	13	11	12	12	14	12	12	10	12	11	12,14
IP894	BAS	R1b3*	14	12	14	16	23	11	13	13	11	11	12	14	12	12	10	12	11	11,11
IP897	BAS	R1b3*	14	12	12	16	23	11	13	14	11	11	12	14	12	13	11	12	11	11,14
IP898	BAS	R1b3*	14	12	13	16	24	10	12	13	11	11	12	15	12	12	10	12	11	11,14
IP899	BAS	R1b3*	14	12	14	16	23	10	13	13	11	11	12	15	12	13	11	12	11	11,11
IP900	BAS	R1b3*	14	12	13	16	24	11	13	13	11	11	12	15	12	11	11	12	11	12,14
IP901	BAS	R1b3*	14	12	13	16	25	10	13	13	11	11	12	15	12	12	10	12	11	11,14
IP903	BAS	R1b3*	14	12	14	17	24	11	13	13	11	11	12	14	12	12	10	12	11	12,14
IP907	BAS	R1b3*	14	12	13	15	24	11	14	12	11	11	12	15	12	12	11	12	11	11,14
IP909	BAS	R1b3*	14	12	14	15	24	11	13	14	11	12	12	14	12	12	10	12	11	12,14
IP911	BAS	R1b3*	14	12	14	17	24	11	13	13	11	11	12	15	12	12	12	12	11	11,14
IP925	BAS	R1b3*	14	12	14	16	24	11	13	13	11	11	12	15	12	11	11	12	11	11,14
IP927	BAS	R1b3*	14	12	13	16	24	10	13	13	11	11	12	15	12	12	11	12	11	12,14
IP928	BAS	R1b3*	14	12	14	17	24	11	13	13	11	11	12	14	12	13	10	12	11	11,14
IP929	BAS	R1b3*	14	12	14	17	23	11	13	13	11	11	12	14	12	12	10	12	11	11,14
IP931	BAS	R1b3*	14	12	13	16	25	10	13	12	11	11	12	15	12	13	10	12	11	11,13
IP932	BAS	R1b3*	14	12	13	16	24	11	13	13	11	11	12	15	12	12	11	12	11	11,14
IP933	BAS	R1b3*	14	12	14	18	24	10	13	13	11	11	12	15	12	12	10	12	11	11,14
IP934	BAS	R1b3*	14	12	14	16	24	10	13	13	11	11	12	14	12	12	10	12	11	11,14
IP937	BAS	R1b3*	14	12	13	16	24	11	13	13	11	11	12	15	12	11	11	12	11	11,14
IP938	BAS	R1b3*	14	12	13	16	25	10	13	12	11	11	12	15	12	13	11	12	11	11,13
IP939	BAS	R1b3*	14	12	14	16	24	11	13	13	11	11	12	14	12	11	10	12	11	11,14
IP940	BAS	R1b3*	14	12	13	16	24	11	13	13	11	11	12	15	12	12	10	12	12	11,14
IP941	BAS	R1b3*	14	12	14	16	24	11	13	13	11	11	12	15	12	11	11	12	11	11,14
IP942	BAS	R1b3*	12	12	13	17	24	11	13	13	11	11	12	15	12	11	11	12	11	11,14
IP945	BAS	R1b3*	14	12	14	16	23	11	13	14	11	11	12	14	12	14	11	12	11	11,14
IP946	BAS	R1b3*	14	12	14	18	24	10	13	13	11	11	12	14	12	11	10	12	11	11,14
IP947	BAS	R1b3*	14	12	14	17	24	11	13	13	11	11	12	14	12	12	10	13	11	11,15
IP47	BAS	R1b3b	15	12	14	16	23	10	13	13	11	11	12	15	12	13	11	12	11	11,11
IP50	BAS	R1b3b	15	12	14	16	23	10	13	13	11	11	12	15	12	13	11	12	11	11,11
IP2	BAS	R1b3d	14	12	14	16	24	11	13	13	11	11	12	14	12	11	10	13	11	11,14
IP9	BAS	R1b3d	14	12	13	16	24	11	13	13	11	11	12	14	12	12	10	12	11	11,14
IP13	BAS	R1b3d	14	12	13	16	24	11	13	14	11	11	12	14	12	13	10	12	11	11,14
IP17	BAS	R1b3d	14	12	13	16	23	10	13	14	12	11	12	14	12	12	10	12	11	11,14

Name	Pop.	Hg	DYS19	DYS388	DYS389I	389 II-Ii	DYS390	DYS391	DYS392	DYS393	DYS434	DYS435	DYS436	DYS437	DYS438	DYS439	DYS460	DYS461	DYS462	DYS385
IP28	BAS	R1b3d	14	12	13	16	24	11	13	13	11	11	12	15	12	12	10	12	11	11,14
IP30	BAS	R1b3d	14	12	13	16	24	11	13	13	11	11	12	14	12	12	10	12	11	11,14
IP35	BAS	R1b3d	14	12	13	16	24	10	13	13	11	11	12	14	12	12	10	12	11	11,14
IP42	BAS	R1b3d	14	12	14	16	24	11	13	13	11	11	12	14	12	11	10	12	11	11,14
IP526	BAS	R1b3d	14	12	13	17	24	9	13	13	11	11	12	14	12	12	10	12	11	11,14
IP879	BAS	R1b3d	14	12	13	16	24	10	13	13	11	11	12	14	12	12	10	12	11	11,14
IP886	BAS	R1b3d	14	12	13	16	24	10	13	13	11	11	12	14	12	12	10	12	11	11,14
IP888	BAS	R1b3d	14	12	13	16	24	10	13	13	11	11	12	14	12	12	10	12	11	11,14
IP895	BAS	R1b3d	14	12	14	16	24	10	13	13	11	11	10,12	14	13	12	11	12	11	11,14
IP896	BAS	R1b3d	13	12	13	17	24	10	13	13	11	11	12	14	11	12	10	12	11	11,14
IP944	BAS	R1b3d	14	12	13	16	24	11	13	14	11	11	12	14	12	12	10	12	12	11,14
IP5	BAS	R1b3f	14	12	14	16	24	10	13	13	11	11	12	15	12	13	10	12	11	11,13
IP14	BAS	R1b3f	14	12	13	16	24	10	13	13	11	11	12	15	12	12	11	12	11	11,14
IP41	BAS	R1b3f	14	12	14	16	24	10	13	13	11	11	12	15	12	13	10	12	11	12,13
IP43	BAS	R1b3f	14	12	14	16	24	11	13	13	11	11	12	15	12	13	10	12	11	11,13
IP82	BAS	R1b3f	14	12	13	16	24	10	13	13	11	11	12	15	12	12	11	12	11	11,14
IP83	BAS	R1b3f	14	12	13	16	24	10	13	13	11	11	12	15	12	12	10	12	11	11,14
IP529	BAS	R1b3f	14	12	14	16	24	11	13	13	11	11	12	15	12	12	10	12	11	11,14
IP530	BAS	R1b3f	14	12	14	16	24	11	13	13	11	11	12	15	12	13	10	12	11	11,14
IP926	BAS	R1b3f	14	12	13	16	24	10	13	13	11	11	12	15	12	12	11	12	11	11,14
IP930	BAS	R1b3f	14	12	13	16	24	12	13	13	11	11	12	15	12	11	11	12	12	11,14
IP943	BAS	R1b3f	14	12	14	16	24	11	13	13	11	11	12	15	12	13	10	12	11	11,14
**Catalonia**																				
IP73	CAT	E3b1	13	12	13	17	24	10	11	13	11	11	12	14	10	12	11	12	12	16,18
IP53	CAT	E3b2	13	12	14	16	24	9	11	13	11	11	12	14	10	10	11	13	12	13,15
IP548	CAT	E3b3	15	12	13	18	23	10	11	14	11	11	12	14	11	12	10	12	11	15,17
IP59	CAT	G	15	13	12	17	22	10	11	14	11	11	12	16	10	12	10	11	12	14,15
IP67	CAT	G	15	12	12	18	22	10	11	13	11	11	12	16	10	12	11	10	12	12,14
IP525	CAT	G	15	13	12	17	22	11	11	15	11	11	12	16	10	11	10	11	12	15,15
IP555	CAT	G	15	12	12	16	21	10	11	13	12	11	12	16	10	11	9	11	12	12,14
IP567	CAT	G	15	13	12	16	22	10	12	13	11	11	12	16	10	11	10	11	12	14,14
IP546	CAT	I	15	13	13	16	26	10	11	13	11	11	12	15	10	11	10	11	12	12,16
IP557	CAT	I	17	13	14	15	25	9	11	13	11	11	12	15	10	12	10	12	12	12,12
IP55	CAT	J2	14	15	13	18	25	10	11	12	11	11	12	14	9	11	12	11	10	14,18
IP64	CAT	J2	15	15	13	18	23	9	11	13	11	11	12	15	9	12	10	13	11	12,13
IP543	CAT	J2	14	15	14	17	23	11	11	12	10	11	12	15	9	11	9	12	11	12,14
IP570	CAT	J2	14	16	13	16	23	10	11	12	11	11	12	14	10	11	11	10	11	13,17
IP588	CAT	J2	16	16	13	16	23	9	11	12	11	11	12	14	9	11	10	13	10	13,16
IP52	CAT	R1b3*	14	12	13	16	24	10	13	13	12	11	12	15	12	13	11	12	11	11,14
IP54	CAT	R1b3*	15	12	13	16	24	10	13	13	11	11	12	15	13	13	10	12	11	11,15
IP56	CAT	R1b3*	14	12	13	16	24	11	13	13	11	11	12	15	12	11	11	12	11	11,14
IP57	CAT	R1b3*	14	12	14	16	24	11	13	13	11	11	12	15	11	11	10	13	11	11,14

Name	Pop.	Hg	DYS19	DYS388	DYS389I	389 II-Ii	DYS390	DYS391	DYS392	DYS393	DYS434	DYS435	DYS436	DYS437	DYS438	DYS439	DYS460	DYS461	DYS462	DYS385
IP58	CAT	R1b3*	14	12	13	16	24	11	13	13	11	11	12	15	12	12	11	12	11	11,14
IP60	CAT	R1b3*	14	13	13	16	24	11	13	13	11	11	12	16	12	12	11	12	11	12,14
IP61	CAT	R1b3*	14	12	13	16	24	11	13	13	11	11	12	15	13	13	11	12	11	11,13
IP62	CAT	R1b3*	14	12	13	16	24	11	13	13	11	11	12	15	12	12	11	12	11	11,15
IP63	CAT	R1b3*	15	12	13	16	24	10	13	13	11	11	12	15	12	11	10	12	11	11,15
IP65	CAT	R1b3*	14	12	13	16	24	12	14	14	11	11	12	15	12	12	11	12	11	12,14
IP66	CAT	R1b3*	14	12	14	16	24	11	13	13	11	11	12	14	12	13	10	12	11	11,15
IP68	CAT	R1b3*	14	12	13	16	23	11	14	14	11	11	12	15	12	12	10	12	11	11,14
IP70	CAT	R1b3*	14	12	13	17	24	10	13	13	11	11	12	14	12	11	11	12	12	11,14
IP72	CAT	R1b3*	14	12	13	16	24	11	12	12	10	11	12	14	12	12	11	11	11	12,14
IP74	CAT	R1b3*	14	12	13	16	23	11	13	13	11	11	12	15	12	12	11	12	12	11,14
IP76	CAT	R1b3*	14	12	14	17	24	11	12	13	11	11	12	14	12	12	12	12	11	11,14
IP77	CAT	R1b3*	14	12	12	16	26	10	13	13	11	11	12	15	12	12	10	12	11	11,14
IP78	CAT	R1b3*	14	12	14	17	24	11	13	13	11	11	12	15	12	11	12	12	11	11,14
IP79	CAT	R1b3*	14	12	13	16	25	11	13	13	11	11	12	15	12	12	11	12	11	11,15
IP518	CAT	R1b3*	14	12	13	17	24	10	14	13	11	11	12	15	13	12	10	12	11	12,14
IP519	CAT	R1b3*	14	12	13	17	24	10	13	13	11	11	12	15	12	12	11	12	11	11,15
IP520	CAT	R1b3*	14	12	13	16	24	11	13	13	11	11	12	14	12	12	11	12	11	11,14
IP523	CAT	R1b3*	14	12	14	16	24	12	13	13	11	11	12	14	12	12	10	12	11	11,15
IP524	CAT	R1b3*	14	12	14	16	24	11	13	13	11	11	12	14	12	12	9	12	11	11,13
IP643	CAT	R1b3*	14	14	14	16	23	11	13	13	11	11	12	14	12	12	9	12	11	11,14
IP834	CAT	R1b3*	14	12	13	16	24	10	13	13	11	11	12	15	12	12	11	12	11	11,15
IP539	CAT	R1b3*	14	12	13	17	24	11	13	12	11	11	12	15	12	12	11	11	11	11,14
IP541	CAT	R1b3*	14	12	14	16	24	11	13	14	11	11	12	14	12	11	10	12	11	11,14
IP542	CAT	R1b3*	14	12	14	16	24	11	13	13	11	11	12	15	12	11	11	12	11	12,14
IP545	CAT	R1b3*	14	12	13	16	24	11	13	12	11	11	12	15	12	12	11	11	11	11,14
IP547	CAT	R1b3*	14	12	13	16	25	10	13	12	11	11	12	15	12	12	11	12	11	11,14
IP549	CAT	R1b3*	15	12	14	17	24	11	13	12	11	11	12	15	13	12	11	11	11	11,14
IP550	CAT	R1b3*	14	12	13	16	24	10	13	13	11	11	12	15	12	13	10	12	11	11,15
IP551	CAT	R1b3*	15	12	14	16	24	11	13	13	11	11	12	14	14	12	10	12	11	11,14
IP553	CAT	R1b3*	14	12	12	16	24	11	13	13	11	11	12	15	12	12	11	12	11	11,15
IP554	CAT	R1b3*	14	12	12	16	25	11	13	13	11	11	12	15	12	12	11	12	11	11,15
IP559	CAT	R1b3*	14	12	13	18	25	11	13	13	11	11	12	15	12	12	11	12	11	11,14
IP560	CAT	R1b3*	12	12	13	17	24	10	13	13	11	11	12	15	12	13	11	12	11	11,15
IP561	CAT	R1b3*	15	12	13	16	23	10	13	13	11	11	12	15	12	12	11	12	12	11,14
IP564	CAT	R1b3*	14	12	13	16	24	11	13	12	11	11	12	15	12	12	12	11	11	11,11
IP565	CAT	R1b3*	14	12	13	16	25	10	13	13	11	11	11	15	12	12	10	13	11	11,15
IP566	CAT	R1b3*	14	12	13	16	24	11	13	13	11	11	12	15	12	13	11	12	11	11,14
IP568	CAT	R1b3*	14	12	12	16	25	11	13	13	11	11	12	15	12	12	11	12	11	11,15
IP590	CAT	R1b3*	14	12	14	16	24	10	13	13	11	11	12	15	12	11	10	12	11	11,15
IP591	CAT	R1b3*	14	12	13	17	24	10	13	13	11	11	12	15	11	12	11	12	11	11,14
IP592	CAT	R1b3*	14	12	13	16	23	10	13	13	11	11	12	15	12	11	11	12	11	11,13
IP600	CAT	R1b3*	14	12	14	16	24	10	13	13	11	11	12	14	12	12	10	12	11	11,14

Name	Pop.	Hg	DYS19	DYS388	DYS389I	389 II-Ii	DYS390	DYS391	DYS392	DYS393	DYS434	DYS435	DYS436	DYS437	DYS438	DYS439	DYS460	DYS461	DYS462	DYS385
IP552	CAT	R1b3d	14	12	13	17	23	11	13	13	11	11	12	14	12	11	11	12	11	11,13
IP51	CAT	R1b3f	14	12	13	16	24	11	13	13	11	11	12	15	12	12	11	12	11	11,14
IP69	CAT	R1b3f	14	12	13	16	24	10	13	13	11	11	12	15	12	11	11	12	11	11,15
IP71	CAT	R1b3f	14	12	13	16	24	11	13	13	11	11	12	15	12	12	11	12	11	12,15
IP75	CAT	R1b3f	14	12	13	16	24	10	13	13	11	11	12	15	12	12	11	12	11	12,16
IP80	CAT	R1b3f	15	12	13	17	24	11	13	13	11	11	12	15	12	12	11	13	11	11,14
IP81	CAT	R1b3f	14	12	13	16	24	10	13	14	11	11	12	15	12	12	11	13	11	11,14
IP538	CAT	R1b3f	14	12	13	18	24	12	13	13	11	12	12	15	12	12	12	12	11	11,14
IP540	CAT	R1b3f	14	12	13	16	24	11	13	13	11	11	12	15	12	13	12	12	11	11,14
IP544	CAT	R1b3f	14	12	13	16	24	11	12	13	11	11	12	15	12	12	12	13	11	11,14
IP556	CAT	R1b3f	14	12	13	16	24	10	13	13	11	11	12	15	12	12	11	12	11	11,14
IP558	CAT	R1b3f	14	12	13	16	24	11	12	14	11	11	12	15	12	11	11	11	11	11,14
IP562	CAT	R1b3f	14	12	12	16	24	11	13	13	11	11	12	15	12	13	10	13	11	11,15
IP563	CAT	R1b3f	14	12	13	16	24	11	13	13	11	11	12	15	12	12	12	12	11	11,14
IP569	CAT	R1b3f	14	12	13	16	23	11	13	13	11	11	12	15	12	14	11	12	11	11,14
IP589	CAT	R1b3f	14	12	13	16	25	11	13	13	11	11	12	15	12	12	11	12	11	11,14
IP603	CAT	R1b3f	14	12	14	16	24	11	13	13	11	11	12	15	12	11	11	12	11	11,14
IP604	CAT	R1b3f	14	12	13	16	24	11	13	13	11	11	12	15	12	11	11	12	11	11,15
**Castilla La Mancha**																				
IP381	CLM	E3b1	14	12	12	17	24	10	11	13	11	11	12	14	10	11	11	12	12	16,17
IP676	CLM	E3b2	13	12	14	16	24	9	11	13	11	11	12	14	10	10	10	13	12	13,14
IP250	CLM	G	15	12	12	16	22	10	11	13	11	11	12	16	10	11	11	11	13	15,15
IP267	CLM	G	15	13	12	17	23	10	11	14	11	11	12	16	10	13	10	11	12	14,14
IP333	CLM	G	15	12	12	17	23	10	11	14	11	11	12	15	10	10	11	10	12	14,14
IP492	CLM	G	16	12	13	16	22	10	11	14	11	11	12	15	10	13	11	8	12	14,15
IP706	CLM	G	15	12	12	17	23	10	11	13	11	11	12	16	10	12	11	11	12	14,15
IP742	CLM	G	15	12	12	17	23	10	11	14	11	12	12	16	10	12	10	12	12	14,14
IP264	CLM	I	16	13	13	15	23	10	11	13	11	11	12	15	10	11	10	11	12	12,12
IP638	CLM	J(xJ2)	14	16	13	18	23	11	11	12	11	11	12	14	10	13	11	11	11	13,17
IP335	CLM	J2	15	15	13	18	23	10	11	12	11	11	12	15	9	11	10	13	11	13,13
IP336	CLM	J2	13	15	14	19	23	11	11	12	11	11	12	15	9	10	11	12	11	14,17
IP345	CLM	J2	14	14	14	17	24	10	11	12	11	11	12	15	9	12	10	12	11	13,13
IP347	CLM	J2	15	17	13	18	21	10	11	12	11	11	12	15	9	10	10	13	11	14,15
IP263	CLM	K(xP)	15	12	13	16	23	11	13	13	11	11	12	14	9	11	11	11	14	14,16
IP677	CLM	K(xP)	14	12	13	16	24	11	13	13	11	11	12	15	12	12	11	12	11	11,14
IP379	CLM	R1a1	15	12	13	18	25	11	11	14	11	11	12	14	11	10	8	11	11	11,14
IP280	CLM	R1b3*	14	12	13	16	25	11	13	13	12	11	12	15	12	11	11	12	11	11,14
IP293	CLM	R1b3*	14	12	13	16	24	10	13	13	11	11	12	15	12	12	10	12	11	11,14
IP300	CLM	R1b3*	13	12	14	16	24	13	13	14	11	11	12	14	12	11	10	12	11	11,14
IP302	CLM	R1b3*	14	12	12	16	23	11	13	13	11	11	12	15	12	12	10	12	11	11,15
IP304	CLM	R1b3*	14	12	14	17	24	11	13	13	11	11	12	14	9	13	10	12	11	11,14
IP305	CLM	R1b3*	14	12	13	15	24	11	13	13	11	11	12	15	12	13	10	12	11	11,14

Name	Pop.	Hg	DYS19	DYS388	DYS389I	389 II-Ii	DYS390	DYS391	DYS392	DYS393	DYS434	DYS435	DYS436	DYS437	DYS438	DYS439	DYS460	DYS461	DYS462	DYS385
IP307	CLM	R1b3*	14	12	14	18	24	11	13	13	11	11	12	15	12	13	11	11	11	11,14
IP308	CLM	R1b3*	14	12	14	16	23	10	13	13	11	11	12	15	12	13	11	12	11	11,11
IP318	CLM	R1b3*	14	12	14	16	24	10	12	13	11	11	12	14	13	13	10	12	11	11,12
IP323	CLM	R1b3*	14	12	13	15	24	11	13	13	11	11	12	15	11	13	11	12	11	11,14
IP324	CLM	R1b3*	14	12	14	16	23	10	13	13	11	11	12	15	12	13	11	12	11	11,11
IP325	CLM	R1b3*	14	12	14	17	24	11	12	13	11	11	12	14	12	13	10	12	11	11,14
IP338	CLM	R1b3*	14	12	13	16	24	10	13	13	11	11	12	15	12	12	11	12	11	10,14
IP348	CLM	R1b3*	14	12	13	16	24	10	13	12	11	11	12	15	12	12	11	12	11	11,14
IP351	CLM	R1b3*	14	12	12	16	24	10	14	13	11	11	12	14	12	12	10	13	11	11,14
IP361	CLM	R1b3*	15	12	14	16	24	10	13	13	11	11	12	15	12	13	11	12	11	11,14
IP362	CLM	R1b3*	15	12	13	16	24	11	13	12	11	11	12	15	12	13	10	11	11	11,14
IP367	CLM	R1b3*	14	12	13	17	25	11	13	13	12	11	12	15	12	12	11	12	11	12,14
IP368	CLM	R1b3*	14	12	13	17	24	10	13	13	11	11	12	15	12	12	11	12	11	11,14
IP370	CLM	R1b3*	14	12	13	18	24	11	13	13	11	11	12	15	12	11	11	12	11	11,14
IP372	CLM	R1b3*	15	12	13	16	23	11	13	13	11	11	12	15	12	12	11	12	11	11,14
IP375	CLM	R1b3*	14	12	14	17	24	11	13	13	11	11	12	14	12	11	10	12	11	11,15
IP376	CLM	R1b3*	14	12	12	16	23	11	13	13	11	11	12	15	12	12	10	13	11	11,14
IP466	CLM	R1b3*	15	12	13	16	24	11	13	13	11	11	12	15	12	13	10	12	11	11,14
IP487	CLM	R1b3*	14	12	13	16	24	11	13	13	11	11	12	14	12	13	11	13	11	11,14
IP488	CLM	R1b3*	15	12	13	16	23	11	13	13	11	11	12	15	12	12	10	12	11	11,15
IP490	CLM	R1b3*	14	12	14	16	24	11	13	13	11	11	12	14	12	12	10	12	11	12,14
IP496	CLM	R1b3*	15	12	13	16	24	11	15	13	11	11	12	15	13	11	12	12	11	13,14
IP661	CLM	R1b3*	14	12	13	16,17	24	11	13	13	11	11	12	15	12	10,13	11	13	11	12,14
IP667	CLM	R1b3*	15	12	12	16	24	11	13	13	11	11	12	14	12	12	10	12	11	11,15
IP698	CLM	R1b3*	14	12	13	16	24	11	13	13	11	11	12	14	12	13	10	11	11	11,13
IP703	CLM	R1b3*	15	12	13	16	23	11	13	13	11	11	12	15	12	13	11	12	11	11,14
IP721	CLM	R1b3*	14	12	13	16	24	11	13	13	11	11	12	15	12	11	12	13	11	11,14
IP766	CLM	R1b3*	14	12	13	16	25	11	13	14	11	11	12	14	12	12	10	11	11	12,15
IP790	CLM	R1b3*	14	12	13	16	24	11	13	13	11	11	12	15	12	13	10	12	11	11,14
IP800	CLM	R1b3*	14	12	13	16	23	10	13	13	11	11	12	14	12	12	10	12	11	11,14
IP826	CLM	R1b3*	14	12	13	16	23	11	13	13	11	11	12	15	12	12	10	12	11	11,14
IP854	CLM	R1b3*	14	12	14	16	24	10	13	13	11	11	12	15	12	12	11	12	11	11,14
IP863	CLM	R1b3*	14	12	14	16	24	10	13	13	11	11	12	14	12	12	10	13	11	11,14
IP873	CLM	R1b3*	14	12	14	16	24	12	13	13	11	11	12	14	12	12	10	12	11	11,14
IP312	CLM	R1b3d	14	12	13	16	24	10	13	13	11	11	12	14	12	13	10	12	11	11,14
IP717	CLM	R1b3d	14	12	13	17	24	11	13	12	12	11	12	14	12	12	11	13	11	11,14
IP320	CLM	R1b3f	14	12	14	16	23	11	13	13	11	11	12	15	12	13	11	12	11	11,14
IP628	CLM	R1b3f	14	12	13	16	24	11	13	13	11	11	12	15	12	12	12	13	11	11,14
IP635	CLM	R1b3f	14	12	13	17	25	11	13	13	11	11	12	15	12	12	10	12	11	11,14
IP648	CLM	R1b3f	14	12	13	16	24	11	13	13	11	11	12	15	12	11	10	12	11	11,14
**East Andalusia**																				
IP758	EAN	E3b1	13	12	12	16	24	10	11	13	11	11	12	14	10	11	9	12	12	17,19

Name	Pop.	Hg	DYS19	DYS388	DYS389I	389 II-Ii	DYS390	DYS391	DYS392	DYS393	DYS434	DYS435	DYS436	DYS437	DYS438	DYS439	DYS460	DYS461	DYS462	DYS385
IP463	EAN	E3b2	13	12	14	16	24	9	11	13	11	11	12	14	10	10	11	13	12	13,14
IP734	EAN	E3b2	13	12	14	16	24	10	11	13	11	11	12	14	10	10	11	13	12	13,14
IP231	EAN	E3b3	13	12	13	17	24	10	11	14	11	11	12	14	11	12	10	13	12	16,19
IP277	EAN	G	15	13	13	17	22	10	11	13	11	11	12	16	10	11	10	11	12	15,15
IP326	EAN	G	15	12	12	17	23	10	11	13	11	11	12	16	10	11	10	11	11	14,14
IP842	EAN	G	15	12	12	17	22	10	11	14	11	11	12	16	10	13	11	11	12	12,15
IP484	EAN	I	16	13	13	15	23	10	11	13	11	11	12	15	10	11	10	11	12	12,12
IP502	EAN	I	17	13	14	15	25	9	11	13	11	11	12	15	10	12	10	12	12	12,12
IP503	EAN	I	14	12	13	17	23	10	12	14	11	11	12	14	10	11	11	12	12	15,16
IP727	EAN	I	17	13	13	15	23	10	11	13	11	11	12	15	10	11	10	11	12	12,12
IP753	EAN	I	16	13	12	16	23	10	11	14	11	11	12	15	8	11	11	12	12	15,15
IP756	EAN	I	17	13	14	15	25	9	12	13	11	11	12	15	10	11	10	12	12	12,12
IP684	EAN	J(xJ2)	14	15	13	18	22	10	11	12	11	11	12	14	10	12	11	11	11	14,18
IP724	EAN	J(XJ2)	14	14	14	16	23	10	11	13	10	11	11	14	10	11	11	12	12	12,12
IP736	EAN	J(xJ2)	14	16	13	16	24	10	11	12	11	11	12	14	10	11	11	11	11	12,18
IP273	EAN	J2	14	15	13	17	23	11	11	12	11	11	12	15	9	12	11	12	11	14,18
IP278	EAN	J2	14	15	13	16	23	9	11	13	11	11	12	16	7	11	10	13	11	14,14
IP481	EAN	J2	15	15	13	16	23	10	11	12	11	11	12	15	9	11	10	13	11	13,18
IP498	EAN	J2	15	15	13	16	23	9	11	12	11	11	12	14	9	12	11	12	10	13,16
IP779	EAN	J2	14	15	12	16	24	11	11	12	11	11	12	16	9	12	11	13	11	13,16
IP794	EAN	J2	14	15	13	16	23	9	11	13	11	11	12	16	7	11	10	13	11	14,14
IP827	EAN	J2	14	14	13	17	25	10	11	12	11	11	12	15	9	12	11	14	11	15,16
IP831	EAN	J2	15	14	13	16	23	9	11	12	11	11	12	14	9	12	10	13	10	13,18
IP848	EAN	J2	15	14	14	15	22	10	11	12	11	11	12	15	9	11	10	12	11	13,18
IP506	EAN	R1a1	15	12	13	17	25	11	11	13	11	11	12	14	11	11	11	11	11	11,15
IP235	EAN	R1b3*	14	13	13	16	23	11	13	13	11	11	12	15	13	12	10	12	11	11,14
IP238	EAN	R1b3*	14	12	13	15	24	11	13	13	12	11	12	15	12	8	10	12	11	9,14
IP252	EAN	R1b3*	14	12	12	16	24	12	13	13	11	11	12	15	12	13	11	12	10	11,15
IP254	EAN	R1b3*	14	12	14	17	24	11	13	13	11	11	12	14	12	12	11	13	11	11,15
IP255	EAN	R1b3*	14	12	13	16	24	11	13	13	11	11	12	14	12	12	11	12	11	11,13
IP257	EAN	R1b3*	14	12	14	16	24	11	14	13	11	11	12	14	12	12	11	12	11	11,14
IP258	EAN	R1b3*	15	12	13	16	24	11	13	13	11	11	12	14	12	11	11	12	11	12,15
IP286	EAN	R1b3*	14	12	12	16	23	11	13	13	11	11	12	15	12	13	11	12	11	11,14
IP311	EAN	R1b3*	14	12	14	16	24	11	13	13	11	11	12	15	12	12	11	12	11	11,15
IP322	EAN	R1b3*	14	12	12	15	23	11	14	12	11	11	12	15	12	14	11	11	11	11,14
IP332	EAN	R1b3*	14	12	14	16	24	10	13	13	11	11	12	15	12	11	11	12	11	11,14
IP344	EAN	R1b3*	14	12	13	16	24	11	13	12	11	11	12	15	12	12	11	12	11	11,14
IP349	EAN	R1b3*	14	12	14	17	24	11	13	13	11	11	12	15	12	11	11	12	11	11,14
IP358	EAN	R1b3*	14	12	13	16	24	11	13	13	11	11	12	15	12	11	11	12	11	12,14
IP365	EAN	R1b3*	14	12	13	16	25	11	13	12	11	11	13	15	12	11	8	11	11	11,14
IP383	EAN	R1b3*	14	12	13	16	24	11	13	12	11	11	12	15	12	12	11	12	11	11,14
IP460	EAN	R1b3*	14	12	14	16	24	11	13	13	11	11	12	14	9	11	10	12	11	11,14
IP479	EAN	R1b3*	14	12	14	16	23	10	13	13	11	11	12	14	12	13	11	12	11	11,14

Name	Pop.	Hg	DYS19	DYS388	DYS389I	389 II-Ii	DYS390	DYS391	DYS392	DYS393	DYS434	DYS435	DYS436	DYS437	DYS438	DYS439	DYS460	DYS461	DYS462	DYS385
IP480	EAN	R1b3*	14	12	13	16	24	11	13	13	11	11	12	14	12	12	10	12	11	11,14
IP482	EAN	R1b3*	14	12	13	16	24	11	13	13	11	11	12	15	12	12	10	12	11	11,15
IP485	EAN	R1b3*	14	12	14	16	25	11	13	13	11	11	12	15	12	12	11	11	11	11,14
IP491	EAN	R1b3*	14	12	13	16	24	11	13	13	11	11	12	15	12	12	11	12	11	11,14
IP497	EAN	R1b3*	14	12	13	16	24	11	13	13	11	11	12	15	12	12	11	13	11	11,14
IP499	EAN	R1b3*	15	12	13	16	24	11	13	13	11	11	12	15	12	12	10	12	11	11,14
IP500	EAN	R1b3*	14	12	13	16	24	10	13	14	11	11	12	14	12	12	11	12	11	11,14
IP504	EAN	R1b3*	14	12	13	16	25	11	13	13	11	11	12	15	12	12	10	12	11	11,14
IP505	EAN	R1b3*	14	12	13	16	23	10	13	13	11	11	12	15	12	12	10	12	11	13,14
IP507	EAN	R1b3*	14	12	13	16	24	11	13	13	11	11	12	15	12	11	11	12	12	11,15
IP508	EAN	R1b3*	14	12	13	17	24	11	13	13	11	11	12	15	12	13	11	13	11	15,15
IP510	EAN	R1b3*	14	12	13	16	24	11	13	13	11	11	12	15	12	12	11	12	11	11,15
IP511	EAN	R1b3*	14	12	14	17	23	10	13	13	11	11	12	15	12	12	10	12	11	11,11
IP651	EAN	R1b3*	14	12	13	16	23	10	13	13	11	11	12	15	12	11	11	12	11	11,14
IP653	EAN	R1b3*	14	12	13	17	23	10	13	13	11	11	12	14	12	13	11	12	11	11,14
IP671	EAN	R1b3*	14	12	13	16	24	10	13	13	11	11	12	14	12	11	10	11	11	11,14
IP679	EAN	R1b3*	14	12	13	17	24	11	13	13	11	11	12	15	12	12	10	12	11	11,14
IP691	EAN	R1b3*	12	12	14	16	24	11	13	13	11	11	12	14	12	12	10	12	11	11,14
IP710	EAN	R1b3*	14	12	14	16	25	11	13	13	11	11	12	15	12	11	11	12	11	11,14
IP720	EAN	R1b3*	14	12	13	15	24	11	14	13	11	11	12	15	12	12	11	11	11	11,14
IP722	EAN	R1b3*	14	12	14	19	25	11	13	13	11	11	12	15	12	11	10	12	11	11,14
IP729	EAN	R1b3*	14	12	12	16	24	11	14	13	11	11	12	14	12	12	10	12	11	11,14
IP731	EAN	R1b3*	14	12	13	16	24	11	13	13	11	11	12	15	12	12	11	12	11	11,14
IP745	EAN	R1b3*	14	12	13	16	24	11	13	13	11	11	12	14	12	12	11	12	11	11,14
IP746	EAN	R1b3*	14	12	12	16	24	10	13	13	11	11	12	15	12	12	11	12	11	11,14
IP751	EAN	R1b3*	14	12	13	16	24	10	13	13	11	11	12	15	12	13	10	11	11	11,14
IP764	EAN	R1b3*	14	12	14	15	24	11	14	13	11	11	12	16	12	11	11	12	12	11,14
IP770	EAN	R1b3*	14	12	15	16	23	11	13	13	11	11	12	15	12	12	11	11	11	11,15
IP778	EAN	R1b3*	14	12	13	16	24	11	13	13	11	11	12	15	12	11	11	11	11	11,14
IP781	EAN	R1b3*	14	12	14	16	24	11	13	13	11	11	12	15	12	10	11	12	11	11,14
IP785	EAN	R1b3*	14	12	13	16	23	11	13	13	11	11	12	15	12	12	11	12	11	11,13
IP796	EAN	R1b3*	14	12	14	16	24	11	13	13	12	11	12	14	12	12	10	13	11	11,14
IP798	EAN	R1b3*	14	12	14	17	24	11	13	13	11	11	12	15	12	11	11	12	11	11,16
IP801	EAN	R1b3*	14	12	13	16	24	11	13	13	11	11	12	15	12	11	11	12	11	11,14
IP819	EAN	R1b3*	14	12	13	16	23	11	13	13	11	11	12	15	12	11	11	12	11	11,14
IP820	EAN	R1b3*	14	12	13	16	24	12	13	13	11	11	12	15	10	12	11	12	11	11,14
IP823	EAN	R1b3*	14	12	13	16	23	11	13	13	11	11	12	14	12	11	11	12	12	11,14
IP832	EAN	R1b3*	14	12	13	16	24	10	13	12	11	11	12	15	12	13	10	12	11	11,14
IP838	EAN	R1b3*	14	12	13	16	23	11	13	13	11	11	12	14	12	11	11	12	11	11,14
IP844	EAN	R1b3*	14	12	13	16	24	10	13	13	11	11	12	15	12	11	11	13	11	11,13
IP847	EAN	R1b3*	14	12	13	16	24	11	13	13	11	11	12	15	12	12	11	13	11	10,15
IP297	EAN	R1b3d	14	12	13	16	23	10	13	13	11	11	12	14	12	12	10	12	11	11,13
IP723	EAN	R1b3d	14	12	13	16	24	10	13	13	11	11	12	14	12	12	10	11	11	11,14

Name	Pop.	Hg	DYS19	DYS388	DYS389I	389 II-Ii	DYS390	DYS391	DYS392	DYS393	DYS434	DYS435	DYS436	DYS437	DYS438	DYS439	DYS460	DYS461	DYS462	DYS385
IP743	EAN	R1b3d	14	12	13	16	24	10	13	13	12	11	12	14	12	12	10	11	11	11,14
IP476	EAN	R1b3f	14	12	14	16	24	11	12	13	11	11	12	15	12	13	11	12	11	11,14
IP665	EAN	R1b3f	14	12	13	17	24	10	13	13	11	11	12	15	12	13	11	12	11	11,15
IP712	EAN	R1b3f	14	12	13	16	24	11	13	13	11	11	12	15	12	12	11	12	11	11,15
IP761	EAN	R1b3f	14	12	14	16	23	11	13	13	11	11	12	14	12	13	11	12	11	11,14
IP772	EAN	R1b3f	14	12	13	16	24	11	13	13	11	11	12	15	12	13	11	12	11	11,14
IP858	EAN	R1b3f	14	12	13	18	24	11	13	13	11	11	12	16	12	12	11	12	11	11,14
IP875	EAN	R1b3f	14	12	14	16	24	10	12	13	11	11	12	15	12	13	11	12	11	11,14
**Extremadura**																				
IP256	EXT	E3b1	13	13	13	17	24	10	11	13	11	11	12	14	10	12	9	12	12	16,18
IP262	EXT	E3b1	14	12	13	17	25	10	11	13	11	11	12	14	10	12	11	12	12	17,18
IP719	EXT	E3b1	13	12	13	17	24	10	11	13	11	11	12	14	10	13	10	12	12	17,17
IP868	EXT	E3b1	13	12	12	17	24	10	11	13	11	11	12	14	9	10	10	13	13	16,17
IP244	EXT	E3b2	13	12	13	16	25	9	11	13	11	11	12	14	10	10	11	13	12	14,14
IP245	EXT	E3b2	13	13	13	16	24	9	11	13	11	11	12	14	10	10	11	13	12	13,14
IP352	EXT	E3b2	13	13	13	16	24	9	11	13	11	11	12	14	10	10	11	13	12	13,14
IP750	EXT	E3b2	13	12	15	17	24	9	11	13	11	11	12	14	10	10	11	14	12	13,14
IP330	EXT	E3b3	13	12	13	16	24	9	11	13	12	11	12	14	10	10	11	14	12	13,14
IP339	EXT	F*	14	13	14	16	23	10	11	12	11	11	12	14	10	11	11	13	12	13,14
IP478	EXT	G	15	13	12	18	22	10	11	14	11	11	12	16	10	13	11	11	12	12,14
IP769	EXT	G	15	12	12	17	23	10	12	14	11	11	12	16	9	11	11	11	12	14,14
IP265	EXT	I	16	13	13	15	26	9	11	13	11	11	12	15	10	11	10	12	12	12,12
IP268	EXT	I	14	15	12	17	22	10	11	14	11	11	12	16	10	11	10	12	12	13,14
IP299	EXT	I	14	14	12	16	23	10	11	13	11	11	12	16	10	12	10	12	12	14,14
IP857	EXT	I	15	13	12	17	23	10	12	15	11	11	12	15	10	11	11	12	12	14,15
IP874	EXT	I	14	14	12	16	23	10	11	13	11	11	12	16	10	11	10	12	13	14,14
IP281	EXT	J2	15	16	13	17	23	10	11	12	11	11	12	14	9	11	10	12	10	13,18
IP329	EXT	J2	16	16	13	17	23	11	11	12	11	11	12	15	9	14	10	13	11	12,18
IP773	EXT	J2	15	15	12	15	23	10	11	13	11	11	12	16	9	12	12	10	11	18,18
IP784	EXT	J2	15	16	13	17	23	10	11	12	11	11	12	14	9	11	10	12	10	13,18
IP795	EXT	J2	14	15	13	16	23	9	11	13	11	11	12	16	7	11	10	13	11	14,14
IP817	EXT	J2	14	15	13	17	23	10	11	12	11	11	12	14	9	11	10	13	12	13,16
IP371	EXT	K(xP)	13	12	13	16	23	10	13	13	11	11	12	15	9	13	10	11	12	14,16
IP755	EXT	K(xP)	15	12	13	17	25	10	14	13	11	11	12	14	9	11	10	11	12	14,16
IP815	EXT	K(xP)	14	12	13	16	23	10	15	13	11	11	12	14	8	11	11	12	12	15,17
IP233	EXT	R1b3*	15	12	13	16	23	11	13	13	11	11	12	15	10	12	11	12	11	11,14
IP237	EXT	R1b3*	15	12	13	16	24	11	13	14	11	11	12	15	12	12	11	12	11	11,15
IP242	EXT	R1b3*	13	12	13	17	22	10	13	13	11	11	12	15	12	12	11	12	11	12,14
IP261	EXT	R1b3*	14	12	13	15	23	10	13	13	11	11	12	15	12	11	12	12	11	11,14
IP346	EXT	R1b3*	14	12	13	16	24	11	13	13	11	11	12	15	13	11	11	12	11	11,15
IP357	EXT	R1b3*	14	12	14	17	24	11	13	13	11	11	12	15	12	12	10	12	12	12,14
IP366	EXT	R1b3*	15	12	13	18	24	13	13	13	11	11	12	15	12	12	11	12	11	11,15

Name	Pop.	Hg	DYS19	DYS388	DYS389I	389 II-Ii	DYS390	DYS391	DYS392	DYS393	DYS434	DYS435	DYS436	DYS437	DYS438	DYS439	DYS460	DYS461	DYS462	DYS385
IP477	EXT	R1b3*	14	12	13	16	24	10	13	13	11	11	12	16	12	12	10	12	11	11,13
IP483	EXT	R1b3*	15	12	13	16	23	11	13	13	11	11	12	15	12	12	11	12	11	11,14
IP494	EXT	R1b3*	15	12	14	16	24	10	13	13	11	11	12	14	12	13	10	12	11	11,13
IP501	EXT	R1b3*	14	12	13	16	23	11	13	13	11	11	12	15	13	12	11	11	11	11,14
IP739	EXT	R1b3*	14	12	14	16	24	11	14	13	11	11	12	15	12	12	11	12	11	11,14
IP744	EXT	R1b3*	14	12	13	16	24	11	13	13	11	11	12	15	12	14	11	12	11	11,14
IP760	EXT	R1b3*	14	12	14	16	24	10	13	13	11	11	12	14	12	12	10	12	11	11,14
IP771	EXT	R1b3*	12	12	13	17	24	10	13	13	11	11	12	15	12	12	11	12	11	11,15
IP807	EXT	R1b3*	12	12	13	17	24	10	14	13	11	11	12	15	12	12	11	12	11	11,15
IP811	EXT	R1b3*	14	14	14	16	24	10	13	13	11	11	12	15	12	11	11	12	11	11,14
IP816	EXT	R1b3*	14	12	13	16	24	10	13	13	11	11	12	14	12	12	10	12	11	11,14
IP818	EXT	R1b3*	14	12	14	17	24	11	13	13	11	11	12	14	12	12	10	12	11	11,14
IP822	EXT	R1b3*	14	12	14	16	24	11	13	13	11	11	12	14	12	12	10	12	12	10,13
IP829	EXT	R1b3*	14	12	13	16	24	11	13	13	11	11	12	15	12	13	11	12	11	11,14
IP836	EXT	R1b3*	14	12	13	16	24	10	13	13	11	11	12	15	12	13	11	12	11	12,14
IP846	EXT	R1b3*	15	12	13	16	24	10	13	13	12	11	12	14	12	12	11	12	11	11,13
IP856	EXT	R1b3*	14	12	13	16	24	11	13	12	11	11	12	15	12	12	10	12	11	11,14
IP861	EXT	R1b3*	14	12	13	16	24	11	13	13	11	11	12	15	12	11	11	12	11	11,14
IP803	EXT	R1b3f	14	12	14	16	24	9	13	13	11	11	12	15	12	13	10	12	11	11,14
**Galicia**																				
IP409	GAL	E3b*	13	12	13	16	24	10	11	12	11	11	12	14	11	12	10	13	12	16,18
IP234	GAL	E3b1	13	12	13	17	23	10	11	13	11	11	12	14	10	12	11	11	13	15,16
IP397	GAL	E3b1	13	12	13	17	23	10	11	13	11	11	12	14	10	12	11	11	13	16,16
IP416	GAL	E3b1	13	12	13	17	24	10	11	13	11	11	12	14	10	12	9	12	12	16,18
IP683	GAL	E3b1	13	12	14	17	23	10	11	13	11	11	12	14	10	12	11	11	12	15,16
IP765	GAL	E3b1	13	12	13	17	23	10	11	13	11	11	12	14	10	11	9	12	12	18,18
IP777	GAL	E3b1	14	12	13	18	24	10	11	13	11	11	12	14	10	12	11	12	11	17,19
IP387	GAL	E3b2	13	12	13	16	24	9	11	14	11	11	12	14	10	10	11	12	12	13,14
IP399	GAL	E3b2	13	12	14	16	24	9	11	13	11	11	12	14	10	10	12	13	12	12,15
IP413	GAL	E3b2	13	12	14	17	25	9	11	13	11	11	12	14	10	10	11	13	12	13,15
IP419	GAL	E3b2	13	13	14	16	24	9	11	13	11	11	12	14	10	11	11	13	12	14,14
IP725	GAL	E3b2	13	12	14	16	23	9	11	13	11	11	12	14	10	10	11	13	12	13,14
IP740	GAL	E3b2	13	12	14	16	24	9	11	13	11	11	12	14	10	11	12	13	12	13,14
IP799	GAL	E3b2	13	12	14	16	23	10	12	13	11	11	12	14	10	10	11	12	12	13,14
IP855	GAL	E3b2	13	12	14	16	23	9	11	13	11	11	12	14	10	10	10	13	12	13,14
IP294	GAL	G	15	13	12	16	22	10	11	14	11	11	12	16	11	11	9	11	12	14,14
IP400	GAL	G	15	12	12	16	21	10	11	14	11	11	12	16	10	12	10	11	12	13,15
IP406	GAL	G	17	13	13	17	22	10	11	14	11	11	12	16	11	11	11	11	12	14,14
IP699	GAL	G	15	12	12	18	23	10	11	15	11	11	12	16	10	11	11	12	12	13,14
IP718	GAL	G	16	12	12	17	21	10	11	15	11	11	14	16	10	11	10	11	12	13,16
IP276	GAL	I	14	14	12	16	23	10	11	13	11	11	12	16	10	13	10	12	12	13,15
IP282	GAL	I	14	14	12	16	22	10	11	13	11	11	12	16	10	12	10	12	12	13,15

Name	Pop.	Hg	DYS19	DYS388	DYS389I	389 II-Ii	DYS390	DYS391	DYS392	DYS393	DYS434	DYS435	DYS436	DYS437	DYS438	DYS439	DYS460	DYS461	DYS462	DYS385
IP386	GAL	I	16	13	13	16	23	11	12	14	11	11	12	15	10	11	11	12	11	12,16
IP404	GAL	I	15	13	14	18	23	11	12	14	11	11	12	15	10	11	11	12	12	15,15
IP411	GAL	I	14	14	12	16	23	10	11	13	11	11	12	16	10	11	10	12	12	13,15
IP422	GAL	I	16	13	13	16	23	11	12	14	11	11	12	15	10	11	11	12	11	12,16
IP673	GAL	I	15	13	13	17	24	10	12	15	11	11	12	15	10	11	11	12	12	14,15
IP747	GAL	I	14	14	12	17	23	10	11	13	11	11	12	16	10	11	10	12	12	13,15
IP809	GAL	I	14	13	13	19	25	11	11	13	11	11	12	15	10	11	10	11	12	12,15
IP266	GAL	J(xJ2)	14	16	13	17	23	10	11	12	11	11	12	14	10	11	11	11	11	13,16
IP295	GAL	J2	14	15	13	17	22	10	11	12	11	11	12	15	9	10	10	12	11	14,14
IP389	GAL	J2	14	16	12	16	23	10	11	12	11	11	12	14	9	11	11	13	11	13,16
IP423	GAL	J2	15	15	12	16	24	10	11	13	11	11	12	16	9	11	11	10	11	13,17
IP802	GAL	J2	15	16	13	16	23	9	11	13	11	11	12	14	9	12	10	13	10	13,16
IP833	GAL	J2	15	16	13	16	22	10	11	12	11	11	12	15	9	11	10	13	11	14,15
IP872	GAL	J2	15	15	12	16	26	10	11	12	11	11	12	16	9	11	11	10	11	15,18
IP414	GAL	K(xP)	15	12	12	15	24	10	14	13	11	11	12	14	9	11	10	11	13	13,15
IP85	GAL	R1b3*	14	12	13	16	25	11	13	13	11	11	12	15	12	12	11	12	11	12,14
IP227	GAL	R1b3*	14	12	13	16	23	10	13	13	11	11	12	15	12	12	11	11	11	11,13
IP229	GAL	R1b3*	14	12	13	16	24	11	13	12	11	11	12	15	12	12	11	11	11	11,13
IP259	GAL	R1b3*	14	12	13	16	24	11	13	13	11	11	12	15	12	12	11	12	11	11,14
IP270	GAL	R1b3*	14	12	13	16	24	11	13	13	11	11	12	15	12	11	10	13	11	11,15
IP287	GAL	R1b3*	14	12	13	16	24	11	14	13	11	11	12	14	12	12	11	12	11	11,16
IP289	GAL	R1b3*	14	12	12	16	23	11	13	13	11	11	12	15	12	11	10	12	11	11,14
IP317	GAL	R1b3*	14	12	13	16	25	11	14	13	11	11	12	15	12	12	10	12	11	11,14
IP354	GAL	R1b3*	14	12	13	14	24	11	13	14	11	11	13	15	11	12	11	12	11	11,14
IP384	GAL	R1b3*	14	12	13	17	23	10	13	13	11	11	12	14	12	13	11	12	11	11,14
IP385	GAL	R1b3*	15	12	13	16	24	10	13	13	11	11	12	15	12	12	11	12	11	11,15
IP388	GAL	R1b3*	14	12	13	16	24	11	14	14	11	11	12	15	12	12	10	11	11	11,11
IP391	GAL	R1b3*	13	12	13	16	25	11	13	13	11	11	12	15	12	13	11	12	11	12,14
IP392	GAL	R1b3*	14	12	13	15	23	12	13	13	11	11	12	15	12	11	10	11	11	10,14
IP393	GAL	R1b3*	14	12	13	16	24	11	13	13	11	11	12	15	12	13	10	12	11	11,14
IP394	GAL	R1b3*	14	12	13	17	24	11	13	13	11	11	12	15	12	12	10	12	11	11,15
IP395	GAL	R1b3*	14	12	13	16	24	11	13	13	11	11	12	15	12	11	10	12	11	12,14
IP396	GAL	R1b3*	14	12	13	16	24	11	13	12	11	11	12	15	12	12	10	11	11	11,14
IP398	GAL	R1b3*	14	12	13	14	24	11	13	13	11	11	12	15	13	13	10	12	11	11,14
IP401	GAL	R1b3*	14	12	13	16	24	11	13	15	11	11	12	14	12	12	10	11	11	12,15
IP402	GAL	R1b3*	14	12	14	15	24	11	13	13	11	11	12	15	12	13	12	13	11	11,13
IP403	GAL	R1b3*	15	12	13	16	24	10	13	14	11	11	12	15	12	11	11	12	10	11,14
IP407	GAL	R1b3*	14	12	12	15	24	10	13	13	11	11	12	15	11	11	10	12	11	11,16
IP408	GAL	R1b3*	14	12	13	16	24	10	13	13	11	11	12	15	13	13	10	12	11	11,16
IP410	GAL	R1b3*	14	12	13	16	24	11	13	13	11	11	12	15	13	13	10	12	11	11,14
IP415	GAL	R1b3*	15	12	13	16	24	11	13	13	11	11	13	14	13	12	10	12	11	12,14
IP417	GAL	R1b3*	14	12	12	16	24	10	13	13	11	11	12	15	12	12	11	11	11	11,14
IP420	GAL	R1b3*	14	12	13	16	24	11	13	13	11	11	12	15	12	12	10	12	11	11,14

Name	Pop.	Hg	DYS19	DYS388	DYS389I	389 II-Ii	DYS390	DYS391	DYS392	DYS393	DYS434	DYS435	DYS436	DYS437	DYS438	DYS439	DYS460	DYS461	DYS462	DYS385
IP421	GAL	R1b3*	15	12	13	16	24	10	13	13	11	11	12	15	12	12	11	12	11	11,15
IP668	GAL	R1b3*	14	12	13	16	23	12	13	13	11	11	12	15	12	13	11	12	11	12,12
IP669	GAL	R1b3*	14	12	13	16	24	11	13	12	11	11	12	15	12	12	10	12	11	11,12,14
IP694	GAL	R1b3*	14	12	14	17	24	10	13	14	11	11	12	15	12	14	11	12	11	11,13
IP695	GAL	R1b3*	14	12	13	16	24	10	13	13	10	11	12	15	11	12	11	12	11	11,14
IP702	GAL	R1b3*	14	12	15	16	24	11	13	13	11	11	12	15	13	14	10	12	11	11,13
IP704	GAL	R1b3*	14	12	14	16	23	11	13	13	11	11	12	15	12	12	10	12	11	11,14
IP705	GAL	R1b3*	14	12	13	17	24	11	14	13	11	11	12	15	12	13	10	12	12	11,17
IP714	GAL	R1b3*	14	12	13	16	24	10	13	13	11	11	12	15	12	11	11	12	12	11,15
IP715	GAL	R1b3*	14	12	14	17	23	11	13	13	11	11	12	14	12	13	10	12	11	11,14
IP733	GAL	R1b3*	14	12	13	16	23	10	13	13	11	11	12	15	12	12	11	12	11	11,15
IP748	GAL	R1b3*	14	12	13	16	24	11	14	13	11	11	12	14	12	12	11	12	11	11,16
IP763	GAL	R1b3*	14	12	14	16	24	11	13	13	11	11	12	15	12	12	10	12	11	11,14
IP806	GAL	R1b3*	14	12	12	16	24	11	13	12	11	11	12	15	12	12	11	12	11	12,14
IP845	GAL	R1b3*	14	12	13	15	22	10	13	13	11	11	12	15	11	11	11	12	11	11,16
IP865	GAL	R1b3*	14	12	13	16	25	11	14	13	11	11	12	15	12	12	10	12	11	11,14
IP867	GAL	R1b3*	14	12	13	15	25	10	13	14	11	11	12	15	12	13	10	12	11	11,14
IP871	GAL	R1b3*	15	12	13	16	24	11	13	13	11	11	12	14	12	12	11	10	11	11,13
IP390	GAL	R1b3d	14	12	13	17	24	11	13	13	11	11	12	14	12	12	11	12	11	11,15
IP824	GAL	R1b3d	14	12	13	16	25	11	13	13	11	11	12	14	12	11	11	12	11	11,14
IP405	GAL	R1b3f	14	12	12	16	24	11	13	13	11	11	12	15	12	13	11	12	11	11,14
IP412	GAL	R1b3f	14	12	13	16	24	11	13	13	11	11	12	15	12	11	11	12	12	11,14
IP708	GAL	R1b3f	14	12	13	16	23	11	13	13	11	11	12	15	12	12	12	12	11	11,14
**Gascony**																				
IP921	GAS	J2	15	15	12	16	24	10	11	12	11	11	12	16	9	12	11	10	11	14,16
IP587	GAS	R1b3*	14	12	12	16	23	11	13	13	11	11	12	15	12	11	11	13	11	12,15
IP593	GAS	R1b3*	14	12	14	16	24	11	13	13	11	11	12	15	12	12	11	12	11	11,14
IP594	GAS	R1b3*	14	12	14	16	24	11	13	13	11	11	12	15	12	12	11	12	11	11,14
IP595	GAS	R1b3*	14	12	14	16	24	11	13	13	11	11	12	15	12	12	11	12	11	11,14
IP596	GAS	R1b3*	14	12	12	16	24	10	13	13	11	11	12	15	12	12	11	12	11	11,15
IP601	GAS	R1b3*	14	12	13	16	24	10	13	13	11	11	12	14	12	12	10	12	11	10,14
IP605	GAS	R1b3*	14	12	13	16	24	11	13	13	11	11	12	14	12	11	10	12	12	11,14
IP913	GAS	R1b3*	14	12	14	16	24	11	13	13	11	11	12	14	12	12	10	12	11	11,14
IP914	GAS	R1b3*	14	12	13	16	24	10	13	13	11	11	12	15	12	11	11	12	11	11,14
IP915	GAS	R1b3*	14	12	13	16	24	11	13	14	11	11	12	14	12	12	11	12	11	11,13
IP919	GAS	R1b3*	14	12	14	16	24	10	13	13	11	11	12	14	12	12	11	12	11	12,14
IP920	GAS	R1b3*	15	12	13	17	24	11	13	13	11	11	12	14	11	11	10	12	11	11,13
IP922	GAS	R1b3*	14	12	14	16	22	10	13	13	11	11	12	15	12	12	11	13	11	11,11
IP923	GAS	R1b3*	14	12	12	16	24	11	13	13	11	11	12	15	12	12	10	12	11	12,14
IP924	GAS	R1b3*	15	12	13	16	24	11	12	13	11	11	12	15	12	12	11	13	10	11,14
IP912	GAS	R1b3d	14	12	13	16	24	10	13	13	11	11	12	14	12	12	11	12	11	11,14
IP916	GAS	R1b3d	14	12	13	16	24	10	13	13	11	11	12	14	12	12	10	12	11	11,14

Name	Pop.	Hg	DYS19	DYS388	DYS389I	389 II-Ii	DYS390	DYS391	DYS392	DYS393	DYS434	DYS435	DYS436	DYS437	DYS438	DYS439	DYS460	DYS461	DYS462	DYS385
IP917	GAS	R1b3d	14	12	13	16	24	11	13	13	11	11	12	14	12	12	10	12	11	12,14
IP918	GAS	R1b3d	14	12	14	16	24	11	13	14	11	11	12	14	12	11	10	12	11	11,14
IP84	GAS	R1b3f	14	12	13	15	24	10	13	13	11	11	12	15	12	12	11	12	11	11,15
IP597	GAS	R1b3f	14	12	13	15	24	11	13	13	11	11	12	15	12	13	11	12	11	11,14
IP598	GAS	R1b3f	14	12	13	16	24	11	13	13	11	11	12	15	12	12	11	12	11	11,14
IP602	GAS	R1b3f	14	12	13	15	23	11	13	13	11	11	12	15	12	14	10	12	11	11,14
**Northeast Castille**																				
IP341	NEC	E3b*	14	12	13	17	23	9	11	13	11	11	12	14	10	12	10	13	12	12,15
IP342	NEC	E3b1	13	14	13	18	24	11	11	13	11	11	12	14	10	13	10	11	12	15,15
IP364	NEC	E3b2	13	12	14	17	24	9	11	13	11	11	12	14	10	11	11	13	12	13,14
IP788	NEC	G	15	12	13	17	22	10	11	12	11	11	12	16	10	10	10	13	12	12,13
IP428	NEC	I	17	13	13	15	24	9	11	13	11	11	12	15	10	12	10	12	12	12,12
IP447	NEC	J2	14	15	13	16	24	10	11	12	11	11	12	14	9	12	10	12	12	13,15
IP427	NEC	K(xP)	15	12	13	16	23	10	15	13	11	11	12	14	9	10	10	11	13	16,17
IP86	NEC	R1b3*	14	12	13	16	24	11	13	14	11	11	12	15	12	13	11	12	11	11,15
IP319	NEC	R1b3*	14	12	13	16	23	10	13	13	11	11	12	15	12	12	11	12	11	12,14
IP321	NEC	R1b3*	15	12	13	16	25	11	13	13	11	11	12	15	12	11	11	12	11	10,14
IP378	NEC	R1b3*	14	12	13	17	24	11	13	13	11	11	12	14	12	11	10	12	11	11,15
IP424	NEC	R1b3*	14	12	13	16	23	11	13	13	11	11	12	15	12	12	10	12	11	11,14
IP425	NEC	R1b3*	14	12	13	18	24	11	13	13	11	11	12	15	12	13	11	12	11	11,12
IP426	NEC	R1b3*	14	11	13	16	24	11	13	13	11	11	12	15	12	13	11	11	11	12,14
IP429	NEC	R1b3*	14	12	13	16	25	12	13	13	11	11	12	15	12	12	10	12	11	11,15
IP430	NEC	R1b3*	15	12	13	16	24	11	13	13	11	11	12	15	12	11	10	12	12	11,14
IP431	NEC	R1b3*	14	12	12	16	24	11	13	13	11	11	12	15	12	12	11	12	11	11,14
IP432	NEC	R1b3*	14	12	13	16	24	11	13	13	11	11	12	14	12	12	10	12	11	11,14
IP433	NEC	R1b3*	14	12	14	16	24	10	13	14	11	11	12	14	12	13	10	12	12	11,14
IP435	NEC	R1b3*	13	12	13	16	25	12	13	13	11	11	12	15	12	13	11	12	11	11,14
IP436	NEC	R1b3*	15	12	12	16	25	11	13	13	11	11	12	15	12	11	11	12	11	12,14
IP437	NEC	R1b3*	14	12	13	16	25	11	13	13	11	11	12	15	12	12	11	12	11	11,14
IP446	NEC	R1b3*	14	12	13	16	24	11	13	13	11	11	12	15	12	11	11	12	11	12,14
IP681	NEC	R1b3*	14	12	13	16	24	11	13	13	11	11	12	15	12	13	10	12	11	11,14
IP732	NEC	R1b3*	14	12	14	16	24	11	13	13	11	11	12	15	12	13	10	12	11	11,14
IP735	NEC	R1b3*	14	12	13	16	24	11	13	13	11	11	12	15	12	12	10	12	11	11,13
IP775	NEC	R1b3*	14	12	13	15	23	10	13	13	11	11	12	15	12	12	10	12	10	11,14
IP787	NEC	R1b3*	14	13	13	16	24	11	13	13	11	11	12	15	12	12	11	12	12	10,15
IP862	NEC	R1b3*	14	12	14	16	24	12	13	13	11	11	12	15	12	12	11	12	11	11,14
IP434	NEC	R1b3d	14	12	14	15	23	11	13	13	11	11	12	14	12	11	10	12	11	11,14
IP285	NEC	R1b3f*	15	12	13	16	24	10	13	13	11	11	12	15	12	12	11	13	11	11,14
**North Portugal**																				
IP118	NPO	E1	17	12	12	17	22	9	12	13	11	12	12	15	11	13	10	13	12	14,16
IP124	NPO	E1	17	12	12	17	22	9	12	13	11	12	12	16	10	13	10	13	12	15,16

Name	Pop.	Hg	DYS19	DYS388	DYS389I	389 II-Ii	DYS390	DYS391	DYS392	DYS393	DYS434	DYS435	DYS436	DYS437	DYS438	DYS439	DYS460	DYS461	DYS462	DYS385
IP113	NPO	E3b*	14	12	13	17	25	10	11	14	11	11	12	14	10	11	11	12	13	16,17
IP91	NPO	E3b1	13	12	13	17	24	10	11	13	11	11	12	14	10	12	10	12	12	16,18
IP103	NPO	E3b1	13	12	13	16	24	10	11	13	11	11	12	15	10	12	9	12	12	16,18
IP138	NPO	E3b1	13	12	11	18	25	10	12	13	10	11	12	14	10	11	11	11	12	16,19
IP95	NPO	E3b2	13	12	13	17	23	9	11	13	11	11	12	14	10	10	11	13	12	13,14
IP114	NPO	E3b2	13	12	14	16	24	9	11	13	11	11	12	14	10	11	11	12	12	13,14
IP143	NPO	E3b3	13	12	13	19	24	10	11	13	11	11	12	14	10	14	10	11	10	15,16
IP87	NPO	G	15	12	12	17	22	10	11	15	11	11	14	16	10	11	10	11	12	13,17
IP89	NPO	G	15	12	13	16	24	10	13	13	11	11	12	14	12	12	11	12	11	11,14
IP94	NPO	G	15	12	12	17	22	10	11	13	11	11	12	16	10	12	11	11	12	12,15
IP104	NPO	G	16	12	12	16	21	11	11	14	11	11	12	16	10	12	10	11	12	15,15
IP107	NPO	G	15	12	12	17	22	10	11	14	11	11	12	16	10	12	11	11	12	12,16
IP127	NPO	G	15	12	12	17	22	10	11	13	11	11	12	16	11	14	11	10	12	15,15
IP133	NPO	G	15	12	11	17	23	11	11	14	11	11	12	16	10	12	10	11	12	13,14
IP111	NPO	I	16	13	14	16	24	11	12	14	11	11	12	15	8	12	11	12	12	15,15
IP93	NPO	J(xJ2)	14	13	13	16	24	10	11	13	11	11	11	14	10	13	11	12	12	13,19
IP109	NPO	J2	15	16	13	17	23	10	11	12	11	11	12	15	9	11	10	8	11	13,15
IP117	NPO	J2	15	15	13	16	24	10	11	12	11	11	12	16	9	13	11	10	11	15,17
IP136	NPO	J2	15	15	12	16	24	10	11	13	11	11	12	16	9	11	11	10	11	13,17
IP139	NPO	J2	15	16	13	16	23	9	11	13	11	11	12	14	9	11	10	13	10	13,16
IP146	NPO	K(xP)	14	12	13	16	23	10	14	13	11	11	12	14	9	11	10	11	11	15,16
IP128	NPO	R1a1	15	12	13	20	26	11	11	13	11	11	12	14	11	10	11	11	11	11,14
IP142	NPO	R1a1	15	12	13	17	24	10	13	13	11	11	12	15	12	14	11	12	11	11,14
IP88	NPO	R1b3*	14	12	13	16	22	10	13	12	11	11	12	15	12	12	11	12	11	11,14
IP92	NPO	R1b3*	14	12	13	16	24	11	13	13	11	11	12	15	13	11	11	12	11	11,15
IP96	NPO	R1b3*	14	12	14	16	25	11	13	13	11	11	12	15	12	12	11	13	11	11,14
IP97	NPO	R1b3*	14	12	13	17	24	11	13	13	11	11	12	15	12	12	10	12	11	12,14
IP98	NPO	R1b3*	14	12	14	16	24	11	13	12	11	11	12	15	12	12	10	11	11	11,14
IP102	NPO	R1b3*	14	12	14	16	24	11	13	14	11	11	12	15	12	13	10	12	11	11,14
IP105	NPO	R1b3*	14	12	13	15	24	11	13	13	11	11	12	15	12	13	11	12	11	11,14
IP106	NPO	R1b3*	14	12	13	16	23	10	13	13	11	11	12	15	12	12	10	12	11	11,14
IP108	NPO	R1b3*	14	12	13	16	24	11	13	13	11	11	12	15	12	12	11	12	11	11,15
IP110	NPO	R1b3*	15	12	13	16	24	10	13	13	11	11	12	15	12	12	11	12	11	11,16
IP112	NPO	R1b3*	13	12	14	16	23	11	13	13	11	11	12	15	12	12	11	12	11	11,14
IP115	NPO	R1b3*	14	12	13	17	24	11	13	11	11	11	12	14	12	12	11	12	11	11,15
IP116	NPO	R1b3*	14	12	13	15	23	12	13	13	11	11	12	14	12	11	10	13	11	11,14
IP120	NPO	R1b3*	14	12	13	16	25	11	13	13	11	11	12	14	12	12	11	12	11	12,14
IP121	NPO	R1b3*	14	12	13	16	24	12	13	13	11	11	12	15	12	12	11	12	11	11,14
IP123	NPO	R1b3*	16	12	13	16	23	11	13	13	11	11	12	15	12	12	10	12	11	11,14
IP125	NPO	R1b3*	14	12	13	16	24	11	13	13	11	11	12	15	12	11	10	12	11	11,14
IP126	NPO	R1b3*	15	12	13	16	24	10	13	13	11	11	12	15	12	11	11	12	11	11,14
IP129	NPO	R1b3*	14	12	13	16	25	11	12	13	11	11	12	15	13	12	12	12	11	12,16
IP130	NPO	R1b3*	14	12	13	16	24	11	13	13	11	11	12	16	12	12	10	12	11	11,13

Name	Pop.	Hg	DYS19	DYS388	DYS389I	389 II-Ii	DYS390	DYS391	DYS392	DYS393	DYS434	DYS435	DYS436	DYS437	DYS438	DYS439	DYS460	DYS461	DYS462	DYS385
IP131	NPO	R1b3*	14	12	13	16	24	10	13	13	11	11	12	14	12	11	11	12	11	11,14
IP132	NPO	R1b3*	14	12	13	16	23	11	13	13	11	11	12	15	13	12	11	11	11	11,14
IP135	NPO	R1b3*	15	12	13	16	24	10	14	13	11	11	12	15	12	11	11	12	11	11,14
IP137	NPO	R1b3*	14	12	13	16	25	11	13	12	11	11	12	15	12	14	10	12	11	11,14
IP140	NPO	R1b3*	15	12	13	17	24	11	13	13	11	11	12	15	12	12	11	13	11	11,14
IP145	NPO	R1b3*	14	12	14	17	24	11	13	13	11	11	12	15	12	13	11	13	11	11,14
IP147	NPO	R1b3*	14	12	13	16	24	11	13	13	11	11	12	15	12	12	10	13	11	11,14
IP148	NPO	R1b3*	15	12	13	18	24	11	14	13	11	11	12	14	12	12	11	12	11	11,13
IP100	NPO	R1b3d	14	12	13	16	24	11	13	13	11	11	12	14	12	12	10	12	11	11,14
IP99	NPO	R1b3f	14	12	13	16	24	11	13	13	11	11	12	15	11	11	12	13	10	11,14
IP119	NPO	R1b3f	14	12	13	16	23	11	13	12	11	11	12	15	12	12	11	13	11	11,14
IP122	NPO	R1b3f	14	12	13	17	23	11	13	13	11	11	12	15	12	12	11	13	11	11,14
IP134	NPO	R1b3f	14	12	13	17	23	11	13	13	11	11	12	15	12	12	11	13	11	11,14
IP141	NPO	R1b3f	14	12	13	16	24	11	13	13	11	11	12	15	11	12	11	13	11	11,14
IP144	NPO	R1b3f	14	12	13	16	24	11	13	14	11	11	12	15	12	11	12	13	11	11,13
**Northwest Castille**																				
IP439	NWC	E3b*	15	12	13	16	22	9	11	14	11	11	12	14	10	11	11	12	12	14,15
IP963	NWC	E3b*	14	12	14	16	23	9	11	13	11	11	12	14	10	11,12	10	13	12	12,17
IP443	NWC	E3b1	13	12	13	17	24	10	11	13	11	11	12	14	10	12	9	12	11	15,18
IP450	NWC	E3b1	13	12	13	17	24	10	11	13	11	11	12	14	10	12	9	12	12	16,18
IP675	NWC	E3b1	14	12	13	17	25	10	11	13	11	11	12	14	10	14	11	12	12	18,19
IP678	NWC	E3b1	13	12	13	17	24	10	11	13	11	11	12	14	10	12	9	12	12	16,18
IP749	NWC	E3b1	13	12	13	17	24	10	11	13	11	11	12	14	10	12	10	12	12	17,17
IP960	NWC	E3b1	13	12	13	17	24	10	11	13	11	11	12	14	10	12	10	12	12	16,17
IP448	NWC	E3b2	13	12	14	16	24	9	11	13	11	11	12	14	10	10	11	13	12	13,14
IP451	NWC	E3b2	13	12	14	16	24	9	11	13	11	11	12	14	10	10	11	12	12	13,15
IP782	NWC	E3b2	13	12	14	16	24	9	11	13	11	11	12	14	10	10	10	13	12	14,14
IP828	NWC	E3b2	13	12	14	16	24	9	11	13	11	11	12	14	10	10	11	13	13	13,14
IP952	NWC	E3b2	13	12	13	16	24	9	11	13	11	11	12	14	10	10	11	13	12	13,14
IP956	NWC	E3b2	13	12	14	16	24	9	11	13	11	11	12	14	10	10	12	13	12	13,14
IP958	NWC	E3b2	13	12	14	16	24	9	11	13	11	11	12	14	10	10	11	13	12	13,14
IP971	NWC	E3b2	13	12	14	16	24	9	11	13	11	11	12	14	10	10	11	12	12	13,14
IP977	NWC	E3b2	13	12	14	16	24	9	11	13	11	11	12	14	10	10	11	13	12	12,14
IP980	NWC	E3b2	13	12	14	16	24	9	11	13	11	11	12	14	10	10	11	13	11	13,15
IP373	NWC	E3b3	13	12	12	18	24	10	11	13	11	11	12	14	10	12	11	13	11	15,19
IP228	NWC	G	15	12	11	17	22	10	11	14	11	11	12	16	10	11	10	12	12	13,14
IP377	NWC	G	15	13	12	16	22	10	11	14	11	11	12	16	10	11	10	11	12	14,15
IP454	NWC	G	15	13	12	17	22	10	11	14	11	11	12	16	10	11	10	11	12	14,14
IP455	NWC	G	15	12	13	16	23	11	11	14	11	11	12	16	10	11	11	12	12	14,15
IP752	NWC	G	15	12	13	17	22	11	11	13	11	11	12	15	9	10	10	11	12	14,14
IP876	NWC	I	17	13	13	15	23	10	11	13	11	11	12	15	10	12	10	11	12	12,12
IP966	NWC	I	15	13	15	17	23	11	12	14	11	11	12	14	10	11	11	12	12	15,16

Name	Pop.	Hg	DYS19	DYS388	DYS389I	389 II-Ii	DYS390	DYS391	DYS392	DYS393	DYS434	DYS435	DYS436	DYS437	DYS438	DYS439	DYS460	DYS461	DYS462	DYS385
IP987	NWC	I	14	14	12	16	22	11	11	13	11	11	12	16	10	11	10	12	12	12,13
IP967	NWC	J(xJ2)	13	15	12	17	24	10	11	12	11	11	12	14	10	12	10	11	11	12,17
IP243	NWC	J2	14	15	12	18	24	10	11	12	10	11	12	16	11	11	10	13	11	13,16
IP440	NWC	J2	14	15	13	17	23	10	11	12	11	11	12	15	9	12	10	13	11	13,20
IP441	NWC	J2	17	15	12	16	24	10	11	12	11	11	12	16	9	12	10	10	11	16,17
IP449	NWC	J2	14	14	13	17	24	10	11	12	11	11	12	15	9	12	11	15	11	15,15
IP741	NWC	J2	14	14	14	18	25	10	11	12	11	11	12	15	9	11	10	12	11	13,15
IP837	NWC	J2	15	16	13	16	23	9	11	12	11	11	12	14	9	11	10	13	10	13,16
IP860	NWC	J2	14	16	12	17	23	10	11	12	11	11	12	15	9	11	12	12	11	13,17
IP985	NWC	J2	16	15	12	16	25	10	11	12	11	11	12	16	9	11	11	10	11	14,16
IP248	NWC	K(xP)	15	11	14	16	23	10	13	13	11	11	12	14	9	11	11	11	12	14,16
IP309	NWC	K(xP)	13	12	13	17	23	9	11	13	11	11	12	14	10	12	10	13	12	12,15
IP959	NWC	R1a1	16	12	13	19	25	11	11	13	11	11	12	14	11	10	11	11	11	11,15
IP974	NWC	R1a1	15	12	14	17	25	10	11	12	11	11	12	14	11	10	11	12	11	11,14
IP232	NWC	R1b3*	14	12	13	16	24	11	14	13	11	11	12	14	12	12	10	13	11	11,12,14
IP247	NWC	R1b3*	14	12	13	16	24	11	13	13	11	11	12	15	12	12	11	13	11	12,14
IP269	NWC	R1b3*	16	12	13	16	24	11	13	13	11	11	12	15	12	12	11	12	11	11,14
IP284	NWC	R1b3*	14	12	14	16	25	10	13	13	11	11	12	15	12	12	11	12	11	11,14
IP288	NWC	R1b3*	14	12	13	16	24	10	13	13	11	11	12	15	12	12	12	11	11	11,14
IP290	NWC	R1b3*	14	12	13	16	23	10	13	13	11	11	12	14	12	11	11	12	11	11,14
IP306	NWC	R1b3*	14	12	14	16	24	10	13	13	11	11	12	15	12	11	11	12	11	11,14
IP337	NWC	R1b3*	14	12	14	17	24	11	13	13	11	11	12	14	12	12	10	11	11	11,14
IP343	NWC	R1b3*	14	12	12	17	24	11	13	13	11	11	12	16	12	12	10	12	11	11,14
IP353	NWC	R1b3*	15	12	13	16	24	10	13	13	11	11	12	15	12	13	11	12	11	11,14
IP355	NWC	R1b3*	14	12	13	15	24	11	13	13	11	11	12	15	12	13	11	11	10	11,14
IP356	NWC	R1b3*	14	12	13	16	24	11	13	13	11	11	12	15	12	13	10	12	11	11,13
IP360	NWC	R1b3*	14	12	13	16	23	11	13	12	11	11	12	15	12	12	11	11	11	11,11
IP363	NWC	R1b3*	14	13	13	16	23	10	13	14	11	11	12	15	12	12	11	12	11	11,14
IP382	NWC	R1b3*	14	11	13	16	24	11	14	13	11	11	12	15	12	11	11	12	11	11,14
IP438	NWC	R1b3*	14	12	13	16	24	10	13	13	11	11	12	15	13	11	11	12	11	11,17
IP442	NWC	R1b3*	14	12	12	16	23	11	13	13	11	11	12	15	12	12	11	12	11	11,14
IP444	NWC	R1b3*	14	12	13	17	24	11	13	13	11	11	12	14	12	12	11	12	11	12,14
IP445	NWC	R1b3*	14	12	13	16	23	11	13	13	11	11	12	14	11	12	11	12	11	11,15
IP452	NWC	R1b3*	14	12	13	16	24	11	13	13	11	11	12	15	12	12	10	12	11	11,14
IP453	NWC	R1b3*	14	13	12	15	25	10	13	13	11	11	12	15	12	11	10	11	11	12,14
IP687	NWC	R1b3*	14	12	13	16	23	11	13	13	11	11	12	15	12	11	11	12	11	11,14
IP709	NWC	R1b3*	14	12	14	16	24	10	13	13	11	11	12	14	12	11	10	14	11	11,13
IP711	NWC	R1b3*	14	12	13	17	22	11	13	13	11	11	12	15	13	12	10	12	11	11,14
IP767	NWC	R1b3*	14	13	13	15	25	10	13	13	11	11	12	16	12	12	10	11	11	12,14
IP786	NWC	R1b3*	14	12	14	16	25	10	13	13	11	11	12	15	12	12	11	12	11	11,14
IP792	NWC	R1b3*	14	12	13	15	22	10	13	13	11	11	12	15	11	11	11	12	11	11,16
IP804	NWC	R1b3*	14	12	13	16	24	10	13	13	11	11	12	15	12	11	11	12	11	13,14
IP805	NWC	R1b3*	14	12	13	16	24	11	13	13	11	11	12	15	12	11	11	12	11	12,14

Name	Pop.	Hg	DYS19	DYS388	DYS389I	389 II-Ii	DYS390	DYS391	DYS392	DYS393	DYS434	DYS435	DYS436	DYS437	DYS438	DYS439	DYS460	DYS461	DYS462	DYS385
IP821	NWC	R1b3*	14.5	12	13	17	24	11	13	13	11	11	12	15	12	11	11	12	11	12,15
IP830	NWC	R1b3*	15	12	13	16	24	11	13	14	11	11	12	15	12	12	10	12	11	11,15
IP843	NWC	R1b3*	14	12	14	15	24	11	13	12	11	11	12	15	12	12	11	11	11	11,14
IP851	NWC	R1b3*	13	12	13	17	24	10	13	13	11	11	12	14	12	12	11	12	11	11,14
IP866	NWC	R1b3*	14	12	13	16	24	11	13	13	11	11	12	14	13	12	10	12	11	11,14
IP869	NWC	R1b3*	14	12	12	16	24	10	13	13	11	11	12	15	12	11	10	12	11	11,14
IP948	NWC	R1b3*	14	12	14	16	23	11	13	14	11	11	12	14	12	12	11	12	11	11,14
IP949	NWC	R1b3*	14	12	13	17	24	12	13	13	11	11	12	15	12	11	10	12	11	11,15
IP950	NWC	R1b3*	14	12	14	16	24	10	13	13	11	11	12	15	12	12	10	12	11	11,14
IP951	NWC	R1b3*	14	12	13	16	25	10	13	13	11	11	12	15	12	12	10	12	11	11,14
IP953	NWC	R1b3*	14	12	13	17	24	10	13	13	11	11	12	15	12	11	10	12	11	11,15
IP954	NWC	R1b3*	13	12	13	16	25	12	13	13	11	11	12	15	12	13	11	12	11	11,14
IP955	NWC	R1b3*	13	12	15	16	24	11	13	13	11	11	12	14	12	12	10	12	11	11,14
IP961	NWC	R1b3*	14	12	14	17	24	11	13	13	11	11	12	14	12	11	10	12	11	11,15
IP962	NWC	R1b3*	14	12	13	16	24	10	13	13	11	11	12	15	12	12	11	12	11	11,14
IP964	NWC	R1b3*	14	12	14	16	25	11	13	13	11	11	12	14	12	12	10	12	11	11,14
IP965	NWC	R1b3*	13	12	13	17	24	10	13	13	11	11	12	15	12	12	11	11	11	11,13
IP968	NWC	R1b3*	14	12	10	16	23	10	13	13	11	11	12	15	12	12	11	12	11	11,14
IP969	NWC	R1b3*	14	12	12	16	24	11	13	13	11	11	12	15	12	13	11	12	11	11,14
IP970	NWC	R1b3*	14	12	13	16	24	10	13	13	11	11	12	15	12	11	11	12	11	11,14
IP972	NWC	R1b3*	14	12	13	16	24	10	13	13	11	11	12	14	12	12	10	12	11	11,14
IP973	NWC	R1b3*	14	12	14	16	24	10	13	13	11	11	12	14	12	12	11	12	11	11,14
IP975	NWC	R1b3*	15	12	13	16	24	10	11	13	11	11	12	14	12	12	11	12	11	14,14
IP976	NWC	R1b3*	14	12	13	16	24	11	13	13	11	11	12	15	12	13	10	12	11	11,15
IP978	NWC	R1b3*	14	12	13	16	24	11	13	13	11	11	12	15	12	14	11	12	11	11,14
IP979	NWC	R1b3*	14	12	15	16	24	11	13	13	11	11	12	14	12	12	10	12	11	12,14
IP981	NWC	R1b3*	14	14	13	17	25	11	13	13	11	11	12	15	12	13	11	12	11	12,14
IP982	NWC	R1b3*	16	12	12	16	23	10	13	13	11	11	12	15	12	11	10	12	11	11,14
IP986	NWC	R1b3*	14	12	14	17	24	11	12	13	11	11	12	14	12	11	10	12	11	11,13
IP957	NWC	R1b3d	14	12	13	16	24	11	13	13	11	11	12	14	12	12	10	12	11	11,14
IP983	NWC	R1b3f	14	12	12	16	23	11	13	13	11	11	12	15	12	12	11	12	12	11,14
**South Portugal**																				
IP160	SPO	E3a	17	12	13	17	21	11	11	13	11	11	12	14	11	11	11	13	12	17,17
IP206	SPO	E3b*	13	12	13	19	24	10	11	12	11	11	12	14	10	12	10	13	12	16,16
IP210	SPO	E3b*	14	12	14	19	25	10	12	14	11	11	12	14	11	13	10	12	12	17,18
IP168	SPO	E3b1	13	12	13	16	24	10	11	13	11	11	12	14	10	12	9	12	12	16,17
IP180	SPO	E3b1	13	12	13	17	23	10	11	13	11	11	12	14	10	11	11	12	12	15,15
IP198	SPO	E3b1	14	12	12	17	23	10	11	13	11	11	12	14	10	11	10	12	12	17,19
IP149	SPO	E3b2	13	12	14	16	24	9	11	13	11	11	12	14	10	10	11	13	12	13,14
IP152	SPO	E3b2	13	12	14	16	23	9	11	13	11	11	12	14	10	10	10	13	12	14,14
IP162	SPO	E3b2	13	12	14	16	24	9	11	13	11	11	12	14	10	10	11	13	12	14,14
IP174	SPO	E3b2	13	12	14	17	24	9	11	13	11	11	12	14	10	10	11	13	12	14,14

Name	Pop.	Hg	DYS19	DYS388	DYS389I	389 II-Ii	DYS390	DYS391	DYS392	DYS393	DYS434	DYS435	DYS436	DYS437	DYS438	DYS439	DYS460	DYS461	DYS462	DYS385
IP178	SPO	E3b2	13	12	13	16	24	9	11	13	11	11	12	14	10	10	12	12	11	11,14
IP201	SPO	E3b2	13	12	14	16	24	9	11	13	11	11	12	14	10	10	11	13	12	13,14
IP184	SPO	E3b3	13	12	13	17	24	11	11	13	11	11	12	14	10	13	10	11	11	16,16
IP159	SPO	G	15,16	12	14	16	22	10	11	14	11	11	12	16	10	10	11	13	12	13,16
IP177	SPO	G	15	12	13	17	21	10	13	14	11	11	14	16	10	13	11	11	12	13,16
IP186	SPO	G	15	12	12	16	22	10	11	14	11	11	12	16	10	13	12	11	12	12,16
IP202	SPO	G	15	12	12	16	22	9	11	14	11	11	12	16	10	13	12	11	12	12,16
IP205	SPO	G	15	12	12	18	23	10	11	14	12	11	12	16	10	12	11	11	12	14,15
IP219	SPO	G	15	12	12	17	22	10	11	14	11	11	12	16	11	12	11	10	12	15,15
IP222	SPO	G	15	12	14	16	22	10	11	14	11	11	12	17	10	10	11	12	12	13,16
IP158	SPO	I	16	15	13	18	25	10	12	14	11	11	12	15	10	13	10	11	12	12,15
IP172	SPO	I	16	13	13	15	22	11	11	13	11	11	12	15	10	12	11	11	12	12,12
IP223	SPO	I	16	13	13	15	23	10	11	13	11	11	12	14	10	12	10	11	13	12,12
IP167	SPO	J(xJ2)	14	16	13	18	23	10	11	12	11	11	12	14	10	11	11	12	11	13,19
IP192	SPO	J(xJ2)	14	16	13	17	23	10	11	12	11	11	12	14	10	14	11	11	11	13,15
IP156	SPO	J2	15	16	13	16	23	9	11	12	11	11	12	14	9	12	10	13	10	13,16
IP157	SPO	J2	14	15	13	17	24	10	11	12	10	11	12	16	7	12	11	13	11	13,15
IP164	SPO	J2	14	15	13	16	23	10	11	12	11	11	12	14	9	10	10	12	11	14,16
IP173	SPO	J2	14	15	13	17	22	10	11	12	11	11	12	15	9	11	10	14	11	13,15
IP179	SPO	J2	14	15	13	16	23	10	11	12	11	11	12	15	9	12	11	12	11	16,16
IP181	SPO	J2	15	15	12	16	25	10	11	12	11	11	12	16	9	11	11	10	11	14,17
IP183	SPO	J2	14	15	12	16	25	10	11	12	11	11	12	16	9	11	11	10	11	14,17
IP187	SPO	J2	15	15	12	17	23	10	11	12	11	11	12	15	9	12	10	10	11	14,17
IP199	SPO	J2	15	15	12	17	25	10	11	12	10	11	12	16	9	11	11	10	11	13,19
IP203	SPO	J2	14	15	14	17	23	10	11	12	11	11	12	14	9	11	9	13	11	13,16
IP213	SPO	J2	14	15	13	17	23	11	11	12	12	11	12	15	9	10	11	12	11	13,15
IP225	SPO	J2	14	15	13	17	21	10	11	12	11	11	12	15	9	11	10	14	11	13,15
IP154	SPO	K(XP)	13	12	14	16	22	10	13	13	11	11	12	15	9	11	10	11	12,13	13,13,14
IP163	SPO	K(xP)	13	12	13	16	23	10	13	14	11	11	12	15	9	11	11	11	13	15,16
IP211	SPO	K(xP)	13	12	14	15	24	10	13	13	11	11	12	14	9	12	10	12	12	13,15
IP220	SPO	K(xP)	13	12	14	15	24	10	13	13	11	11	12	14	9	12	10	12	12	13,15
IP226	SPO	R1a1	15	12	14	17	25	11	11	13	11	11	12	14	11	10	11	11	11	11,14
IP150	SPO	R1b3*	14	13	12	16	24	11	13	13	11	11	12	14	11	13	11	14	12	11,14
IP151	SPO	R1b3*	14	12	13	16	25	10	13	13	11	11	12	15	12	12	12	12	11	11,14
IP153	SPO	R1b3*	15	12	14	15	24	11	13	13	11	11	12	15	12	12	10	12	11	11,14
IP161	SPO	R1b3*	14	12	13	16	23	11	13	13	11	11	12	15	12	13	11	12	11	11,14
IP165	SPO	R1b3*	15	12	13	15	24	11	13	13	11	11	12	15	12	12	10	12	11	11,14
IP166	SPO	R1b3*	15	12	13	17	24	11	13	14	11	11	12	15	12	12	10	12	12	11,14
IP169	SPO	R1b3*	14	12	13	16	25	11	13	13	11	11	12	15	12	11	11	11	11	11,13
IP170	SPO	R1b3*	14	12	12	16	24	11	12	13	11	11	12	15	12	12	10	12	11	11,14
IP171	SPO	R1b3*	15	12	13	16	24	10	13	13	11	11	12	15	12	12	11	12	11	11,15
IP175	SPO	R1b3*	14	12	14	16	24	10	13	13	11	11	12	14	12	11	10	12	11	11,14
IP176	SPO	R1b3*	14	12	13	16	23	11	13	13	11	11	12	15	12	13	11	12	11	12,12

Name	Pop.	Hg	DYS19	DYS388	DYS389I	389 II-Ii	DYS390	DYS391	DYS392	DYS393	DYS434	DYS435	DYS436	DYS437	DYS438	DYS439	DYS460	DYS461	DYS462	DYS385
IP182	SPO	R1b3*	14	12	13	16	24	11	13	13	11	11	12	15	12	12	11	12	11	11,14
IP185	SPO	R1b3*	14	12	13	16	23	10	13	13	11	11	12	15	12	13	11	12	11	11,14
IP188	SPO	R1b3*	14	12	14	16	24	11	12	13	11	11	12	15	12	12	11	12	10	11,14
IP189	SPO	R1b3*	15	12	13	17	24	11	13	13	11	11	12	15	12	12	10	12	11	11,15
IP190	SPO	R1b3*	14	12	14	16	24	11	13	13	11	11	12	15	12	12	10	13	11	11,15
IP191	SPO	R1b3*	14	12	15	16	24	11	12	13	11	11	12	15	12	12	11	12	10	11,14
IP193	SPO	R1b3*	14	12	13	17	24	11	13	13	11	11	12	15	12	13	10	12	11	11,14
IP194	SPO	R1b3*	14	12	13	16	24	10	13	12	11	11	12	16	12	12	10	13	11	11,13
IP195	SPO	R1b3*	14	12	13	16	25	11	13	13	11	11	12	15	12	12	10	13	11	11,14
IP196	SPO	R1b3*	14	12	13	16	23	11	13	13	11	11	12	15	12	11	11	12	10	11,14
IP197	SPO	R1b3*	15	12	13	17	25	11	13	14	11	11	12	15	12	12	10	12	12	11,14
IP200	SPO	R1b3*	14	12	13	16	23	11	13	13	11	11	12	15	12	13	11	12	12	11,14
IP204	SPO	R1b3*	14	13	13	16	24	10	13	13	11	11	12	15	12	12	11	12	11	11,14
IP207	SPO	R1b3*	14	12	12	16	24	10	13	13	11	11	12	14	12	12	11	12	11	10,14
IP209	SPO	R1b3*	14	12	13	18	25	11	13	15	11	11	12	15	12	13	11	12	11	11,14
IP212	SPO	R1b3*	15	12	13	16	24	11	13	13	11	11	12	14	12	12	11	12	11	11,15
IP214	SPO	R1b3*	14	12	13	16	24	10	13	13	11	11	12	15	12	12	10	12	11	11,14
IP215	SPO	R1b3*	14	12	14	17	24	11	13	13	11	11	12	14	12	14	10	12	11	12,14
IP216	SPO	R1b3*	14	12	14	16	24	11	14	13	11	11	12	15	12	12	12	12	11	11,14
IP217	SPO	R1b3*	14	12	13	16	24	11	14	14	11	11	12	15	12	12	11	13	11	11,15
IP218	SPO	R1b3*	14	12	13	17	24	10	13	13	11	11	12	15	12	11	12	13	11	13,13
IP221	SPO	R1b3*	14	12	13	16	25	11	12	12	11	11	12	15	12	12	10	12	11	11,14
IP224	SPO	R1b3*	14	12	13	16	24	10	13	13	11	11	12	15	12	11	11	12	11	8,14
IP155	SPO	R1b3f	14	12	13	16	24	11	13	13	11	11	12	15	11	12	11	13	11	11,15
IP208	SPO	R1b3f	15	12	13	16	24	11	13	13	11	11	12	15	12	12	12	12	11	11,14
**Valencia**																				
IP474	VAL	E3a	16	12	13	18	21	11	11	14	11	11	12	15	11	12	10	13	12	17,17
IP609	VAL	E3b1	13	12	13	18	23	10	11	14	11	11	12	14	10	12	11	12	13	15,16
IP627	VAL	E3b1	13	12	13	17	24	10	11	13	11	11	12	14	10	11	9	12	13	15,18
IP629	VAL	E3b1	13	12	13	18	23	11	11	13	11	11	12	14	10	12	9	11	13	16,17
IP649	VAL	E3b1	13	12	13	17	24	10	11	13	11	11	12	14	10	11	9	12	12	16,16
IP471	VAL	E3b2	14	12	14	16	24	9	11	13	11	11	12	14	10	10	12	13	12	13,14
IP611	VAL	E3b2	13	10	14	16	24	9	11	13	11	11	12	14	10	10	11	13	12	13,14
IP644	VAL	E3b2	13	12	14	16	24	9	11	13	11	11	12	14	10	10	11	13	12	13,14
IP619	VAL	E3b3	13	12	14	18	23	10	11	13	11	11	12	14	10	12	10	12	11	15,18
IP459	VAL	G	15	13	12	16	22	10	11	15	11	12	12	16	10	11	11	11	12	14,15
IP465	VAL	I	15	13	13	16	23	11	12	14	11	11	12	15	10	12	10	12	12	15,15
IP614	VAL	I	14	14	12	17	23	10	11	13	11	11	12	16	10	11	10	12	12	13,15
IP623	VAL	I	17	13	14	15	23	10	11	13	11	11	12	15	10	11	10	11	12	12,12
IP641	VAL	I	14	14	12	15	22	10	11	13	11	11	12	17	10	11	11	12	12	13,14
IP647	VAL	I	14	14	12	16	22	10	11	13	11	11	12	16	10	11	9	12	12	13,14
IP658	VAL	I	17	13	14	16	23	10	11	13	11	11	12	15	10	12	10	12	12	12,12

Name	Pop.	Hg	DYS19	DYS388	DYS389I	389 II-Ii	DYS390	DYS391	DYS392	DYS393	DYS434	DYS435	DYS436	DYS437	DYS438	DYS439	DYS460	DYS461	DYS462	DYS385
IP660	VAL	I	14	14	12	16	23	10	11	13	11	11	12	16	10	11	10	12	12	13,14
IP470	VAL	J(xJ2)	14	17	14	17	23	11	11	13	11	11	13	14	10	10	11	12	12	11,18
IP630	VAL	J(xJ2)	14	16	12	18	23	10	11	12	11	11	12	14	10	11	11	11	11	12,17
IP464	VAL	J2	14	15	14	17	22	10	12	12	11	11	12	15	9	12	11	12	11	13,14
IP606	VAL	J2	13	15	13	16	22	11	11	12	11	11	12	15	9	11	11	13	11	13,16
IP612	VAL	J2	14	15	13	16	24	11	11	12	11	11	12	16	9	11	11	14	11	13,17
IP624	VAL	J2	14	15	14	17	23	10	11	12	11	11	12	14	9	11	9	13	11	13,16
IP469	VAL	K(xP)	14	12	13	15	23	10	13	13	11	11	12	14	9	11	10	11	13	13,16
IP631	VAL	R1a1	16	12	14	18	25	10	11	14	11	11	12	14	11	10	11	11	11	11,14
IP632	VAL	R1a1	15	12	13	18	25	11	11	13	11	11	12	14	11	10	12	11	11	11,14
IP456	VAL	R1b*	16	12	13	16	25	11	13	13	11	11	12	14	12	12	11	12	11	13,14
IP637	VAL	R1b*	17	12	13	15	22	11	13	13	11	11	12	14	11	12	11	12	11	13,14
IP271	VAL	R1b3*	15	12	12	16	25	11	14	13	11	11	12	15	12	12	10	13	11	11,14
IP457	VAL	R1b3*	14	12	13	16	24	10	13	13	11	11	12	15	12	12	11	12	11	11,14
IP458	VAL	R1b3*	14	12	14	17	25	11	13	13	11	11	12	14	12	11	10	13	11	11,14
IP461	VAL	R1b3*	14	12	14	16	24	11	13	14	11	11	12	15	12	12	10	12	11	11,15
IP462	VAL	R1b3*	14	12	13	16	23	12	13	13	11	11	12	14	12	13	10	12	11	11,15
IP467	VAL	R1b3*	14	12	13	16	23	11	13	13	11	11	12	15	12	12	10	12	11	11,14
IP472	VAL	R1b3*	15	12	13	16	24	10	13	13	11	11	12	15	12	13	11	12	11	11,14
IP473	VAL	R1b3*	14	12	14	17	24	10	13	13	11	11	12	14	12	12	10	11	11	11,14
IP475	VAL	R1b3*	14	12	14	16	24	11	13	13	11	11	12	15	12	11	11	12	11	11,14
IP607	VAL	R1b3*	14	12	13	16	23	10	13	13	11	11	12	15	12	12	10	12	11	12,14
IP608	VAL	R1b3*	14	12	14	16	24	11	13	13	11	11	12	15	12	12	11	12	11	11,14
IP610	VAL	R1b3*	14	12	13	16	23	12	13	13	12	11	12	14	12	12	10	12	11	11,14
IP613	VAL	R1b3*	14	12	12	16	24	10	13	13	11	11	12	15	12	12	11	12	11	10,16
IP615	VAL	R1b3*	14	12	13	16	24	10	13	13	11	11	12	14	12	13	11	12	11	11,14
IP616	VAL	R1b3*	14	12	13	16	24	11	13	12	11	11	12	14	12	12	10	12	11	13,14
IP618	VAL	R1b3*	14	12	13	15	24	11	13	14	11	11	13	15	12	12	11	12	11	11,14
IP620	VAL	R1b3*	14	12	13	16	24	10	13	13	11	11	12	15	12	12	11	12	11	11,14
IP621	VAL	R1b3*	14	12	13	16	24	11	13	13	11	11	12	15	12	11	11	12	11	11,15
IP622	VAL	R1b3*	16	12	14	16	25	10	11	13	11	11	12	14	11	11	11	11	11	11,15
IP625	VAL	R1b3*	14	12	13	16	24	11	13	13	11	11	12	15	12	12	11	12	11	11,14
IP626	VAL	R1b3*	14	12	13	16	24	11	13	13	11	11	12	15	12	12	10	11	11	11,14
IP633	VAL	R1b3*	14	12	13	17	23	11	13	13	11	11	12	14	12	11	11	10	11	11,13
IP634	VAL	R1b3*	14	12	14	15	24	10	13	13	11	11	12	15	12	11	11	12	11	12,14
IP636	VAL	R1b3*	14	12	13	15	24	11	12	13	11	11	12	15	12	12	11	12	11	11,14
IP639	VAL	R1b3*	15	12	13	16	24	10	13	13	12	11	12	15	12	12	11	12	11	11,14
IP640	VAL	R1b3*	16	12	13	17	23	10	13	13	11	11	12	15	12	13	11	12	11	11,13
IP642	VAL	R1b3*	14	12	13	16	26	11	13	13	11	11	12	15	12	13	10	12	11	11,15
IP645	VAL	R1b3*	14	12	14	16	24	11	13	13	11	11	12	14	9	12	10	12	11	11,14
IP646	VAL	R1b3*	14	12	13	16	24	10	13	13	11	11	12	15	12	12	10	12	11	12,14
IP650	VAL	R1b3*	15	12	13	16	24	10	13	14	11	11	12	15	12	13	11	12	11	11,14
IP652	VAL	R1b3*	14	12	13	16	23	10	13	13	11	11	12	15	12	12	11	12	11	11,14

Name	Pop.	Hg	DYS19	DYS388	DYS389I	389 II-I	DYS390	DYS391	DYS392	DYS393	DYS434	DYS435	DYS436	DYS437	DYS438	DYS439	DYS460	DYS461	DYS462	DYS385
IP656	VAL	R1b3*	14	12	13	16	24	11	14	13	11	11	12	15	12	13	10	14	11	11,13
IP657	VAL	R1b3*	14	12	13	17	25	11	13	12	11	11	12	14	12	14	11	13	11	11,14
IP659	VAL	R1b3*	14	12	13	16	24	11	13	13	11	11	12	15	12	12	11	12	11	11,15
IP662	VAL	R1b3*	14	12	13	16	24	11	13	13	11	11	12	14	12	12	11	12	11	11,14
IP663	VAL	R1b3*	14	12	13	17	24	10	13	13	11	11	12	15	12	12	11	12	11	11,14
IP693	VAL	R1b3*	14	13	13	16	24	10	13	13	11	11	9	15	12	13	11	12	11	11,14
IP697	VAL	R1b3*	14	12	14	17	25	11	13	13	11	11	12	15	12	13	11	12	11	12,14
IP700	VAL	R1b3*	15	12	13	16	24	11	13	13	11	11	12	15	12	13	11	12	11	10,15
IP707	VAL	R1b3*	14	12	13	17	24	11	13	13	11	11	12	16	12	11	11	12	11	11,14
IP468	VAL	R1b3d	14	12	14	16	24	11	13	13	11	11	12	14	12	11	10	11	11	10,13
IP617	VAL	R1b3f	14	12	13	15	24	11	13	13	11	11	12	15	12	13	11	12	11	11,15
IP655	VAL	R1b3f	15	12	13	15	24	11	13	13	11	11	12	15	12	11	11	12	11	11,14
IP664	VAL	R1b3f	14	12	13	16	24	11	12	14	11	11	12	15	12	11	11	12	11	10,14
IP814	VAL	R1b3f	14	12	13	16	24	11	13	13	11	11	12	15	12	12	11	12	11	11,14
**West Andalusia**																				
IP514	WAN	E3b*	13	12	12	17	24	10	11	14	11	11	12	15	10	10	11	13	12	16,18
IP272	WAN	E3b1	13	12	13	17	24	10	11	12	11	11	12	14	10	12	9	12	13	16,18
IP513	WAN	E3b1	13	12	13	17	24	10	11	13	11	11	12	14	10	12	10	12	12	16,18
IP759	WAN	E3b1	13	12	13	18	24	11	11	13	11	11	12	14	11	11	11	12	12	18,18
IP298	WAN	E3b2	13	12	14	16	24	9	11	13	11	11	12	14	10	10	10	13	12	13,14
IP515	WAN	E3b2	13	12	14	16	23	9	11	13	11	11	12	14	10	10	11	13	12	13,14
IP516	WAN	E3b2	13	12	14	16	24	9	11	13	11	11	12	14	10	9	11	13	12	13,15
IP791	WAN	E3b2	13	12	14	16	24	9	11	13	11	11	12	14	10	10	10	13	12	13,14
IP813	WAN	E3b2	13	12	14	16	23	10	11	13	11	11	12	14	10	11	11	13	12	13,14
IP852	WAN	E3b2	13	12	14	16	24	9	11	13	11	11	12	14	10	11	10	13	12	13,14
IP853	WAN	E3b2	13	12	13	16	24	9	11	13	11	11	12	14	10	10	11	13	12	13,14
IP674	WAN	G	15	13	13	17	22	10	11	15	11	11	12	16	11	11	10	11	12	14,14
IP696	WAN	G	15	12	12	18	23	11	11	14	11	11	12	16	10	11	11	12	12	13,15
IP839	WAN	G	15	13	13	17	22	10	11	15	11	11	12	16	10	11	10	11	12	14,14
IP374	WAN	I	16	13	13	15	23	10	11	13	11	11	12	15	10	11	10	11	12	12,12
IP517	WAN	I	17	13	12	17	25	10	11	13	11	11	12	15	10	12	10	12	12	12,16
IP754	WAN	I	16	13	12	15	25	11	11	13	11	11	12	15	10	11	10	12	12	13,17
IP783	WAN	I	15	13	13	17	24	10	12	14	11	11	12	15	10	11	13	12	12	15,15
IP797	WAN	J(xJ2)	14	16	14	16	23	10	11	12	11	11	12	14	10	11	11	11	11	13,15
IP279	WAN	J2	14	15	12	17	22	10	11	12	11	11	12	15	9	11	9	14	11	13,15
IP296	WAN	J2	15	15	12	16	24	10	11	12	11	11	12	16	10	12	11	10	11	12,18
IP666	WAN	J2	15	16	13	16	23	9	11	12	11	11	12	15	9	12	10	13	10	13,16
IP672	WAN	J2	16	15	13	16	24	11	11	12	11	11	12	15	9	11	10	13	11	12,16
IP690	WAN	J2	15	16	13	16	23	11	11	12	11	11	12	15	9	12	11	12	11	13,13
IP737	WAN	J2	15	15	13	17	23	10	11	12	11	11	12	15	9	12	10	14	11	13,17
IP738	WAN	J2	15	16	13	16	24	9	11	12	11	11	12	14	9	12	10	12	10	13,16
IP774	WAN	J2	15	16	13	16	23	9	11	12	11	11	12	14	9	11	11	12	8	13,16

Name	Pop.	Hg	DYS19	DYS388	DYS389I	389 II-Ii	DYS390	DYS391	DYS392	DYS393	DYS434	DYS435	DYS436	DYS437	DYS438	DYS439	DYS460	DYS461	DYS462	DYS385
IP316	WAN	J2	15	14	13	16	23	10	11	12	11	11	12	15	9	14	11	13	11	13,15
IP340	WAN	J2	15	16	13	16	24	9	11	12	11	11	12	14	9	12	10	12	10	13,16
IP246	WAN	Q(XQ3)	13	12	14	17	25	10	14	12	11	11	12	13	9	13	11	13	12	15,17
IP689	WAN	R1*	15	12	14	16	24	10	13	13	11	11	12	14	11	11	11	12	11	11,13
IP274	WAN	R1a1	16	12	14	17	25	10	11	13	11	11	12	14	11	10	11	11	12	11,14
IP350	WAN	R1a1	16	12	13	19	24	11	11	13	11	11	12	14	11	10	12	11	11	11,11
IP849	WAN	R1a1	15	12	13	17	25	11	11	13	11	11	12	14	11	11	12	11	11	11,14
IP230	WAN	R1b3*	14	12	12	16	23	11	13	14	11	11	12	14	12	12	10	13	11	10,14
IP236	WAN	R1b3*	14	12	14	18	24	11	13	14	11	11	12	15	12	11	11	12	11	11,14
IP240	WAN	R1b3*	14	12	13	16	24	11	13	13	11	11	12	15	12	12	11	12	11	11,14
IP241	WAN	R1b3*	14	12	13	16	23	10	13	13	11	11	12	15	12	13	11	12	11	11,14
IP249	WAN	R1b3*	14	12	13	17	23	10	13	13	11	11	12	15	12	12	10	12	11	12,15
IP260	WAN	R1b3*	14	12	12	16	24	11	13	13	11	11	12	15	10	13	10	12	11	11,14
IP291	WAN	R1b3*	14	12	14	17	24	10	13	13	11	11	12	14	12	12	9	12	11	11,14
IP301	WAN	R1b3*	14	12	13	15	24	11	13	13	11	11	12	15	12	11	10	12	11	11,14
IP310	WAN	R1b3*	14	12	13	16	25	11	13	13	11	11	12	15	12	11	12	12	11	11,14
IP313	WAN	R1b3*	14	12	13	16	23	10	13	13	11	11	12	15	12	11	11	12	11	11,14
IP315	WAN	R1b3*	14	12	13	16	24	10	13	13	11	11	12	15	12	12	11	12	11	11,14
IP327	WAN	R1b3*	14	12	13	16	24	11	13	13	11	11	12	15	12	12	10	12	11	11,14
IP328	WAN	R1b3*	14	12	13	16	25	10	13	13	11	11	12	15	12	13	11	12	11	11,11
IP331	WAN	R1b3*	14	12	12	16	23	11	14	13	11	11	12	14	12	11	11	12	11	11,14
IP380	WAN	R1b3*	14	12	13	16	24	11	13	13	11	11	12	15	12	12	11	12	11	11,12
IP486	WAN	R1b3*	14	12	14	16	24	11	13	12	11	11	12	15	12	13	11	11	11	11,14
IP489	WAN	R1b3*	14	12	12	16	24	11	13	13	11	11	12	15	12	12	11	12	11	11,14
IP493	WAN	R1b3*	14	12	14	16	24	11	13	13	11	11	12	14	12	11	10	13	11	11,14
IP495	WAN	R1b3*	14	12	13	16	24	11	13	13	11	11	12	15	12	12	11	12	11	11,14
IP512	WAN	R1b3*	14	12	13	16	25	10	13	13	11	11	12	15	12	13	11	12	11	11,15
IP654	WAN	R1b3*	14	13	12	16	24	10	13	13	11	11	12	15	12	12	11	12	11	11,14
IP670	WAN	R1b3*	14	12	13	16	24	11	13	13	11	11	12	15	12	12	11	13	11	11,14
IP686	WAN	R1b3*	14	12	13	17	25	11	13	13	11	11	12	15	12	12	11	12	11	11,14
IP701	WAN	R1b3*	15	12	13	16	23	11	13	13	11	11	12	15	12	12	10	12	11	11,14
IP713	WAN	R1b3*	14	12	13	16	24	10	13	13	11	11	12	14	12	12	11	12	12	11,16
IP726	WAN	R1b3*	14	12	13	16	24	11	13	13	11	11	12	14	12	12	11	12	11	11,14
IP757	WAN	R1b3*	14	12	14	17	24	10	13	13	11	11	12	14	12	13	10	12	11	11,14
IP762	WAN	R1b3*	14	12	13	16	24	11	13	12	11	11	12	14	12	12	10	12	11	11,16
IP768	WAN	R1b3*	14	12	13	16	23	10	13	13	11	11	12	15	12	12	11	12	11	11,16
IP776	WAN	R1b3*	15	12	13	16	24	11	13	13	11	11	12	14	12	14	11	12	11	11,17
IP780	WAN	R1b3*	14	12	14	17	25	10	13	13	11	11	12	14	12	12	11	12	12	11,15
IP825	WAN	R1b3*	14	12	13	16	24	10	13	13	10	11	12	14	12	12	10	11	11	11,15
IP840	WAN	R1b3*	14	12	14	15	24	10	13	13	11	11	12	14	12	12	10	12	11	12,15
IP841	WAN	R1b3*	15	12	13	16	24	10	13	13	11	11	12	15	12	12	10	12	11	11,15
IP850	WAN	R1b3*	14	12	13	16	24	11	14	13	11	11	12	15	12	12	10	12	12	12,15
IP275	WAN	R1b3d	14	12	13	16	24	10	13	13	11	11	12	14	12	12	10	12	11	11,14

Name	Pop.	Hg	DYS19	DYS388	DYS389I	389 II-Ii	DYS390	DYS391	DYS392	DYS393	DYS434	DYS435	DYS436	DYS437	DYS438	DYS439	DYS460	DYS461	DYS462	DYS385
IP810	WAN	R1b3d	14	12	13	18	24	10	13	14	11	11	12	14	12	12	11	12	11	11,14
IP283	WAN	R1b3f	14	12	12	16	24	11	13	13	11	11	12	15	12	12	10	12	11	11,15
IP688	WAN	R1b3f	14	12	13	16	24	11	13	13	11	11	12	15	12	13	10	12	11	11,14
**Ibiza**																				
IP1056	IBZ	E3b1	13	12	13	17	24	11	11	13	11	11	12	14	10	12	9	12	12	14,18
IP1077	IBZ	E3b1	13	12	13	17	24	11	11	13	11	11	12	14	10	12	10	12	12	14,18
IP1030	IBZ	E3b3	13	12	13	17	25	10	11	14	11	11	12	14	10	12	10	12	12	14,17
IP1050	IBZ	E3b3	13	12	13	17	25	10	11	14	11	11	12	14	10	13	10	12	12	14,17
IP1025	IBZ	G	15	12	12	16	22	10	11	14	11	11	12	16	10	13	10	12	12	14,14
IP1032	IBZ	G	15	12	12	16	22	10	11	14	11	11	12	16	10	13	10	12	12	14,14
IP1037	IBZ	G	15	13	12	17	22	10	11	14	11	11	12	16	10	12	10	11	12	14,14
IP1045	IBZ	G	15	12	12	16	22	10	11	14	11	11	12	16	10	13	10	12	12	14,14
IP1054	IBZ	G	15	13	12	17	22	10	11	14	11	11	12	16	10	12	10	11	12	14,14
IP1059	IBZ	G	15	12	12	16	22	10	11	14	11	11	12	16	10	13	10	12	12	14,14
IP1060	IBZ	G	15	13	12	17	22	10	11	14	11	11	12	16	10	12	10	11	12	13,14
IP1075	IBZ	I	16	13	13	19	24	11	11	13	11	11	12	15	10	13	10	11	12	14,15
IP1028	IBZ	J2	14	15	14	16	23	10	11	12	11	11	12	15	9	10	10	12	11	14,16
IP1042	IBZ	J2	14	15	14	17	23	10	11	12	11	11	12	16	9	10	10	12	11	14,16
IP1024	IBZ	K(xP)	14	12	14	16	23	11	13	13	11	11	12	14	9	11	10	11	12	17,17
IP1047	IBZ	K(xP)	14	12	14	16	23	12	13	13	11	11	12	14	9	11	10	11	12	17,17
IP1049	IBZ	K(xP)	14	12	14	16	23	11	13	13	11	11	12	14	9	11	10	11	12	17,17
IP1053	IBZ	K(xP)	14	12	14	16	23	11	13	13	11	11	12	14	9	11	10	11	12	17,17
IP1058	IBZ	K(xP)	14	12	14	16	23	12	13	13	11	11	12	14	9	11	10	12	12	17,17
IP1064	IBZ	K(xP)	14	12	14	16	23	12	13	13	11	11	12	14	9	11	10	11	12	17,17
IP1065	IBZ	K(xP)	14	12	14	16	23	11	13	13	11	11	12	14	9	11	10	11	12	17,17
IP1071	IBZ	K(xP)	14	12	14	16	23	12	13	13	11	11	12	14	9	11	10	11	12	17,17
IP1073	IBZ	K(xP)	14	12	14	16	23	11	13	13	11	11	12	14	9	11	10	11	12	17,17
IP1027	IBZ	R1b3*	15	12	13	16	23	11	13	13	11	11	12	15	12	12	11	12	11	11,14
IP1029	IBZ	R1b3*	14	12	13	16	24	11	13	13	11	11	12	15	13	12	11	12	11	12,14
IP1031	IBZ	R1b3*	14	12	13	16	24	10	13	13	11	11	12	14	12	13	11	12	11	11,14
IP1034	IBZ	R1b3*	14	12	13	17	24	11	13	10	11	11	12	15	12	12	11	12	11	11,14
IP1036	IBZ	R1b3*	14	12	13	16	24	10	13	13	11	11	12	14	12	13	11	12	11	11,14
IP1038	IBZ	R1b3*	15	12	13	16	23	11	13	13	11	11	12	15	12	12	11	12	11	11,14
IP1039	IBZ	R1b3*	16	12	13	16	23	11	13	13	11	11	12	15	12	12	11	12	11	11,14
IP1040	IBZ	R1b3*	14	12	13	16	24	11	13	13	11	11	12	15	12	12	11	12	12	11,14
IP1041	IBZ	R1b3*	14	12	13	17	24	11	13	13	11	11	12	14	12	12	11	12	11	11,14
IP1043	IBZ	R1b3*	14	12	13	17	24	11	13	13	11	11	12	14	12	12	11	12	11	11,14
IP1044	IBZ	R1b3*	15	12	13	17	24	11	13	13	11	11	12	14	12	12	11	12	11	11,14
IP1046	IBZ	R1b3*	16	12	13	16	23	10	13	13	11	11	12	15	12	12	11	12	12	11,14
IP1051	IBZ	R1b3*	14	12	14	16	24	10	13	13	11	11	12	14	12	12	10	12	11	11,14
IP1052	IBZ	R1b3*	15	12	13	16	24	11	15	13	11	11	12	15	12	13	11	12	11	11,14
IP1057	IBZ	R1b3*	14	12	13	16	24	11	13	10	11	11	12	14	12	12	11	13	11	11,14

Name	Pop.	Hg	DYS19	DYS388	DYS389I	389 II-Ii	DYS390	DYS391	DYS392	DYS393	DYS434	DYS435	DYS436	DYS437	DYS438	DYS439	DYS460	DYS461	DYS462	DYS385
IP1061	IBZ	R1b3*	14	12	13	16	24	11	13	13	11	11	12	15	12	13	11	12	11	11,14
IP1062	IBZ	R1b3*	14	13	14	16	24	10	13	13	11	11	12	14	12	12	10	12	11	11,14
IP1063	IBZ	R1b3*	14	13	14	16	24	10	13	13	11	11	12	14	12	12	10	12	11	11,14
IP1066	IBZ	R1b3*	14	12	13	16	25	10	13	13	11	11	12	15	13	12	11	12	11	12,14
IP1068	IBZ	R1b3*	14	12	13	16	24	11	13	13	11	11	12	15	12	12	11	12	12	11,14
IP1069	IBZ	R1b3*	14	12	13	16	24	11	13	10	11	11	12	15	12	12	12	12	11	11,14
IP1070	IBZ	R1b3*	14	12	13	16	24	10	13	13	11	11	12	14	12	13	11	12	11	11,14
IP1072	IBZ	R1b3*	14	12	14	16	24	10	13	13	11	11	12	14	12	12	10	12	11	11,14
IP1074	IBZ	R1b3*	15	12	13	16	23	10	13	13	11	11	12	15	12	11	11	12	11	11,14
IP1076	IBZ	R1b3*	15	12	13	18	24	9	13	13	11	11	12	14	12	11	10	12	11	11,14
IP1033	IBZ	R1b3d	15	12	13	16	24	11	13	13	11	11	12	14	12	13	10	12	11	11,14
IP1035	IBZ	R1b3d	15	12	13	16	24	11	13	13	11	11	12	14	12	12	10	12	11	11,14
IP1026	IBZ	R1b3f	14	12	14	16	25	11	13	13	11	11	12	15	12	12	11	12	11	11,14
IP1048	IBZ	R1b3f	14	12	13	16	24	11	13	13	11	11	12	15	12	11	10	12	11	11,14
IP1055	IBZ	R1b3f	14	12	13	16	24	11	13	13	11	11	12	15	12	11	10	12	11	11,14
IP1067	IBZ	R1b3f	14	12	14	16	25	11	13	13	11	11	12	15	12	12	11	12	11	11,14
**Majorca**																				
IP1139	MAJ	E3a	15	12	13	18	21	10	11	14	11	11	12	14	11	11	11	13	12	16,18
IP1081	MAJ	E3b1	14	12	13	17	24	10	13	13	11	11	12	14	10	13	11	12	13	16,19
IP1090	MAJ	E3b1	13	12	13	17	24	10	11	13	11	11	12	14	10	11	9	12	12	16,17
IP1091	MAJ	E3b1	14	12	14	18	24	10	11	13	11	11	12	14	10	11	11	12	12	17,18
IP1121	MAJ	E3b2	13	12	14	16	24	9	11	13	11	11	12	14	10	10	11	13	12	13,14
IP1084	MAJ	G	15	12	12	17	23	10	12	12	11	11	12	16	10	11	10	13	13	15,15
IP1085	MAJ	G	16	12	12	16	21	11	11	13	11	11	12	16	10	11	10	11	12	13,15
IP1088	MAJ	G	15	12	12	16	21	10	11	15	11	11	12	15	10	11	10	11	13	13,16
IP1097	MAJ	G	15	13	12	17	22	10	11	14	11	11	12	16	10	11	10	11	12	14,14
IP1089	MAJ	I	14	14	12	16	23	10	11	13	11	11	12	16	10	11	10	12	12	13,15
IP1093	MAJ	I	17	13	15	15	25	9	11	13	11	11	12	15	10	12	11	12	12	12,12
IP1095	MAJ	I	14	14	12	16	22	10	11	13	11	11	12	15	10	12	10	12	12	13,14
IP1129	MAJ	I	14	14	12	16	23	10	11	13	11	11	12	16	10	11	10	12	12	13,15
IP1137	MAJ	I	15	13	12	16	25	10	11	13	11	11	12	15	10	11	10	14	12	13,17
IP1099	MAJ	J(xJ2)	14	17	13	18	23	10	11	12	11	11	12	14	10	12	11	12	11	13,17
IP1080	MAJ	J2	15	15	12	16	23	10	11	12	11	11	12	15	9	11	11	10	11	14,18
IP1092	MAJ	J2	16	15	13	16	23	10	12	12	11	11	12	14	9	12	10	13	10	13,17
IP1120	MAJ	J2	16	15	14	16	23	10	12	12	11	11	12	14	9	12	11	13	10	13,17
IP1127	MAJ	J2	14	15	12	16	23	10	11	12	11	11	12	16	9	11	11	10	11	14,17
IP1134	MAJ	J2	15	16	13	16	23	9	11	12	11	11	12	14	9	11	10	13	10	13,16
IP1115	MAJ	K(xP)	14	12	13	16	25	11	13	13	11	11	12	14	12	11	11	13	11	11,14
IP1078	MAJ	R1b3*	14	12	13	16	24	10	13	13	11	11	12	15	12	12	11	12	11	11,14
IP1079	MAJ	R1b3*	14	12	13	16	25	11	13	13	11	11	12	15	12	12	10	12	11	11,15
IP1082	MAJ	R1b3*	14	12	13	16	24	11	13	13	11	11	12	14	12	11	11	12	11	11,14
IP1083	MAJ	R1b3*	15	12	13	16	23	11	13	13	11	11	12	14	12	12	12	13	11	10,15

Name	Pop.	Hg	DYS19	DYS388	DYS389I	389 II-Ii	DYS390	DYS391	DYS392	DYS393	DYS434	DYS435	DYS436	DYS437	DYS438	DYS439	DYS460	DYS461	DYS462	DYS385
IP1086	MAJ	R1b3*	15	12	13	16	24	11	13	13	11	11	12	15	12	12	12	11	11	11,15
IP1087	MAJ	R1b3*	14	12	13	16	24	11	13	13	11	11	12	14	12	11	11	12	11	11,14
IP1094	MAJ	R1b3*	14	12	13	16	24	11	13	13	11	11	12	15	12	12	11	12	11	8,14
IP1098	MAJ	R1b3*	15	12	13	16	24	11	13	13	11	11	12	14	13	12	11	12	11	11,14
IP1100	MAJ	R1b3*	14	12	13	17	24	10	13	13	11	11	12	15	12	11	12	13	11	11,14
IP1101	MAJ	R1b3*	15	12	13	16	24	10	14	13	11	11	12	15	12	12	10	12	11	11,14
IP1102	MAJ	R1b3*	14	12	14	16	23	10	13	13	11	11	12	15	12	13	11	13	11	11,11
IP1103	MAJ	R1b3*	14	12	14	16	24	11	12	13	11	11	12	14	12	12	10	12	11	11,14
IP1105	MAJ	R1b3*	14	12	15	16	23	11	14	13	11	11	12	15	12	12	11	12	11	11,14
IP1106	MAJ	R1b3*	14	12	12	18	25	10	13	13	11	11	12	14	12	12	11	12	11	11,14
IP1107	MAJ	R1b3*	14	12	13	17	24	11	13	13	11	11	12	15	12	12	11	12	11	11,14
IP1109	MAJ	R1b3*	14	12	13	16	24	11	13	13	11	11	12	14	12	11	11	12	11	11,15
IP1110	MAJ	R1b3*	14	12	13	16	24	11	13	13	11	11	12	15	12	11	11	12	11	11,14
IP1111	MAJ	R1b3*	15	12	14	18	24	11	13	13	11	11	12	15	12	12	11	11	11	11,14
IP1113	MAJ	R1b3*	16	12	13	16	23	10	13	13	11	11	12	14	12	12	11	14	11	10,14
IP1116	MAJ	R1b3*	14	12	13	16	24	11	13	13	11	11	12	15	13	11	11	12	11	13,17
IP1119	MAJ	R1b3*	14	12	13	16	221	11	13	13	11	11	12	14	12	11	11	13	11	11,14
IP1122	MAJ	R1b3*	14	12	12	16	24	10	13	13	11	11	12	15	12	12	11	12	11	12,14
IP1123	MAJ	R1b3*	14	12	13	16	24	12	13	13	11	11	12	14	12	12	11	11	11	11,14
IP1124	MAJ	R1b3*	15	12	13	16	22	11	13	13	11	11	12	15	12	12	12	12	11	11,14
IP1125	MAJ	R1b3*	14	12	13	16	24	11	13	12	11	11	12	14	12	12	11	12	11	11,14
IP1126	MAJ	R1b3*	14	12	13	16	24	11	13	13	11	11	12	15	13	11	11	12	11	13,17
IP1128	MAJ	R1b3*	16	12	13	16	23	10	13	13	11	11	12	15	12	12	11	12	12	11,14
IP1130	MAJ	R1b3*	14	12	13	16	24	11	13	13	11	11	12	14	12	11	11	12	11	11,15
IP1131	MAJ	R1b3*	14	12	13	16	24	11	13	13	11	11	12	14	12	11	11	12	11	11,14
IP1133	MAJ	R1b3*	13	12	14	16	23	11	13	13	11	11	12	15	12	11	11	12	11	11,14
IP1135	MAJ	R1b3*	14	12	13	16	24	11	13	13	11	11	12	15	13	11	11	12	11	13,17
IP1136	MAJ	R1b3*	14	12	13	16	24	11	13	13	11	11	12	14	12	12	10	12	11	12,15
IP1138	MAJ	R1b3*	14	12	13	16	24	11	13	14	11	11	12	14	12	12	10	12	11	11,14
IP1096	MAJ	R1b3f	14	12	13	16	24	10	13	13	11	11	12	15	12	12	11	12	12	11,14
IP1104	MAJ	R1b3f	14	12	13	16	24	11	13	13	11	11	12	15	12	13	11	12	11	11,14
IP1108	MAJ	R1b3f	14	12	13	16	25	11	13	13	11	11	12	14	12	12	11	12	11	11,14
IP1112	MAJ	R1b3f	13	12	12	16	24	11	13	13	11	11	12	15	12	11	11	12	11	11,14
IP1114	MAJ	R1b3f	14	12	13	16	24	10	12	14	11	11	12	15	12	11	11	12	11	11,14
IP1117	MAJ	R1b3f	14	12	13	16	24	10	12	14	11	11	12	15	12	11	11	12	11	11,14
IP1118	MAJ	R1b3f	14	12	13	16	24	10	12	14	11	11	12	15	12	11	11	12	11	11,14
IP1132	MAJ	R1b3f	14	12	13	16	24	10	12	14	11	11	12	15	12	11	11	12	11	11,14
**Minorca**																				
IP1157	MIN	E3b1	14	12	13	16	24	10	11	13	11	11	12	14	10	12	10	12	12	17,19
IP1160	MIN	E3b1	13	12	14	17	22	10	11	13	11	11	12	14	10	12	11	11	13	15,15
IP1168	MIN	E3b1	14	12	13	16	24	10	11	13	11	11	12	14	10	12	10	12	12	17,19
IP1175	MIN	E3b1	14	12	13	16	23	11	11	13	11	11	12	14	10	12	10	12	12	17,19

Name	Pop.	Hg	DYS19	DYS388	DYS389I	389 II-Ii	DYS390	DYS391	DYS392	DYS393	DYS434	DYS435	DYS436	DYS437	DYS438	DYS439	DYS460	DYS461	DYS462	DYS385
IP1140	MIN	E3b2	13	12	14	16	24	9	11	13	12	11	12	14	10	10	10	13	12	14,14
IP1152	MIN	E3b3	13	12	13	18	24	10	11	13	11	11	12	13	10	12	10	12	11	15,16
IP1162	MIN	E3b3	13	12	13	18	24	10	11	13	11	11	12	13	10	12	10	12	11	14,16
IP1144	MIN	I	16	13	13	15	23	10	11	13	11	11	12	15	10	13	10	11	12	12,12
IP1149	MIN	J2	16	15	13	19	22	10	11	12	11	11	12	14	9	11	10	13	11	13,15
IP1148	MIN	R1a1	16	12	14	16	25	11	11	13	12	11	12	14	12	10	10	11	11	11,14
IP1141	MIN	R1b3*	15	12	14	16	24	11	13	13	11	11	12	14	12	12	10	13	11	11,14
IP1145	MIN	R1b3*	14	12	13	16	24	11	13	13	11	11	12	14	12	12	11	12	11	11,14
IP1147	MIN	R1b3*	14	12	14	16	24	11	13	13	11	11	12	15	12	11	12	12	11	12,14
IP1150	MIN	R1b3*	14	12	13	16	23	11	13	12	11	11	12	15	12	12	11	11	11	11,14
IP1151	MIN	R1b3*	14	12	13	16	24	10	13	13	11	11	12	15	12	12	11	12	11	12,15
IP1154	MIN	R1b3*	14	12	13	17	24	11	13	13	11	11	12	15	12	13	10	12	11	11,14
IP1155	MIN	R1b3*	15	12	13	16	24	10	13	13	11	11	12	15	12	12	11	12	11	12,14
IP1156	MIN	R1b3*	15	12	13	16	23	11	13	13	11	11	12	14	12	11	11	12	11	11,14
IP1158	MIN	R1b3*	14	12	14	16	24	11	13	14	11	11	12	14	12	12	10	12	11	11,14
IP1159	MIN	R1b3*	14	12	14	17	24	10	13	13	11	11	12	14	12	12	10	12	11	11,14
IP1161	MIN	R1b3*	14	12	13	16	24	11	13	14	11	11	12	14	12	12	11	12	11	12,14
IP1163	MIN	R1b3*	14	12	12	16	24	11	13	13	11	11	12	15	12	12	11	12	11	12,13
IP1164	MIN	R1b3*	14	12	13	16	24	11	13	10	11	11	12	15	12	11	11	12	11	11,14
IP1165	MIN	R1b3*	14	12	13	17	24	11	13	13	11	11	12	15	12	13	11	12	11	11,14
IP1166	MIN	R1b3*	14	12	13	16	24	11	13	13	11	11	12	14	12	12	11	12	11	11,14
IP1167	MIN	R1b3*	14	12	14	16	24	11	13	14	11	11	12	14	12	12	10	12	11	11,14
IP1169	MIN	R1b3*	14	12	13	16	23	11	13	13	11	11	12	15	11	14	11	12	11	11,14
IP1170	MIN	R1b3*	14	12	13	16	24	11	13	13	11	11	12	14	12	12	11	12	11	11,14
IP1171	MIN	R1b3*	14	12	13	16	24	11	13	13	11	11	12	15	12	12	12	12	11	11,14
IP1173	MIN	R1b3*	14	12	13	17	24	10	13	13	11	11	12	15	13	13	11	12	11	11,13
IP1174	MIN	R1b3*	14	12	14	16	24	11	13	13	11	11	12	14	12	12	11	12	11	11,14
IP1176	MIN	R1b3*	14	12	13	16	23	11	13	13	11	11	12	15	12	12	11	13	11	11,14
IP1142	MIN	R1b3d	14	12	13	16	25	11	13	13	11	11	12	14	12	13	10	12	11	11,14
IP1143	MIN	R1b3f	14	12	13	16	24	10	12	14	11	11	12	15	12	11	11	12	11	11,14
IP1146	MIN	R1b3f	14	12	13	16	24	11	13	13	13	11	12	15	12	12	11	14	11	11,15
IP1153	MIN	R1b3f	14	12	13	16	24	11	13	13	12	11	12	15	12	12	11	12	11	11,15
IP1172	MIN	R1b3f	14	12	13	16	24	11	13	13	11	11	12	15	12	11	10	13	11	11,14
**Sephardic Jewish**																				
Sj1	SJ	E3a	13	14	13	16	23	10	11	13										
Sj2	SJ	E3b*	13	12	13	19	25	10	11	14										
Sj3	SJ	E3b*	13	12	14	18	23	10	11	12										
Sj4	SJ	E3b1	13	12	12	17	24	10	11	13										
Sj5	SJ	E3b1	13	12	12	17	23	10	11	13										
Sj6	SJ	E3b1	13	12	13	17	24	10	11	13										
Sj7	SJ	E3b1	13	12	12	18	23	10	11	13										
Sj8	SJ	E3b1	13	12	13	18	25	10	11	12										

Name	Pop.	Hg	DYS19	DYS388	DYS389I	389 II-Ii	DYS390	DYS391	DYS392	DYS393	DYS434	DYS435	DYS436	DYS437	DYS438	DYS439	DYS460	DYS461	DYS462	DYS385
Sj9	SJ	E3b1	13	12	13	17	24	10	11	13										
Sj10	SJ	E3b2	13	12	14	16	24	9	11	13										
Sj11	SJ	E3b3	14	12	13	17	24	9	11	14										
Sj12	SJ	E3b3	13	13	12	17	24	10	11	13										
Sj13	SJ	E3b3	13	13	12	17	24	10	11	13										
Sj14	SJ	E3b3	13	12	14	17	24	9	11	13										
Sj15	SJ	E3b3	14	12	13	17	24	9	11	14										
Sj16	SJ	E3b3	13	12	13	17	24	10	11	13										
Sj17	SJ	G	15	12	12	16	22	10	11	14										
Sj18	SJ	G	15	12	12	17	22	11	11	14										
Sj19	SJ	G	16	12	12	16	22	10	11	14										
Sj20	SJ	G	15	12	12	16	23	11	12	13										
Sj21	SJ	G	15	12	12	16	22	10	11	13										
Sj22	SJ	G	15	13	13	17	22	10	11	13										
Sj23	SJ	G	15	12	12	17	22	10	11	14										
Sj24	SJ	G	15	12	11	18	23	10	10	14										
Sj25	SJ	G	15	12	12	17	21	10	11	15										
Sj26	SJ	G	16	12	12	19	22	10	10	14										
Sj27	SJ	G	16	12	12	16	23	10	10	14										
Sj29	SJ	G	16	12	12	16	22	10	11	14										
Sj30	SJ	G	15	12	12	17	23	10	11	13										
Sj31	SJ	G	16	12	12	16	22	10	11	14										
Sj32	SJ	G	16	12	12	16	22	10	11	14										
Sj33	SJ	G	15	12	12	17	22	10	11	13										
Sj34	SJ	G	15	12	12	17	22	10	12	14										
Sj35	SJ	G	15	12	12	17	23	10	11	14										
Sj36	SJ	G	15	12	12	17	22	10	11	14										
Sj38	SJ	G	16	12	12	16	22	10	11	13										
Sj39	SJ	G	15	12	12	17	22	10	12	14										
Sj40	SJ	G	16	12	13	16	24	10	10	14										
Sj41	SJ	G	16	12	11	17	23	10	11	14										
Sj42	SJ	G	15	12	12	17	22	10	11	13										
Sj43	SJ	G	15	12	12	17	22	10	12	14										
Sj44	SJ	I	17	13	14	15	23	10	11	13										
Sj45	SJ	J(xJ2)	14	15	14	17	23	10	11	12										
Sj46	SJ	J(xJ2)	14	15	14	17	23	10	11	12										
Sj47	SJ	J(xJ2)	14	15	14	17	23	10	11	12										
Sj48	SJ	J(xJ2)	14	15	14	17	23	10	11	12										
Sj49	SJ	J(xJ2)	14	15	14	17	23	10	11	12										
Sj50	SJ	J(xJ2)	14	15	14	17	23	10	11	12										
Sj51	SJ	J(xJ2)	14	15	14	17	23	10	11	12										
Sj52	SJ	J(xJ2)	14	15	14	17	23	10	11	12										
Sj53	SJ	J(xJ2)	14	15	14	17	23	10	11	12										

Name	Pop.	Hg	DYS19	DYS388	DYS389I	389 II-Ii	DYS390	DYS391	DYS392	DYS393	DYS434	DYS435	DYS436	DYS437	DYS438	DYS439	DYS460	DYS461	DYS462	DYS385
Sj54	SJ	J(xJ2)	14	15	14	17	23	10	11	12										
Sj55	SJ	J(xJ2)	14	15	14	17	23	10	11	12										
Sj56	SJ	J(xJ2)	14	16	13	17	23	10	11	12										
Sj57	SJ	J(xJ2)	14	16	13	17	24	10	11	12										
Sj58	SJ	J(xJ2)	14	16	13	17	23	10	11	12										
Sj59	SJ	J(xJ2)	15	17	14	17	23	10	11	12										
Sj60	SJ	J(xJ2)	14	15	14	17	23	10	11	12										
Sj61	SJ	J(xJ2)	14	17	14	17	23	10	11	12										
Sj62	SJ	J(xJ2)	14	16	13	18	23	10	11	12										
Sj63	SJ	J(xJ2)	14	17	13	18	23	10	11	12										
Sj64	SJ	J(xJ2)	14	16	13	18	23	10	11	9										
Sj65	SJ	J(xJ2)	14	16	13	17	23	10	11	12										
Sj66	SJ	J(xJ2)	14	16	13	17	24	10	11	12										
Sj67	SJ	J(xJ2)	15	16	13	17	23	10	11	12										
Sj68	SJ	J(xJ2)	14	16	13	17	23	10	11	12										
Sj69	SJ	J(xJ2)	14	17	13	16	23	10	11	12										
Sj70	SJ	J(xJ2)	14	16	14	16	23	10	11	12										
Sj71	SJ	J(xJ2)	14	13	13	16	23	10	11	12										
Sj72	SJ	J(xJ2)	14	16	13	17	23	10	11	12										
Sj73	SJ	J(xJ2)	14	16	13	17	23	10	11	12										
Sj74	SJ	J(xJ2)	14	16	13	17	23	10	11	12										
Sj75	SJ	J(xJ2)	14	16	13	18	23	10	11	12										
Sj76	SJ	J(xJ2)	14	13	13	16	23	10	11	12										
Sj77	SJ	J(xJ2)	14	17	13	16	23	10	11	12										
Sj78	SJ	J(xJ2)	14	16	13	17	23	10	10	12										
Sj79	SJ	J(xJ2)	14	17	13	18	23	10	11	12										
Sj80	SJ	J(xJ2)	14	16	13	16	25	10	13	12										
Sj81	SJ	J(xJ2)	14	16	13	17	23	10	11	12										
Sj82	SJ	J(xJ2)	14	17	13	18	23	10	11	12										
Sj83	SJ	J2	14	16	14	16	24	9	11	12										
Sj84	SJ	J2	15	16	14	16	24	9	11	12										
Sj85	SJ	J2	15	16	14	17	24	9	11	12										
Sj86	SJ	J2	15	16	13	16	24	9	11	12										
Sj87	SJ	J2	15	16	14	16	24	9	11	12										
Sj88	SJ	J2	14	16	14	17	23	10	11	12										
Sj89	SJ	J2	15	16	14	16	23	10	11	12										
Sj90	SJ	J2	14	15	14	17	24	10	11	12										
Sj91	SJ	J2	15	16	14	16	23	10	11	12										
Sj92	SJ	J2	15	16	14	16	24	9	11	12										
Sj93	SJ	J2	15	16	14	17	24	9	11	12										
Sj94	SJ	J2	15	16	14	16	24	9	11	12										
Sj95	SJ	J2	17	15	13	16	23	10	11	12										
Sj96	SJ	J2	14	15	12	15	24	10	11	12										

Name	Pop.	Hg	DYS19	DYS388	DYS389I	389 II-Ii	DYS390	DYS391	DYS392	DYS393	DYS434	DYS435	DYS436	DYS437	DYS438	DYS439	DYS460	DYS461	DYS462	DYS385
Sj97	SJ	J2	15	15	12	16	24	11	11	12										
Sj98	SJ	J2	16	13	14	17	22	10	10	12										
Sj99	SJ	J2	14	14	13	17	24	10	12	12										
Sj100	SJ	J2	17	15	13	17	25	9	11	12										
Sj101	SJ	J2	15	17	13	17	23	10	11	12										
Sj102	SJ	J2	15	15	12	19	23	10	11	12										
Sj103	SJ	J2	14	14	13	17	24	10	11	13										
Sj104	SJ	J2	14	14	13	18	23	10	11	12										
Sj105	SJ	J2	14	14	13	17	24	10	12	12										
Sj106	SJ	J2	17	15	13	17	23	10	11	12										
Sj107	SJ	J2	14	14	13	17	22	10	11	12										
Sj108	SJ	J2	15	16	13	16	23	9	11	12										
Sj109	SJ	J2	16	15	13	16	23	9	11	12										
Sj110	SJ	J2	14	15	14	18	22	10	11	12										
Sj111	SJ	J2	14	16	13	16	23	10	11	12										
Sj112	SJ	J2	13	15	12	15	24	10	11	12										
Sj113	SJ	J2	15	15	12	16	24	11	11	12										
Sj114	SJ	J2	15	15	12	17	24	10	11	12										
Sj115	SJ	J2	15	16	13	16	23	9	11	12										
Sj116	SJ	J2	16	15	13	16	22	10	12	12										
Sj117	SJ	J2	15	16	14	16	23	10	11	12										
Sj118	SJ	J2	14	14	13	16	23	10	11	12										
Sj119	SJ	J2	15	14	13	16	23	10	11	12										
Sj120	SJ	J2	13	15	13	15	24	10	11	12										
Sj121	SJ	J2	14	15	14	16	23	10	11	12										
Sj122	SJ	J2	16	15	13	15	23	9	11	13										
Sj123	SJ	J2	16	15	13	16	22	10	12	12										
Sj124	SJ	J2	14	15	13	17	22	10	11	12										
Sj125	SJ	J2	13	15	14	19	23	10	11	12										
Sj126	SJ	K(xP)	15	12	13	17	23	11	13	13										
Sj127	SJ	K(xP)	14	12	13	17	23	10	13	13										
Sj128	SJ	K(xP)	13	12	13	15	22	10	13	13										
Sj129	SJ	K(xP)	15	12	13	16	23	11	13	13										
Sj130	SJ	K(xP)	14	12	14	15	23	10	13	13										
Sj131	SJ	K(xP)	14	12	13	18	23	10	13	13										
Sj132	SJ	K(xP)	15	12	13	17	23	11	13	13										
Sj133	SJ	K(xP)	13	12	13	16	22	10	13	13										
Sj134	SJ	K(xP)	13	12	13	15	22	10	13	13										
Sj135	SJ	K(xP)	14	12	14	16	23	10	14	12										
Sj136	SJ	K(xP)	15	12	15	15	23	10	14	11										
Sj137	SJ	Q(xQ3)	13	14	13	15	22	10	15	13										
Sj138	SJ	Q(xQ3)	13	12	13	16	22	10	15	13										
Sj139	SJ	Q(xQ3)	13	12	13	15	22	10	16	13										

Name	Pop.	Hg	DYS19	DYS388	DYS389I	389 II-Ii	DYS390	DYS391	DYS392	DYS393	DYS434	DYS435	DYS436	DYS437	DYS438	DYS439	DYS460	DYS461	DYS462	DYS385
Sj140	SJ	Q(xQ3)	13	12	13	14	22	10	15	13										
Sj141	SJ	R1a	15	12	13	16	24	11	11	13										
Sj142	SJ	R1a	16	12	13	17	25	10	11	13										
Sj143	SJ	R1a	16	12	12	18	25	11	11	13										
Sj144	SJ	R1a	15	12	13	17	25	10	11	12										
Sj145	SJ	R1a	16	12	13	17	25	10	11	14										
Sj146	SJ	R1a	16	12	13	17	24	10	11	13										
Sj147	SJ	R1a	16	12	13	17	25	10	11	13										
Sj148	SJ	R1a	16	12	13	17	24	10	11	13										
Sj149	SJ	R1b*	15	12	14	15	24	11	13	14										
Sj150	SJ	R1b*	16	12	13	16	23	10	13	13										
Sj151	SJ	R1b3	14	12	13	15	24	11	13	13										
Sj152	SJ	R1b3	14	12	13	15	24	11	13	13										
Sj153	SJ	R1b3	14	12	13	15	24	11	13	13										
Sj154	SJ	R1b3	14	12	13	16	24	11	13	13										
Sj155	SJ	R1b3	16	12	13	16	24	11	13	13										
Sj156	SJ	R1b3	14	12	13	16	24	10	14	13										
Sj157	SJ	R1b3	14	12	13	15	25	11	14	12										
Sj158	SJ	R1b3	14	12	13	15	24	11	13	13										
Sj159	SJ	R1b3	14	12	14	16	24	11	13	13										
Sj160	SJ	R1b3	14	12	12	15	24	10	14	12										
Sj161	SJ	R1b3	14	12	14	16	22	10	13	13										
Sj162	SJ	R1b3	14	12	13	15	24	10	14	12										
Sj163	SJ	R1b3	14	12	13	17	24	10	13	12										
Sj164	SJ	R1b3	14	12	12	15	24	11	14	12										
Sj165	SJ	R1b3	14	12	13	15	24	10	14	12										
Sj166	SJ	R1b3	14	12	13	16	25	11	13	13										
Sj167	SJ	R1b3	14	12	13	16	24	11	13	13										
Sj168	SJ	R1b3	14	12	13	16	24	11	13	13										
Sj169	SJ	R1b3	14	13	12	16	24	11	13	13										
Sj170	SJ	R1b3	14	12	13	15	25	10	14	13										
Sj171	SJ	R2	14	12	13	15	23	10	10	14										
Sj172	SJ	R2	14	12	14	15	23	10	10	14										
Sj173	SJ	R2	14	12	14	15	23	10	10	14										
Sj174	SJ	R2	14	12	14	15	23	10	10	14										

[a] "Hg" denotes "haplogroup."
[b] Note that subtyping of R1b3 was not done for the Sephardic Jewish sample.

Table S2. Ancestry Proportions in Iberian Populations

	Basque		Moroccan		Sephardic Jewish	
**Population**	**mY1 (%)**	**SD (%)**	**mY2 (%)**	**SD (%)**	**mY3 (%)**	**SD (%)**
**Aragon**	59.4	11.6	4.8	3.6	35.8	12.0
**Andalucia, E**	80.1	7.1	2.4	2.7	17.6	7.4
**Andalucia, W**	59.7	8.1	16.7	5.9	23.6	8.8
**Asturias**	44.3	15.3	10.5	7.3	45.2	16.2
**Castilla la Mancha**	81.1	8.3	0.9	2.7	18.0	8.7
**Castile, NE**	88.3	9.9	9.3	5.7	2.5	9.4
**Castile, NW**	65.4	6.0	21.7	5.4	12.9	2.7
**Catalonia**	91.5	7.0	2.3	2.5	6.2	7.1
**Extremadura**	52.3	9.4	19.0	7.1	28.7	10.4
**Galicia**	62.3	7.4	20.8	5.6	16.9	7.5
**Gascony**	110.8	4.7	-2.5	0.7	-8.3	4.7
**Portugal, N**	64.7	8.8	11.8	5.3	23.6	9.1
**Portugal, S**	47.6	8.1	16.1	6.1	36.3	9.2
**Valencia**	72.1	7.7	12.8	5.1	15.1	7.8
**Majorca**	71.9	8.5	6.6	4.3	21.5	8.7
**Minorca**	86.0	9.3	21.5	7.8	-7.5	7.3
**Ibiza**	63.2	9.0	3.8	4.3	33.0	9.7
